# Regional reef fish assemblage maps provide baseline biogeography for tropicalization monitoring

**DOI:** 10.1038/s41598-024-58185-6

**Published:** 2024-04-03

**Authors:** Brian K. Walker, Dana Fisco Becker, Gareth J. Williams, Audie K. Kilfoyle, Steven G. Smith, Allie Kozachuk

**Affiliations:** 1 GIS and Spatial Ecology Lab, Halmos College of Arts and Sciences, Nova Southeastern University, 8000 North Ocean Drive, Dania Beach, FL 33004 USA; 2 School of Ocean Sciences, Bangor University, Menai Bridge, Anglesey, LL59 5AB UK; 3 Resilient Environment Department, Broward County Florida, 115 S Andrews Ave, Room 329-H, Fort Lauderdale, FL 33301 USA; 4 Cooperative Institute for Marine and Atmospheric Studies, University of Miami’s Rosenstiel School of Marine, Atmospheric, and Earth Science, 4600 Rickenbacker Causeway, Miami, FL 33149 USA

**Keywords:** Western Atlantic, North America, Florida, Zoogeography, Community, Latitude, Subtropical, Biogeography, Climate-change ecology, Community ecology

## Abstract

The Anthropocene rise in global temperatures is facilitating the expansion of tropical species into historically non-native subtropical locales, including coral reef fish. This redistribution of species, known as tropicalization, has serious consequences for economic development, livelihoods, food security, human health, and culture. Measuring the tropicalization of subtropical reef fish assemblages is difficult due to expansive species ranges, temporal distribution shifts with the movement of isotherms, and many dynamic density-dependent factors affecting occurrence and density. Therefore, in locales where tropical and subtropical species co-occur, detecting tropicalization changes relies on regional analyses of the relative densities and occurrence of species. This study provides a baseline for monitoring reef fish tropicalization by utilizing extensive monitoring data from a pivotal location in southeast Florida along a known transition between tropical and subtropical ecotones to define regional reef fish assemblages and use benthic habitat maps to spatially represent their zoogeography. Assemblages varied significantly by ecoregion, habitat depth, habitat type, and topographic relief. Generally, the southern assemblages had higher occurrences and densities of tropical species, whereas the northern assemblages had a higher occurrence and density of subtropical species. A total of 108 species were exclusive to regions south of the Bahamas Fracture Zone (BFZ) (South Palm Beach, Deerfield, Broward-Miami) and 35 were exclusive to the north (North Palm Beach, Martin), supporting the BFZ as a pivotal location that affects the coastal biogeographic extent of tropical marine species in eastern North America. Future tropicalization of reef fish assemblages are expected to be evident in temporal deviance of percent occurrence and/or relative species densities between baseline assemblages, where the poleward expansion of tropical species is expected to show the homogenization of assemblage regions as adjacent regions become more similar or the regional boundaries expand poleward. Ecoregions, habitat depth, habitat type, and relief should be incorporated into the stratification and analyses of reef fish surveys to statistically determine assemblage differences across the seascape, including those from tropicalization.

## Introduction

As global temperatures rise, historically subtropical locales are at risk of becoming inhabited by range-extending vagrant tropical species in a process known as tropicalization^1–7^. Changes in large-scale thermoclines along continental coasts over the past three decades have affected the distribution of biological communities and may be facilitating the tropicalization of subtropical latitudes^1,3–8^. Tropicalization of marine taxa has been documented along Western Boundary Current coastlines with corals^9,10^ and reef fishes^11–13^ and is progressing poleward by 72 km per decade^14^, much faster than terrestrial fauna^3^. This redistribution of species has serious consequences for economic development, livelihoods, food security, human health, and culture and therefore must be incorporated into local, regional, and global assessments as standard practice to set achievable sustainability and biodiversity targets^3^.

Coral reef fishes comprise the most species-rich assemblages of vertebrates on earth^15,16^; however, over the past several decades, there have been substantial changes in the composition of the biomass and density of reef fish assemblages driven by many natural and anthropogenic factors^17–20^. Since water temperature is one of the most important abiotic factors influencing reef fish geographic distributions^21–23^, the warming climate has facilitated the range extension of non-native tropical species into subtropical reefs^24,25^. Such shifts have altered local assemblage structures^26^ and modified fish behavioral niches and interactions^27^. Severe reef fish biomass declines are predicted by 2100 under high ocean warming scenarios^28^, making understanding the spatial distributions of reef fish assemblages critical for management and conservation.

Determining reef fish tropicalization of subtropical and temperate reef systems is challenging because many species’ ranges are latitudinally expansive^29,30^, their distributions are correlated with depth^31^, they temporally shift with the movement of isotherms^32^, and a myriad of dynamic density-dependent factors affect their occurrence and density (e.g., competition, predation, recruitment)^33^. These factors vary along latitudinal gradients and have long been identified as zoogeographic indicators for the large-scale distribution and diversity of many coral reef fishes^14,30,34–43^. Ecotones form along these gradients where faunal latitudinal ranges overlap in transition zones between biogeographic provinces^32,44–46^. Species comingle in these zones to various extents depending on shifts in the oceanographic climate and seasonal changes^36^, local conditions^11^, and ecological processes^5,13,27,47^. To avoid these complexities, some studies focus on the new occurrence of vagrant species in poleward systems^6,7,48^, which illustrate a species becoming established in a new space through time. However, tropicalization may also be evident in changes to the relative density of historically co-occurring species with wide range distributions^26,49^.

Measuring regional changes in reef fish assemblages requires a baseline spatial categorization of reef fish assemblages throughout the entire bioregion^50,51^. Regional categorization of assemblages is valuable at multiple scales to better comprehend the processes of evolution, extinction, and biodiversity^51^, where a combination of abiotic variables (e.g., temperature, depth, topographic complexity) and ecological processes (e.g., recruitment, competition, food availability, predation) help determine distributions and relative abundance^52–56^. This baseline can then be used to compare against future datasets. Additionally, delineating the spatial distribution of reef fish assemblages provides a cost-effective and statistically appropriate survey design^57^, highlights critical areas of high conservation value and need^58,59^, provides appropriate strata for data analyses, and provides baseline data for evaluating the effects of management actions and climate change.

This study utilized extensive monitoring data from along a known transition between tropical and subtropical ecotones in southeast Florida to define regional reef fish assemblages, establish a baseline for monitoring reef fish tropicalization, and use benthic habitat maps to spatially represent their zoogeography. Since fish assemblages are known to vary locally with ecoregion, depth, habitat type, and topography, and benthic habitat map classifications include these descriptors in their scheme^57,60^, we used benthic habitat maps to spatially represent their extents. In doing so, we elicit a better understanding of the arrangement of reef fish communities along southeast Florida, provide a spatial framework and baseline data for monitoring reef fish tropicalization, support the Bahamas Fracture Zone as a pivotal location that affects the biogeographic extent of tropical marine species in eastern North America, and highlight the importance of including ecoregion differences in assemblage analyses.

## Methods

### Study area

The Florida Current is a western boundary current that travels along the mainland of the southeast and central Florida coast where the climate transitions from subtropical and temperate^44,45,61^ carrying tropical water northward and out to sea where it becomes known as the Gulf Stream. This current has long been recognized as a gateway facilitating the distribution of Western Atlantic tropical fishes to higher latitudes by the displacement of warmer waters up to the Carolinas and Bermuda^32,62,63^. The northern section of the Florida Reef Tract (nFRT) resides in a pivotal transitional area of the Florida Current where latitudinal and cross-shelf coastal benthic community and fish assemblage differences occur^40,64–66^. The nFRT consists of several linear, shore-parallel, coral reef assemblages separated from one another longitudinally by sand flats extending north from the Florida Keys for approximately 215 km^40,65,67^. Geologic coral reefs extend northward to the Bahamas Fracture Zone near Lake Worth before giving way to an expanded coastal shelf^65,68,69^ that facilitates upwelling associated with Florida Current meanders^70,71^. Prior studies have indicated that nFRT reef fish distributions are influenced by depth, topographic complexity, cross-shelf habitats^54^, and latitudinal setting^72–75^. Sailors’ choice (*Haemulon parra*), silver porgy (*Diplodus argenteus*), and hairy blenny (*Labrisomus nuchipinnis*) dominate the nearshore reef fish assemblages in Jupiter but are rare in Broward County (~ 80 km south)^72,75^. The seafloor in this region has been extensively mapped using multiple remote sensing techniques, and recent regional randomized reef fish surveys have been conducted, making it an ideal location to investigate coastal community biogeography^40,65,76,77^. Benthic habitat spatial analyses have indicated six coral reef ecoregions between southern Miami-Dade County and Martin County where the overall live stony coral cover and the size and number of distinct benthic habitats attenuate in a northward progression^40,64,65^; however, regional spatial biogeographic differences in reef fish communities have not been quantified.

### Data collection

**Figure 1:**
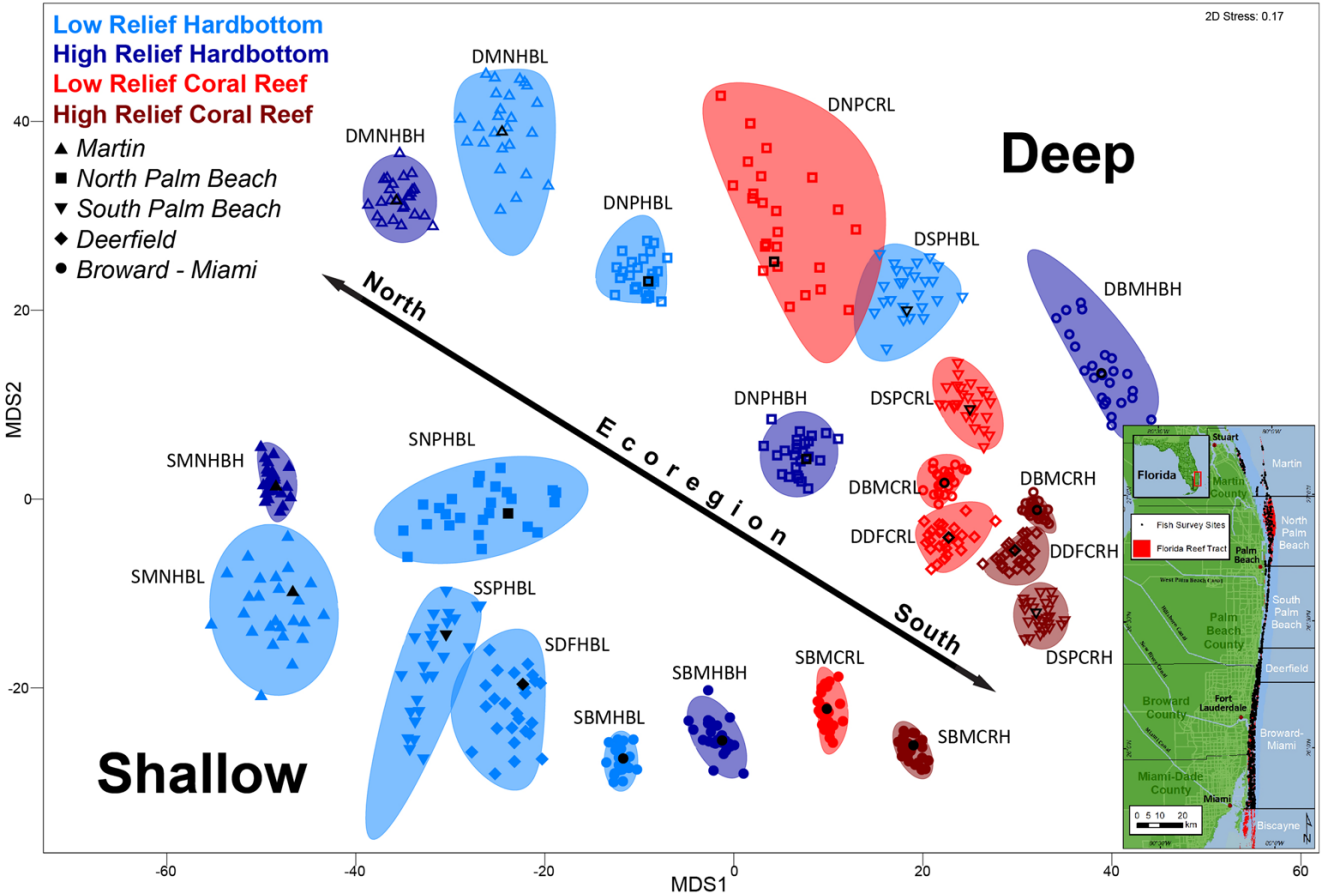
Bootstrap means plot of assemblage density data by habitat depth (shallow, deep), coral reef ecoregion (Broward-Miami, Deerfield, South Palm Beach, North Palm Beach, and Martin), habitat type (coral reef, hardbottom), and relief (high, low). Shallow sites are denoted by solid symbols, and deep sites are hollow. The symbol shape represents the ecoregion. Red represents coral reef habitats, and blue represents hardbottom habitats. Light colors are low relief, and dark colors are high relief. The map inset illustrates the study area, survey sites, and the coral reef ecosystem regions. Strata were abbreviated following the sequence depth-coral reef ecoregion-habitat type-relief. Depth: deep (D), shallow (S); Coral reef ecoregion: Broward-Miami (BM), Deerfield (DF), South Palm Beach (SP), North Palm Beach (NP), and Martin (MN); Habitat type: Hardbottom (HB), Coral Reef (CR); Relief: High (H), Low (L).

This study utilized data from annual diver surveys of the reef fish community conducted in the southeast Kristin Jacobs Coral Reef Ecosystem Conservation Area (Fig. 1) during the warmest months (May-Sept) of 2012–2014 (Table 1). The survey frame encompassed the full extent of mapped coral reef and hardbottom habitats shallower than 33 m^40,65^. The statistical sampling design was adapted from a stratified random survey utilized in the Florida Keys^57^. Field sampling collected biological data following a standard, nondestructive, in situ monitoring protocol in which a stationary diver records reef fish data (numbers-at-length of each species) while centered in a randomly selected circular plot 15 m in diameter^78^. Divers recorded the lowest possible taxon of all fish that enter the plot (from sea floor to sea surface) for five minutes. At which time the initial fishes seen were documented by total number and average, minimum, and maximum sizes for an additional fifteen minutes. Fish seen after the first five minutes were recorded separately. Each circular plot was sampled by a buddy pair of divers, and biological metrics (e.g., fish density, number per 177 m^2^) were computed as the arithmetic average of the stationary counts for a buddy team. For randomized site selection, the sample frame was gridded into 50-m cells and stratified by depth, habitat class, and topography within coral reef ecoregions defined by Walker^40^. Completed sites were plotted in GIS and categorized by depth, habitat type, and ecoregion according to their final location and diver data. Sites were classified by topography according to diver data.

**Table 1:** Number of surveys conducted in each map strata by year.

Habitat depth	Coral reef ecoregion	Habitat type	Relief	2012	2013	2014	Total
Deep	Martin	Hardbottom	Low	4	22	3	29
Deep	Martin	Hardbottom	High	0	15	33	48
Deep	North Palm Beach	Hardbottom	Low	17	78	61	156
Deep	North Palm Beach	Hardbottom	High	3	8	38	49
Deep	North Palm Beach	Coral Reef	Low	0	8	0	8
Deep	South Palm Beach	Hardbottom	Low	4	10	7	21
Deep	South Palm Beach	Coral Reef	Low	21	28	10	59
Deep	South Palm Beach	Coral Reef	High	11	28	42	81
Deep	Deerfield	Coral Reef	Low	44	43	1	88
Deep	Deerfield	Coral Reef	High	16	31	56	103
Deep	Broward-Miami	Hardbottom	High	2	5	4	11
Deep	Broward-Miami	Coral Reef	Low	90	65	13	168
Deep	Broward-Miami	Coral Reef	High	19	80	140	239
Shallow	Martin	Hardbottom	Low	6	2	6	14
Shallow	Martin	Hardbottom	High	4	6	36	46
Shallow	North Palm Beach	Hardbottom	Low	6	8	5	19
Shallow	South Palm Beach	Hardbottom	Low	4	10	4	18
Shallow	Deerfield	Hardbottom	Low	13	14	3	30
Shallow	Broward-Miami	Hardbottom	Low	104	115	22	241
Shallow	Broward-Miami	Hardbottom	High	4	8	61	73
Shallow	Broward-Miami	Coral Reef	Low	50	33	8	91
Shallow	Broward-Miami	Coral Reef	High	4	12	44	60
Total				426	629	597	1652

The surveys were funded to complement the National Oceanic and Atmospheric Administration’s National Coral Reef Monitoring Program (NCRMP) in the Florida Keys and adopted the same methods. It is important to note that the results herein describe the spatial distributions of assemblages during the rainy, warm season (May–Oct)^79^. The sample design precluded a seasonal analysis of the assemblages because surveys were not conducted during the dry, cool season. However, it is not uncommon for there to be a higher abundance of subtropical fish in southeast Florida during winter months^80^. A large-scale winter-time survey would be needed to understand how the spatial distribution of assemblages changes during colder months.

Habitat classifications from Walker^40^ were designated Coral Reef—a substrate that has historical coral reef growth^81^, and Hardbottom—every other type of natural, hard substrate habitat. Sites were categorized by the ecoregion in which they occurred from Walker and Gilliam^65^: Miami-Dade, Deerfield, South Palm Beach, North Palm Beach, and Martin. Walker, et al.^54^ showed 76% dissimilarity between reef fish assemblages in shallow habitats versus deep habitats as defined in Walker et al.^77^; therefore, the sites were categorized into two depth strata depending on the habitat in which they occurred: Shallow (< ~ 10 m) and Deep (~ 10 to ~ 33 m). Diver-estimated topographic relief was categorized using the NCRMP habitat stratification: low—sites with less than 30 cm maximum vertical relief; high—sites greater than 30 cm maximum vertical relief. Strata with less than 8 samples were removed from the analyses.

The thermal affinities of fish species were compiled from FishBase.org, a global biodiversity information system on finfishes covering > 35,400 fish species compiled from > 60,600 references in partnership with > 2510 collaborators^82^. Additionally, Aquamaps standardized distribution maps were used to reference species' ranges^83^. Species with limited latitudinal ranges were considered tropical and species with wider ranges into cold-water areas were considered subtropical.

### Data analysis

Reef fish abundance data by species for each site (all years combined for increased statistical power) were analyzed across four categorical factors using Plymouth Routines in Multivariate Ecological Research (PRIMER)^84^. Square root transformation was conducted to down weight the influence of highly dominant species on the observed patterns in multivariate space. A permutational multivariate analysis of variance (PERMANOVA)^85^ was used to test for an effect of coral reef ecoregion (fixed factor, 5 levels: Martin, North Palm Beach, South Palm Beach, Deerfield, Broward-Miami), depth (fixed factor, 2 levels: shallow, deep), reef topographic relief (fixed factor, 2 levels: high, low), and habitat type (random nested in ecoregion × depth × relief, 2 levels: coral reef, hardbottom) on reef fish assemblage structure. Following the global PERMANOVA test, pairwise tests were conducted to determine where any significant differences occurred between factor levels. All PERMANOVAs were conducted using the unrestricted permutation of raw data Type III partial sums of squares with 999 permutations. We then used nonmetric multidimensional scaling (nMDS) based on a Bray‒Curtis similarity matrix to visualize the relative similarity of fish assemblages across the factor levels and generated 95% bootstrapped averages for each factor level in multivariate space. This process was repeated without using ecoregion to illustrate the effect of not having defined ecoregions.

Benthic habitat polygons were combined based on the significant pairwise PERMANOVA results to define the spatial extent of reef fish assemblages in GIS. Similarity percentages of species (SIMPER), density, and frequency of occurrence data were tabulated for each assemblage. Similarity percentages identified the species contributing to the group similarities and to the differences between the reef fish assemblage strata. Species richness was calculated as the total number of species present at each data collection site. Nonparametric Kruskal‒Wallis ANOVA tests (*a* < 0.05) with a post hoc multiple comparison of mean ranks analysis were performed to test for total density and richness data differences between assemblages.

## Results

A total of 283,644 fish across 1652 sites were counted during three sampling seasons, representing 285 species from 66 families. The total mean fish density per site for all sites combined was 170.0 ± 5.9 standard error of the mean (SEM). The total mean species richness for all sites combined was 25.0 ± 0.2 SEM species/site.

There was a significant interaction between habitat type nested in ecoregion × habitat depth × relief (p = 0.001) on reef fish assemblage structure, indicating that all four factors significantly influenced reef fish assemblages (Table 2). Pairwise comparisons applied to this interaction term were largely significant: 94% (65/69) of the coral reef ecoregion comparisons, 88.9% (16/18) of the habitat depth comparisons, 90% (9/10) of the relief comparisons, and 71.4% (5/7) of the habitat type comparisons (Suppl. S1). The bootstrap averages nMDS plot corroborated the PERMANOVA results and showed clear separation of fish assemblages across all four factors and between levels within individual factors (Fig. 1). The main separation in the data was by habitat depth, where shallow habitats occupied one side of the plot and deep habitats occupied the opposite side. The plot also showed a latitudinal axis where the ecoregions generally plot in order of their geographic occurrence from north (left) to south (right) along the coast. High-relief sites plotted separately from low-relief sites of the same ecoregion, depth, and habitat type. Coral reef habitat sites grouped together by relief factors on the right side of the plot but remained distinct from each other.

**Table 2:** A summary of the PERMANOVA results testing habitat type (Ty) nested within coral reef ecoregion (Ec) × habitat depth (De) × relief (Re) in a mixed effect model.

Source	df	SS	MS	Pseudo-F	P (perm)	Unique perms
**Ec**	**4**	**2.14E+05**	**53,449**	**5.0107**	**0.006**	**997**
**De**	**1**	**79,764**	**79,764**	**8.0983**	**0.002**	**999**
Re	1	23,739	23,739	2.0843	0.133	999
EcxDe	4	66,301	16,575	1.5545	0.211	998
EcxRe	4	35,362	8840.6	0.61293	0.822	998
DexRe	1	6830.1	6830.1	0.63116	0.632	999
EcxDexRe*	1	7518.5	7518.5	0.69478	0.636	999
**Ty(EcxDexRe)**	**5**	**78,285**	**15,657**	**7.8988**	**0.001**	**995**
Res	1630	3.23E+06	1982.2			
Total	1651	4.29E+06				

Bold indicates significant results.

*Term has one or more empty cells.

**Figure 2:**
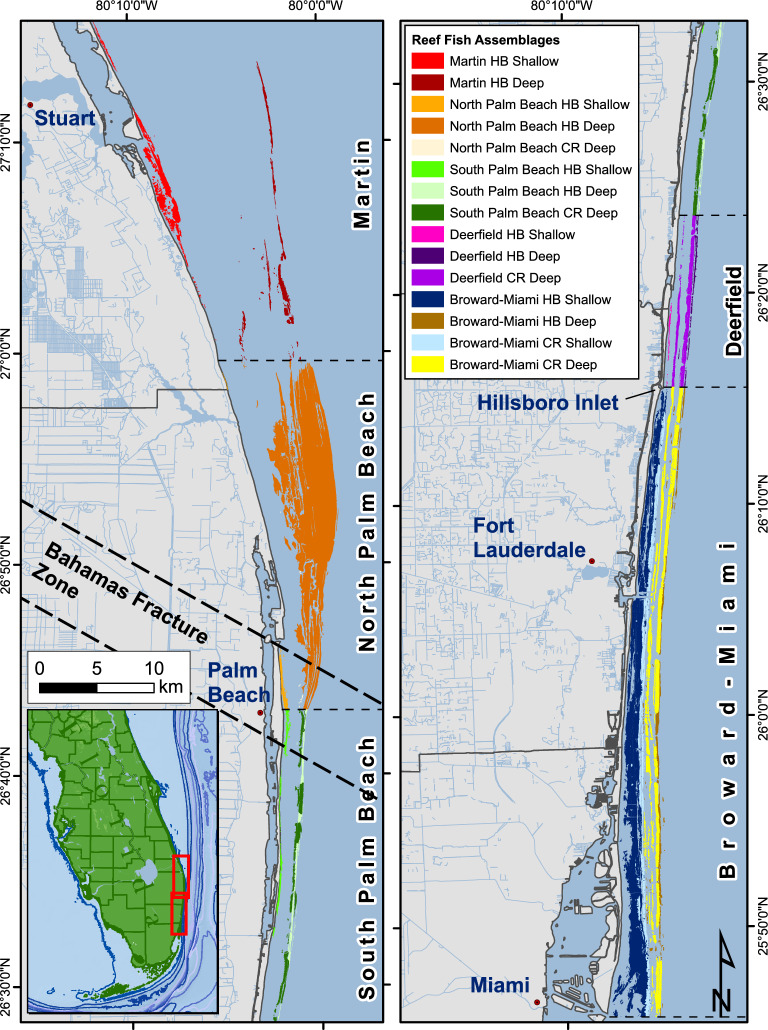
Map illustrating the final Reef Fish Assemblage Regions using the benthic habitat map polygons from Walker (2012) and Walker and Gilliam (2013). The coral reef ecoregions (Broward-Miami, Deerfield, South Palm Beach, North Palm Beach, and Martin) are labeled, and the divisions are indicated by dashed lines. HB-hardbottom habitats; CR-coral reef habitats.

Reef fish assemblages were defined as a combination of ecoregion, habitat depth, habitat types, and relief. Strata were abbreviated following the sequence depth-coral reef ecoregion-habitat type-relief. Depth: deep (D), shallow (S); Coral reef ecoregion: Broward-Miami (BM), Deerfield (DF), South Palm Beach (SP), North Palm Beach (NP), and Martin (MN); Habitat type: Hardbottom (HB), Coral Reef (CR); Relief: High (H), Low (L). Since relief has not been delineated in the benthic habitat polygons, ecoregion, habitat depth, and reef type were combined to create the assemblage maps (Fig. 2). Relief categorization needs to be included in future habitat maps to fully illustrate the spatial extent of the assemblages.

**Figure 3:**
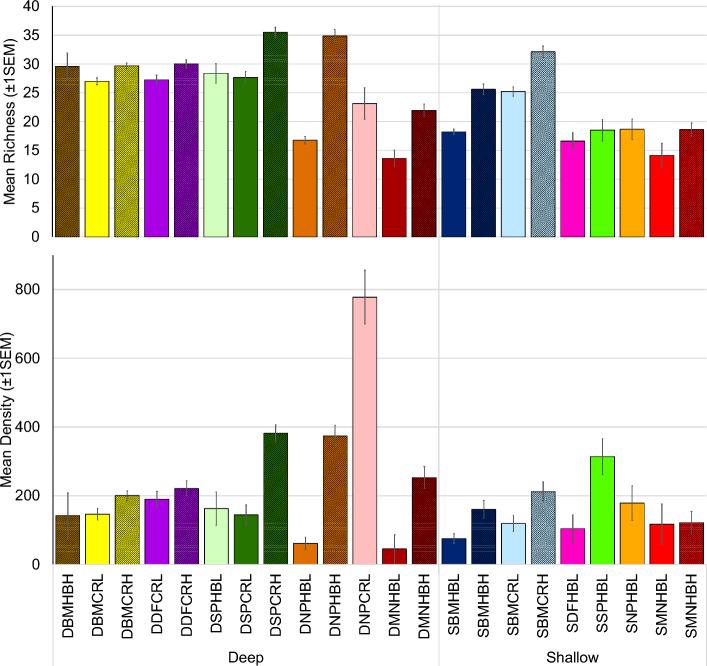
Mean species richness (top) and mean fish density (bottom) by reef fish assemblage regions. Error bars indicate one standard error of the mean. Strata were abbreviated following the sequence depth-coral reef ecoregion-habitat type-relief. Depth: deep (D), shallow (S); Coral reef ecoregion: Broward-Miami (BM), Deerfield (DF), South Palm Beach (SP), North Palm Beach (NP), and Martin (MN); Habitat type: Hardbottom (HB), Coral Reef (CR); Relief: High (H), Low (L).

Every high-relief assemblage of the same ecoregion, habitat depth, and habitat type had significantly higher mean richness than its low-relief equivalent and in every case for mean density as well except for the shallow Martin hardbottom (Fig. 3, Suppl. S2). Differences of total richness and density between deep and shallow habitats by ecoregion, habitat type, and relief were fewer (Suppl. S3). Richness was higher in the shallow Broward-Miami coral reef high relief (32.1 ± 0.9) versus the deep (29.7 ± 0.4) (p = 0.005), and higher in the deep South Palm Beach hardbottom low relief (28.4 ± 1.1) versus the shallow (18.5 ± 2.1) (p = 0.0008). All other richness depth comparisons were not significant. Total density was higher in the shallow North Palm Beach hardbottom low relief (178.7 ± 54.9) versus the deep (61.5 ± 5.6) (p = 0.002), in the shallow Martin hardbottom low relief (117.3 ± 26.0) versus the deep (45.8 ± 10.1) (p = 0.003), and in the deep Martin hardbottom high relief (252.8 ± 54.5) versus the shallow (121.8 ± 17.3) (p = 0.025). Richness and density on coral reef assemblages was significantly higher in three of the five comparisons to hardbottom in the same ecoregion, depth, and relief (Suppl. S4).

In deep coral reef high relief assemblages, density (d) and richness (r) was higher South Palm Beach (d = 382 ± 39.6; r = 35.5 ± 0.9) than in Deerfield (d = 221.2 ± 16.9; r = 30 ± 0.7) and Broward-Miami (d = 200.3 ± 9.1; r = 29.7 ± 0.4) (p < 0.001) and density was higher in Deerfield (189.8 ± 19.4) versus Broward-Miami (146.3 ± 9.2) in deep coral reef low relief (p = 0.048) (Suppl. S5). In deep hardbottom high relief assemblage density was higher in North Palm Beach (373.9 ± 51.5) than Martin (252.8 ± 54.5) and Broward-Miami (142.3 ± 193) (p > 0.02), while richness was lower in Martin (21.9 ± 1.4) versus North Palm Beach (34.9 ± 1.4) and Broward-Miami (29.5 ± 1.4) (p < 0.005). In deep hardbottom low relief assemblages, South Palm Beach had higher density and richness (d = 162.5 ± 19.4; r = 28.4 ± 1.1) than Martin (d = 45.8 ± 10.1; r = 13.6 ± 1.2) and North Palm Beach (d = 61.5 ± 5.6; r = 16.8 ± 0.7) (p < 0.001).

In the shallow habitats, richness and density were only different in a few of the hardbottom comparisons (Suppl. S5). In shallow hardbottom low relief assemblages, density was lower in Broward-Miami (75.4 ± 4.6) than North Palm Beach (178.7 ± 54.9) and South Palm Beach (313.6 ± 147.5) (p < 0.04). However, richness in Broward-Miami shallow hardbottom low relief (18.2 ± 0.5) and high relief assemblages (25.6 ± 1) was higher than Martin (low = 14.1 ± 1.4; high = 18.6 ± 0.8) (p < 0.03).

The percent occurrence and density of species within depth, habitat, and relief categories between assemblage regions showed latitudinal distribution patterns. A total of 108 species were exclusive to regions south of the Bahamas Fracture Zone (BFZ) (South Palm Beach, Deerfield, Broward-Miami) and 35 were exclusive to the north (North Palm Beach, Martin). Of the 231 species found in the shallow assemblages, 80 were exclusive to regions south of the BFZ, and 13 were exclusive to areas north of the BFZ (Suppl. S6, S7). Of the 270 species found in the deep assemblages, 44 were exclusive to regions south of the BFZ and 26 were exclusive to areas north of the BFZ (Suppl. S8, S9).

**Figure 4:**
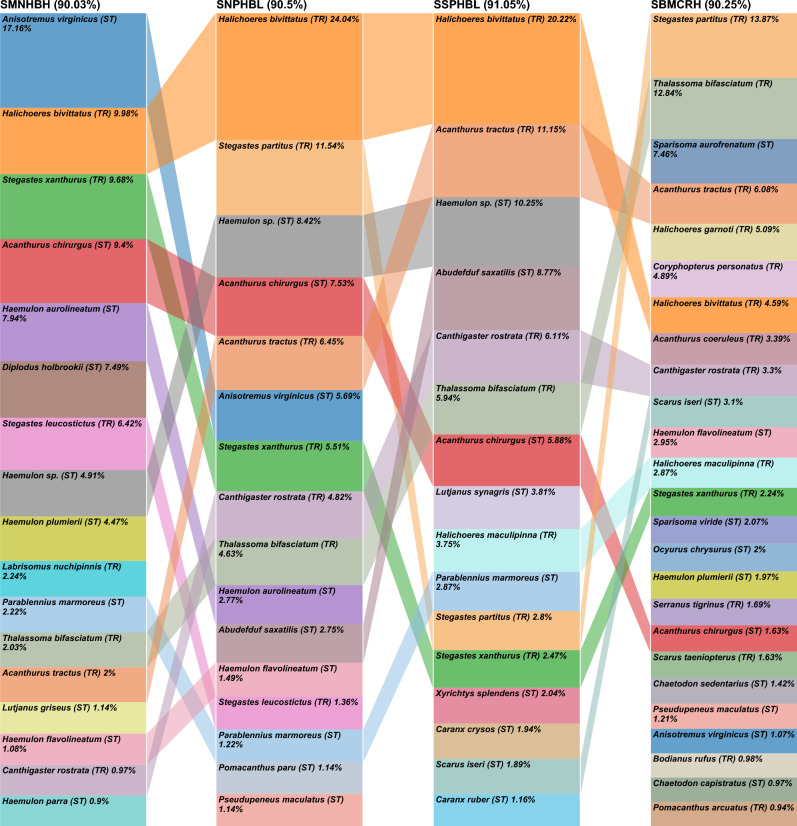
Comparison chart of the species contributing to > 90% of the shallow fish assemblage group similarities for one assemblage per region. Box sizes are relative to the species’ contribution within a group. Colors track across the chart by species illustrating the differences in their relative contribution across the assemblages. Assemblages are arranged from north (left) to south (right). Strata were abbreviated following the sequence depth-coral reef ecoregion-habitat type-relief. Depth: deep (D), shallow (S); Coral reef ecoregion: Broward-Miami (BM), Deerfield (DF), South Palm Beach (SP), North Palm Beach (NP), and Martin (MN); Habitat type: Hardbottom (HB), Coral Reef (CR); Relief: High (H), Low (L). TR = tropical and ST = subtropical.

The species contributing to the similarities within the shallow assemblages differed between regions (Fig. 4). Fifty-one species contributed to 99% of the similarity in the Broward-Miami coral reef high relief (SBMCRH) versus 41 species in the Martin hardbottom high relief (SMNHBH), with 22 in common. Sixteen of the 29 fish (55.2%) in Broward-Miami uncommon between the groups are categorized as tropical, compared to 5 of 19 (26.3%) in Martin. In SBMCRH, 25 tropical species contributed to 68.7% of the group similarity, including *Stegastes partitus, Thalassoma bifasciatum, Acanthurus tractus, Halichoeres garnoti,* and *Coryphopterus personatus* which contributed to 42.8%. In SMNHBH, 27 subtropical species contributed to 63.2% of the group similarity, including *Anisotremus virginicus*, *Acanthurus chirurgus*, *Haemulon aurolineatum*, and *Diplodus holbrooki* which contributed to 42%. *Stegastes partitus* exemplified the northward reduction of tropical species in shallow assemblages, with 98.7% occurrence in SBMCRH and < 17% in Martin further north (Suppl. S6). *Diplodus holbrookii* exemplified the northward increase in shallow assemblage subtropical species, with 81.3% occurrence in SMNHBH versus 11.4% occurrence in SBMHBH.

**Figure 5:**
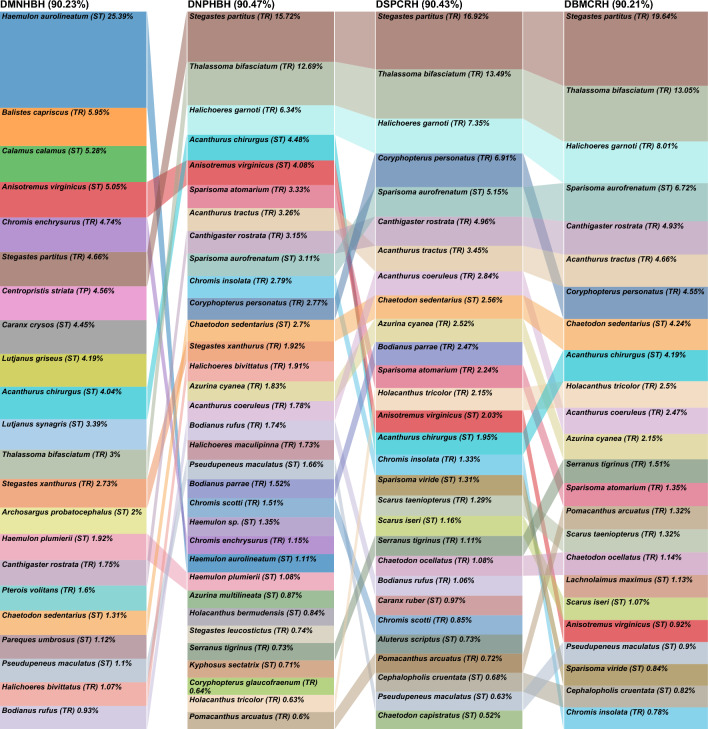
Comparison chart of the species contributing to > 90% of the deep fish assemblage group similarities for one assemblage per region. Box sizes are relative to the species’ contribution within a group. Colors track across the chart by species illustrating the differences in their relative contribution across the assemblages. Assemblages are arranged from north (left) to south (right). Strata were abbreviated following the sequence depth-coral reef ecoregion-habitat type-relief. Depth: deep (D), shallow (S); Coral reef ecoregion: Broward-Miami (BM), Deerfield (DF), South Palm Beach (SP), North Palm Beach (NP), and Martin (MN); Habitat type: Hardbottom (HB), Coral Reef (CR); Relief: High (H), Low (L). TR = tropical and ST = subtropical.

**Figure 6:**
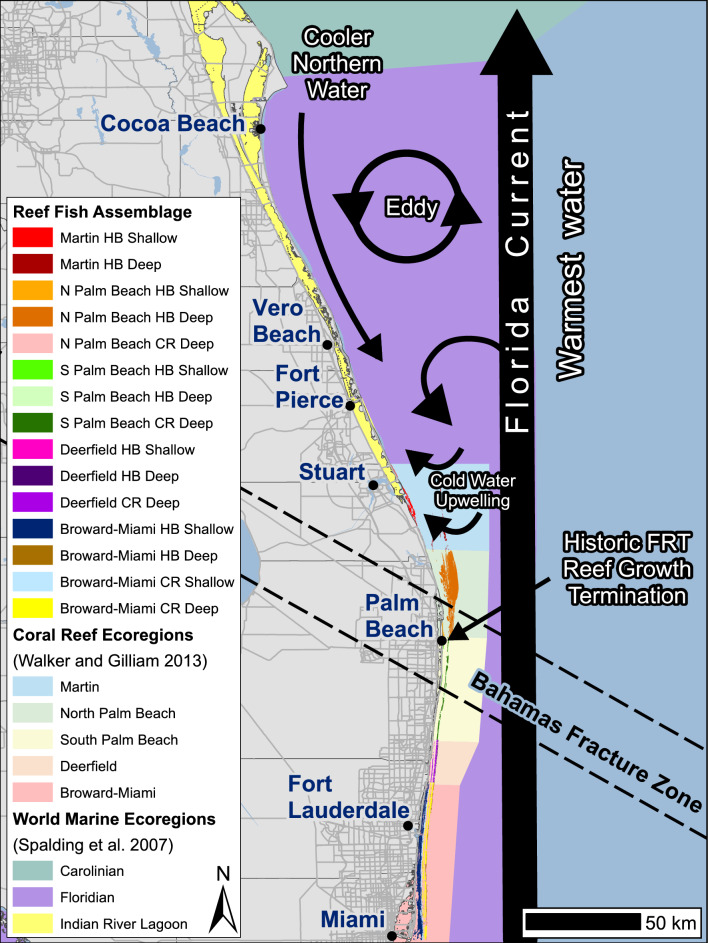
Illustration depicting the reef fish assemblage regions in relation to the hydrodynamics along the southeast Florida coast. A combination of the Florida Current ushering the warmest water offshore, frequent cold-water upwelling, and relatively cooler coastal waters off north of Palm Beach County coincides with the present-day tropical to temperate transition in coastal marine communities and may inhibit the future topicalization of northern locales.

The species contributing to the similarities within the deep assemblages differed between regions (Fig. 5). Fifty-three species contributed to 99% of the similarity in the Broward-Miami coral reef high relief (DBMCRH) versus 50 species in the Martin hardbottom high relief (DMNHBH), with 24 in common. Seventeen of the 29 fish (58.6%) in Broward-Miami uncommon between the groups are categorized as tropical, compared to 4 of 26 (15.4%) in Martin. In DBMCRH, 32 tropical species contributed to 75.3% of the group similarity, including *S. partitus, T. bifasciatum*, and *H. garnoti* which contributed to 40.7%. In SMNHBH, 30 subtropical and one temperate species contributed to 69.7% of the group similarity, including *H. aurolineatum*, *Calamus calamus*, *A. virginicus*, and *Caranx crysos* which contributed to 40.2%. *Halichoeres garnoti* exemplified the northward reduction of tropical species in deep assemblages, with > 88.4% occurrence in all coral reef assemblages and < 18.5% in Martin. *Haemulon aurolineatum* exemplified the northward increase in subtropical species, with 77.8% occurrence in SMNHBH and > 14% occurrence in the Broward-Miami ecoregion (Fig. 6).

## Discussion

Our results support that the density and occurrence of most tropical coral reef fish species drastically decrease along the southeastern Florida coast with a concomitant increase in subtropical fishes and the assemblages where this transition occurs have the highest mean richness in the region (Suppl. S5). These changes spatially coincide with the Bahamas Fracture Zone (BFZ)^65,71^, a geological feature that coincides with the end of historical coral reef growth and where the Florida Current diverges from the coast^68^ (Fig. 6). This divergence carries warm tropical waters into the Gulf Stream, and boundary eddies form, causing frequent episodes of cold-water upwelling^63,70,71,86^. This result is supported by other studies along the east coast of central and northern Florida^32^, where subtropical and tropical reef fish ranges overlap^44,45,87^. For instance, Indian River lagoon reef fish are comprised of a Caribbean assemblage advected northward by the Florida Current and a Carolinian assemblage that migrated on southbound countercurrents and other inshore water mass movements^88^. The ecotone transition is also evident in the coral reef ecosystem along the nFRT, where the amount and extent of distinct benthic habitats attenuate northward^40^ and the benthic macroalgal^89^ and coral assemblages vary with latitude^65,90–93^.

**Figure 7:**
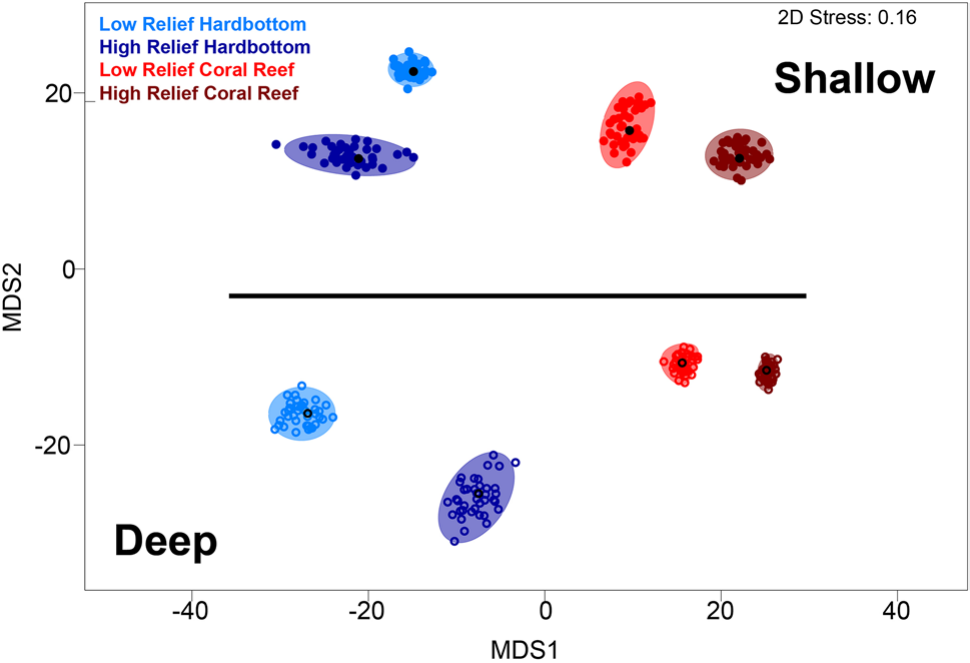
Bootstrap means plot of assemblage density data by habitat depth, habitat type, and relief, illustrating that the absence of region still elicits assemblage distinctions but does not distinguish them along a spatial gradient. Shallow sites are denoted by solid symbols, and deep sites are hollow. Red represents reef habitats, and blue represents hardbottom habitats. Light colors are low relief, and dark colors are high relief.

Other middle- and high-latitude studies have shown similar patterns of increased numbers of warm-water species and decreased numbers of cold-water species^94–96^. With the frequency of warm winters increasing, the possibility grows for typically tropical populations of certain reef fish species to become established in subtropical locations year-round^97^. Some tropical fish species are currently overwintering and possibly spawning in temperate reefs of western Japan, as benthic communities have changed to accommodate them^8^.

Indications of a latitudinal transition were evident in assemblage richness, density, and species composition. Richness was significantly lower in many Martin and North Palm Beach assemblages than in comparable assemblages in southern regions by approximately 5–10 species. The southern assemblages had a higher occurrence and density of tropical species, and the northern assemblages had more subtropical species. The ranges of many of the subtropical species contributing to the regional assemblage differences at the northernmost sites extend much farther north, whereas the ranges of many of the tropical species diminish to the north, indicating that they are less tolerant of colder conditions^83^. In Martin County, two dominant species, *H. aurolineatum* (Tomtate Grunt) and *D. holbrookii* (Spottail Pinfish), are found from 43° N to 33° S and 40° N to 20° N, respectively, whereas two of the species with higher densities of Broward-Miami assemblage regions, *T. bifasciatum* (Bluehead Wrasse) and *S. aurofrenatum* (Redband Parrotfish), are only found from 33° N to 8° N and 32° N to 7° N, respectively^98^. One species, *Centropristis striata* (Black Seabass), was observed 47 times in the Deep Martin assemblages combined and only three times in regions further south. The Black Seabass is described as a temperate fish with a range from Maine to the Gulf of Mexico^99^. Although they have been rarely documented in south Florida during cold winters^98^, they are typically limited to water temperatures lower than 28 °C^100^ and are not a component of coastal tropical reef fish assemblages < 30 m where water temperatures rarely drop below 20 °C^36,91^.

As in many previous studies^1,87,101–104^, depth was one of the most influential factors affecting reef fish distributions. This was evident in the bootstrap means plot where shallow habitat assemblages (< 10 m) were separated far from deep habitat assemblages (≥10 to 33 m). Topography was also significant in defining the assemblage regions but to a lesser extent than depth and geographic location, as shown by the proximity of high- and low-relief assemblages in the bootstrap plot. This agrees with previous research in southeast Florida that found weak but significant reef fish assemblage correlations to topographic complexity while controlling for depth and habitat^54^. The causes for this are likely complex, as there are many factors influencing the total assemblage composition, and may change depending on the relative abundance of more rugosity-dependent fish.

The assemblage structures were spatially variable along the coast. This makes sense since reef fish assemblage composition is strongly related to habitat type^101,105,106^, and the habitat types differ between ecoregions^40^. In the Broward-Miami ecoregion, the Linear Reef-Inner is the primary shallow coral reef habitat, which runs along the entire nearshore shelf, terminating at the Hillsboro inlet^107^. This region is the only shallow region to contain extant coral reef geology. The shallow habitats north of the Hillsboro inlet are antecedent topographies formed by processes other than historical organic reef growth (i.e., exposed rock outcrops)^40,67,107^, leading to the hardbottom classification. However, the fish assemblage differentiation in the deep habitats corresponded to the ecoregions along a continuous reef feature. The Outer Reef is a distinct, relatively continuous, shore-parallel reef that crests at approximately 16 m depth spanning the entire southeast Florida region^40^. Despite the lack of clear geographic breaks in this feature, the relative assemblages were distinct between ecoregions, indicating that factors related to differences in latitude are affecting the reef fish distributions.

Sea surface temperatures are expected to rise over the next century^22,23,108–110^; thus, a holistic approach is needed to monitor reef fish, coral communities, and other tropical species distributions that interconnect with coral reef ecosystems, such as mangroves that serve as nurseries, to track tropicalization^94^. The outcomes of this study provide a statistically derived spatially defined baseline of reef fish assemblages for evaluating the effects of future demographic and structural changes. Tropical species’ poleward range expansion along the nFRT have occurred in the historical past^61^ and have been suggested for contemporary corals^96^, but thus far, contemporary range expansions for fish or corals have not been documented. If warm sea surface temperatures move northward as predicted, opportunities will increase for tropical species to survive in historically subtropical locales, and a poleward shift in the center of biomass of tropical species could occur^21,94,95,111^.

The tropicalization of reef fish in southeast Florida will be evident in multiyear changes in percent occurrence and/or relative species densities between assemblages. The poleward expansion of tropical species is expected to show the homogenization of assemblage regions where adjacent regions become more similar or the regional boundaries expand poleward. 108 species were exclusive to regions south of the BFZ and 35 were exclusive to the north, helping to differentiate the regional assemblages. Greater contributions of the exclusively southern species to the assemblages in more northern ecoregions could indicate tropicalization if the increases persist.

However, climate change effects on reef fish are not linear or predictable^11^, and subtropical environments are proving to be unstable for tropicalization due to extreme cold events periodically affecting their new poleward range limits^112^. The links between poleward expatriation of tropical reef fish and climate warming can be decoupled by localized storm events^11^, opening the possibility of tropical fish being advected far enough north to areas where upwelling is not a factor. Expatriation may also be reliant on fish demographics and behavior. Tropical fish species with large body size, high swimming ability, large size at settlement, and pelagic spawning behavior are more likely to successfully settle into subtropical habitats^13^. And species that can increase their overall behavioral niche breadth while maintaining a moderate to high niche segregation with native temperate species are more likely to colonize temperate locales^27^.

The interaction of Western Boundary Currents and the unique geomorphology of each coastline affects tropical fish ranges differently. Therefore, the study of local systems is important to understanding how fish assemblages respond to global temperature changes because the tropicalization of each coastal system is unique and local controls will ultimately dictate their potential distributions^109^. Although there is no indication of upwelling subsiding in southeast Florida, investigations into the extent of the present and predicted upwelling are warranted to understand the possibility for tropical marine taxa to advect beyond present coastal environmental limitations.

Our results showed that ecoregions, habitat depth, habitat type, and relief are all significant factors in defining assemblage differences. Therefore, appropriate stratification for reef fish surveys and scientific research must incorporate these factors in their designs to statistically determine assemblage differences across the seascape, including those from tropicalization. Measuring tropicalization changes requires an ecoregion classification. Without the ecoregion strata, habitat depth, habitat type, and relief are still significant, but there is no regional spatial context (Fig. 7). Furthermore, effective place-based management strategies need to be informed by this spatial context. Tens of millions of dollars have been spent creating benthic habitat maps of coral reefs throughout the United States and its territories to inventory reef inhabitants, establish coral and reef fish monitoring programs to acquire baselines, and measure temporal changes^113^. An absence of ecoregion classification may be impeding the ability to measure temporal changes as they relate to tropicalization and other factors.

### Supplementary Information

Supplementary Information 1.

Supplementary Information 2.

Supplementary Information 3.

Supplementary Information 4.

Supplementary Information 5.

Supplementary Information 6.

Supplementary Information 7.

Supplementary Information 8.

Supplementary Information 9.

Supplementary Information 10.

## Data Availability

The datasets used and analyzed during the current study are available in the National Oceanic and Atmospheric Administration repository, OneStop: https://data.noaa.gov/onestop/.
