# Supplementary material for: Regional reef fish assemblage maps provide baseline biogeography for tropicalization monitoring
Source: Sci Rep. 2024 Apr 3;14:7893. doi: 10.1038/s41598-024-58185-6 (PMC10991435; doi:10.1038/s41598-024-58185-6)
Supplement: Supplementary file 1 — Supplementary Information 1. [file 41598_2024_58185_MOESM1_ESM.pdf]

S1. p-values for all PERMANOVA pairwise comparisons within each factor.

**Ecoregion comparison**	Habitat Depth	Topographic Relief Low	Topographic Relief High	Habitat Type Hardbottom	Habitat Type Coral Reef
Martin,	Deep	**0.001**	**0.001**	**0.001**	NA
North Palm Beach	Shallow	**0.001**	**0.002**	**0.001**	NA
Martin,	Deep	**0.001**	**0.001**	**0.001**	NA
South Palm Beach	Shallow	**0.001**	**0.001**	**0.001**	NA
Martin,	Deep	**0.001**	**0.001**	**0.001**	NA
Deerfield	Shallow	**0.001**	**0.018**	**0.001**	NA
Martin,	Deep	**0.001**	**0.001**	**0.001**	NA
Broward-Miami	Shallow	**0.001**	**0.001**	**0.001**	NA
North Palm Beach,	Deep	**0.001**	**0.046**	**0.001**	**0.001**
South Palm Beach	Shallow	**0.001**	**0.052**	**0.029**	NA
North Palm Beach,	Deep	**0.001**	**0.001**	**0.006**	**0.001**
Deerfield	Shallow	**0.001**	0.305	**0.001**	NA
North Palm Beach,	Deep	**0.001**	**0.001**	**0.001**	**0.001**
Broward-Miami	Shallow	**0.001**	**0.006**	**0.001**	NA
Deerfield,	Deep	**0.001**	**0.001**	**0.004**	**0.001**
South Palm Beach	Shallow	**0.001**	0.130	**0.001**	0.579
Broward-Miami,	Deep	**0.001**	**0.001**	**0.001**	**0.001**
South Palm Beach	Shallow	**0.001**	**0.001**	**0.001**	**0.003**
Broward-Miami,	Deep	**0.001**	**0.001**	**0.056**	**0.001**
Deerfield	Shallow	**0.001**	**0.028**	**0.001**	0.192

	**Relief**		**Habitat type**	
**Shallow vs Deep**	Low Relief	High Relief	Hardbottom	Coral Reef
Martin	**0.001**	**0.001**	**0.001**	NA
North Palm Beach	**0.001**	0.152	**0.001**	NA
South Palm Beach	**0.001**	**0.001**	**0.001**	**0.009**
Deerfield	**0.001**	**0.016**	**0.001**	0.156
Broward-Miami	**0.001**	**0.001**	**0.001**	**0.001**

**Low vs High Relief**	Deep	Shallow
Martin	**0.001**	**0.001**
North Palm Beach	**0.001**	**0.008**
South Palm Beach	**0.001**	**0.005**
Deerfield	**0.001**	0.659
Broward-Miami	**0.001**	**0.001**

**Hardbottom vs Coral Reef**	Deep	Shallow
Martin	NA	NA
North Palm Beach	**0.042**	NA
South Palm Beach	**0.001**	**0.004**
Deerfield	0.113	0.837
Broward-Miami	**0.002**	**0.001**
