# Supplementary material for: Regional reef fish assemblage maps provide baseline biogeography for tropicalization monitoring
Source: Sci Rep. 2024 Apr 3;14:7893. doi: 10.1038/s41598-024-58185-6 (PMC10991435; doi:10.1038/s41598-024-58185-6)

S2 Density and Richness between relief by ecoregion, depth, type

Where(:Depth == "Deep" & :Ecoregion == "Broward-Miami" & :Type == "Hardbottom")

Fit Group

Oneway Analysis of TotalDensity By Relief

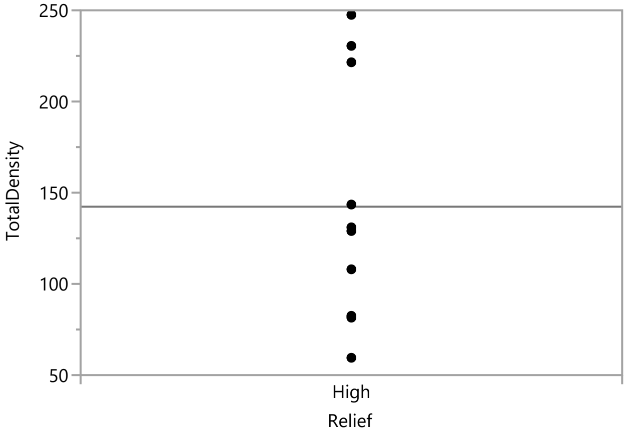

Oneway Analysis of Richness By Relief

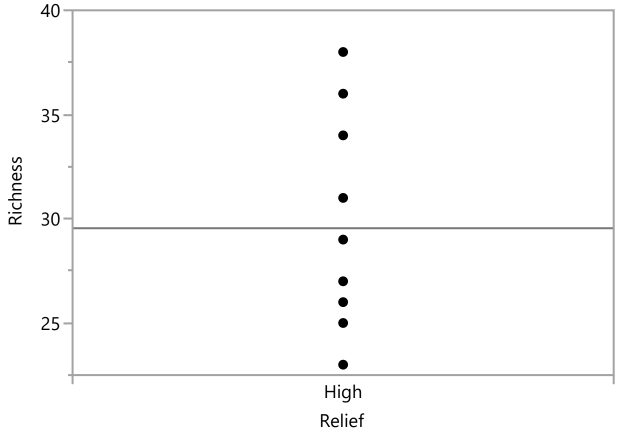

Where(:Depth == "Deep" & :Ecoregion == "Broward-Miami" & :Type == "Reef")

Fit Group

Oneway Analysis of TotalDensity By Relief

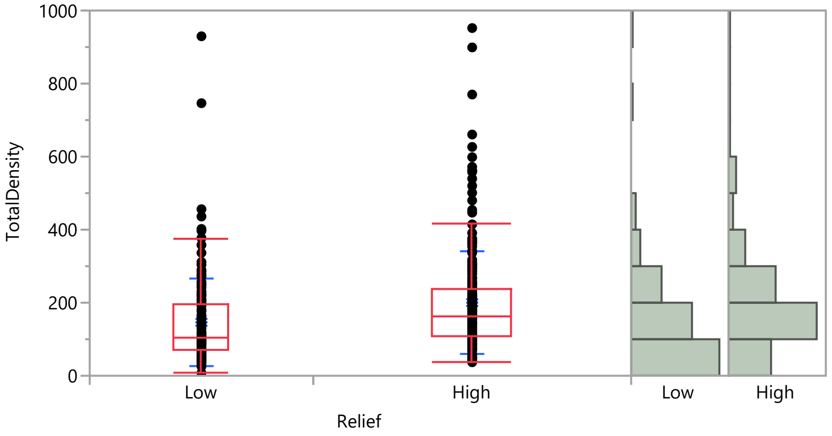

Means and Std Deviations

Level	Number	Mean	Std Dev	Std Err Mean	Lower 95%	Upper 95%
Low	168	146.2691	119.83698	9.2456239	128.01573	164.52247
High	239	200.25131	140.5426	9.0909452	182.34232	218.16031

Wilcoxon / Kruskal-Wallis Tests (Rank Sums)

Level	Count	Score Sum	Expected Score	Score Mean	(Mean-Mean0)/Std0
Low	168	27889.5	34272.0	166.009	-5.462
High	239	55138.5	48756.0	230.705	5.462

2-Sample Test, Normal Approximation

S	Z	Prob>|Z|
27889.5	-5.46220	<.0001*

1-Way Test, ChiSquare Approximation

ChiSquare	DF	Prob>ChiSq
29.8403	1	<.0001*

S2 Density and Richness between relief by ecoregion, depth, type

Fit Group

Oneway Analysis of Richness By Relief

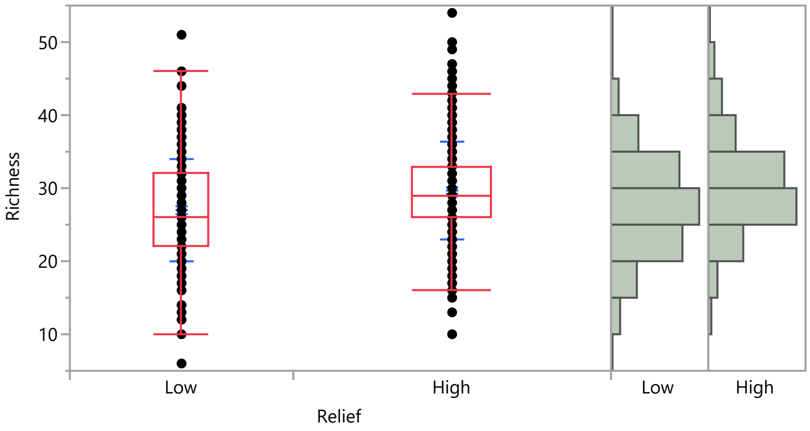

Means and Std Deviations

Level	Number	Mean	Std Dev	Std Err Mean	Lower 95%	Upper 95%
Low	168	26.982143	6.9999771	0.54006	25.915918	28.048368
High	239	29.669456	6.6921775	0.432881	28.816689	30.522224

Wilcoxon / Kruskal-Wallis Tests (Rank Sums)

Level	Count	Score Sum	Expected Score	Score Mean	(Mean-Mean0)/Std0
Low	168	29747.5	34272.0	177.068	-3.877
High	239	53280.5	48756.0	222.931	3.877

2-Sample Test, Normal Approximation

S	Z	Prob>|Z|
29747.5	-3.87678	0.0001*

1-Way Test, ChiSquare Approximation

ChiSquare	DF	Prob>ChiSq
15.0328	1	0.0001*

Where(:Depth == "Deep" & :Ecoregion == "Deerfield" & :Type == "Reef")

Fit Group

S2 Density and Richness between relief by ecoregion, depth, type

Fit Group

Oneway Analysis of TotalDensity By Relief

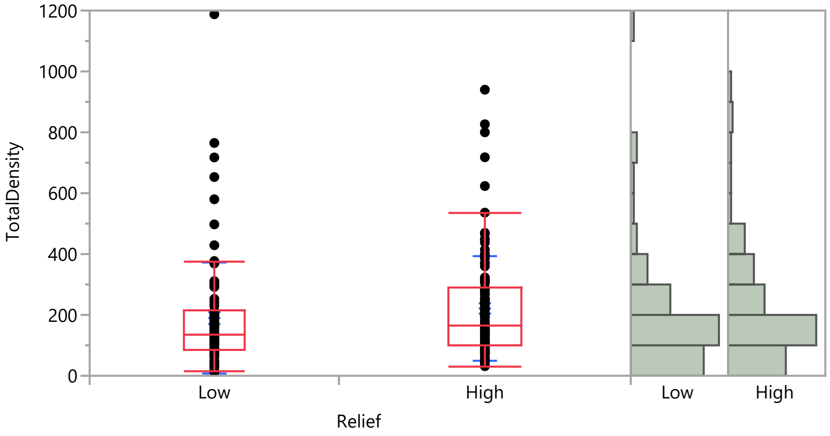

Means and Std Deviations

Level	Number	Mean	Std Dev	Std Err Mean	Lower 95%	Upper 95%
Low	88	189.78843	182.42862	19.446957	151.1355	228.44136
High	103	221.2233	171.91067	16.938862	187.62515	254.82145

Wilcoxon / Kruskal-Wallis Tests (Rank Sums)

Level	Count	Score Sum	Expected Score	Score Mean	(Mean-Mean0)/Std0
Low	88	7697.50	8448.00	87.472	-1.969
High	103	10638.5	9888.00	103.286	1.969

2-Sample Test, Normal Approximation

S	Z	Prob>|Z|
7697.5	-1.96945	0.0489*

1-Way Test, ChiSquare Approximation

ChiSquare	DF	Prob>ChiSq
3.8839	1	0.0488*

S2 Density and Richness between relief by ecoregion, depth, type

Fit Group

Oneway Analysis of Richness By Relief

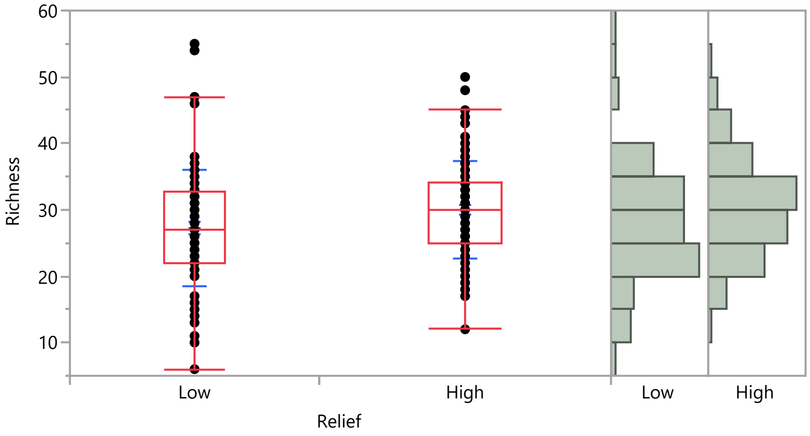

Means and Std Deviations

Level	Number	Mean	Std Dev	Std Err Mean	Lower 95%	Upper 95%
Low	88	27.25	8.7641511	0.9342616	25.393054	29.106946
High	103	30	7.3431307	0.7235402	28.564862	31.435138

Wilcoxon / Kruskal-Wallis Tests (Rank Sums)

Level	Count	Score Sum	Expected Score	Score Mean	(Mean-Mean0)/Std0
Low	88	7562.50	8448.00	85.938	-2.326
High	103	10773.5	9888.00	104.597	2.326

2-Sample Test, Normal Approximation

S	Z	Prob>|Z|
7562.5	-2.32633	0.0200*

1-Way Test, ChiSquare Approximation

ChiSquare	DF	Prob>ChiSq
5.4179	1	0.0199*

Where(:Depth == "Deep" & :Ecoregion == "Martin" & :Type == "Hardbottom")

Fit Group

S2 Density and Richness between relief by ecoregion, depth, type

Fit Group

Oneway Analysis of TotalDensity By Relief

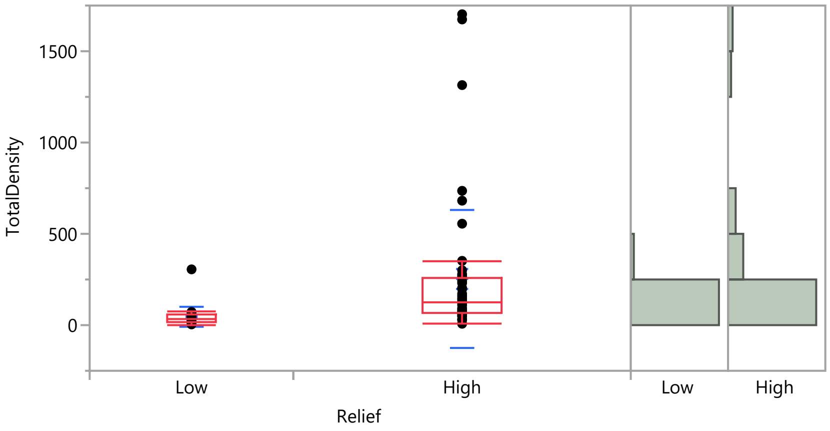

Means and Std Deviations

Level	Number	Mean	Std Dev	Std Err Mean	Lower 95%	Upper 95%
Low	29	45.775862	54.648321	10.147939	24.988752	66.562972
High	48	252.80208	377.84022	54.536538	143.08872	362.51545

Wilcoxon / Kruskal-Wallis Tests (Rank Sums)

Level	Count	Score Sum	Expected Score	Score Mean	(Mean-Mean0)/Std0
Low	29	612.500	1131.00	21.1207	-5.446
High	48	2390.50	1872.00	49.8021	5.446

2-Sample Test, Normal Approximation

S	Z	Prob>|Z|
612.5	-5.44580	<.0001*

1-Way Test, ChiSquare Approximation

ChiSquare	DF	Prob>ChiSq
29.7141	1	<.0001*

S2 Density and Richness between relief by ecoregion, depth, type

Fit Group

Oneway Analysis of Richness By Relief

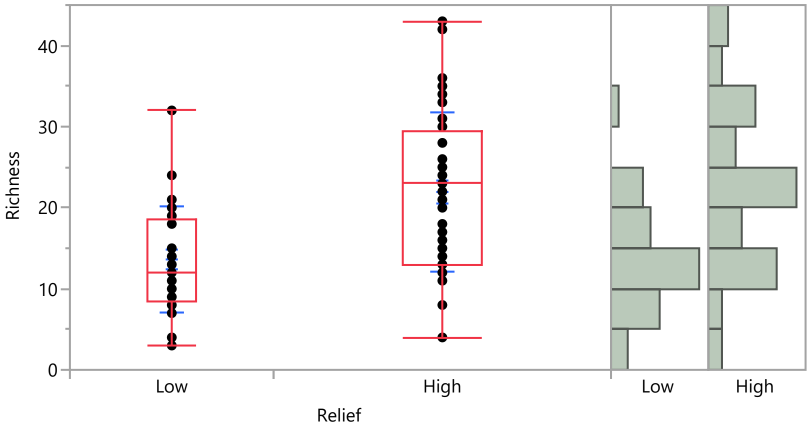

Means and Std Deviations

Level	Number	Mean	Std Dev	Std Err Mean	Lower 95%	Upper 95%
Low	29	13.62069	6.5487937	1.2160805	11.129662	16.111718
High	48	21.9375	9.8248626	1.4180968	19.084657	24.790343

Wilcoxon / Kruskal-Wallis Tests (Rank Sums)

Level	Count	Score Sum	Expected Score	Score Mean	(Mean-Mean0)/Std0
Low	29	763.000	1131.00	26.3103	-3.870
High	48	2240.00	1872.00	46.6667	3.870

2-Sample Test, Normal Approximation

S	Z	Prob>|Z|
763	-3.86958	0.0001*

1-Way Test, ChiSquare Approximation

ChiSquare	DF	Prob>ChiSq
15.0145	1	0.0001*

Where(:Depth == "Deep" & :Ecoregion == "North Palm Beach" & :Type == "Hardbottom")

Fit Group

S2 Density and Richness between relief by ecoregion, depth, type

Fit Group

Oneway Analysis of TotalDensity By Relief

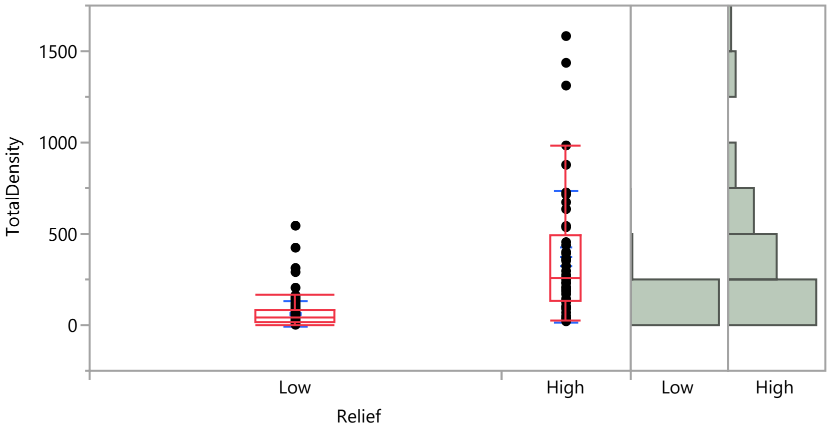

Means and Std Deviations

Level	Number	Mean	Std Dev	Std Err Mean	Lower 95%	Upper 95%
Low	156	61.471154	69.916707	5.5978166	50.413299	72.529009
High	49	373.92347	360.26943	51.467062	270.44201	477.40493

Wilcoxon / Kruskal-Wallis Tests (Rank Sums)

Level	Count	Score Sum	Expected Score	Score Mean	(Mean-Mean0)/Std0
Low	156	13011.0	16068.0	83.404	-8.438
High	49	8104.00	5047.00	165.388	8.438

2-Sample Test, Normal Approximation

S	Z	Prob>|Z|
8104	8.43782	<.0001*

1-Way Test, ChiSquare Approximation

ChiSquare	DF	Prob>ChiSq
71.2202	1	<.0001*

S2 Density and Richness between relief by ecoregion, depth, type

Fit Group

Oneway Analysis of Richness By Relief

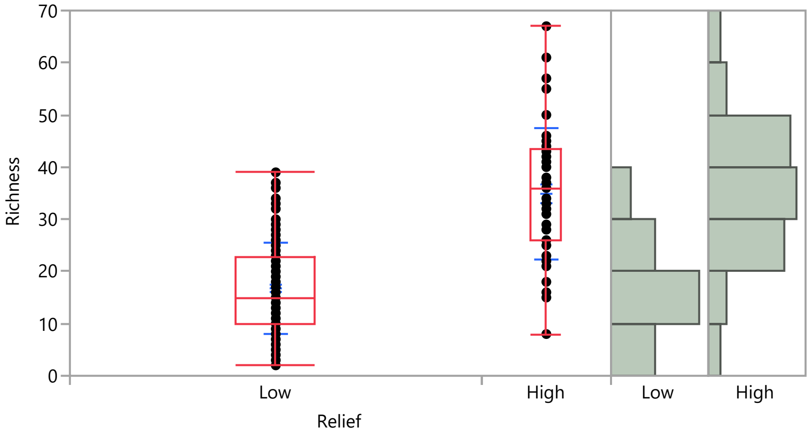

Means and Std Deviations

Level	Number	Mean	Std Dev	Std Err Mean	Lower 95%	Upper 95%
Low	156	16.762821	8.7459574	0.700237	15.379581	18.14606
High	49	34.877551	12.594034	1.7991477	31.260122	38.49498

Wilcoxon / Kruskal-Wallis Tests (Rank Sums)

Level	Count	Score Sum	Expected Score	Score Mean	(Mean-Mean0)/Std0
Low	156	13169.0	16068.0	84.417	-8.006
High	49	7946.00	5047.00	162.163	8.006

2-Sample Test, Normal Approximation

S	Z	Prob>|Z|
7946	8.00572	<.0001*

1-Way Test, ChiSquare Approximation

ChiSquare	DF	Prob>ChiSq
64.1136	1	<.0001*

Where(:Depth == "Deep" & :Ecoregion == "North Palm Beach" &
:Type == "Reef")

Fit Group

Oneway Analysis of TotalDensity By Relief

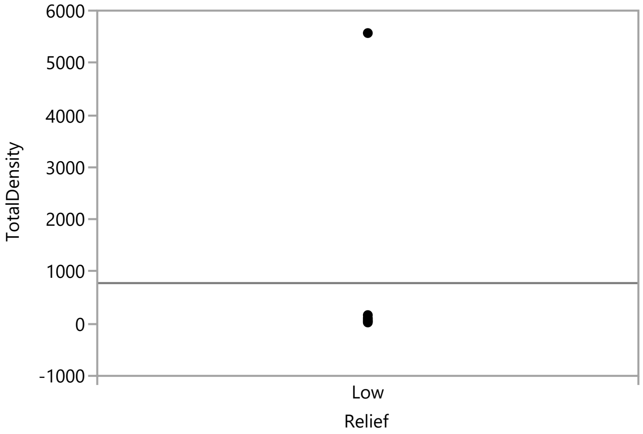

Oneway Analysis of Richness By Relief

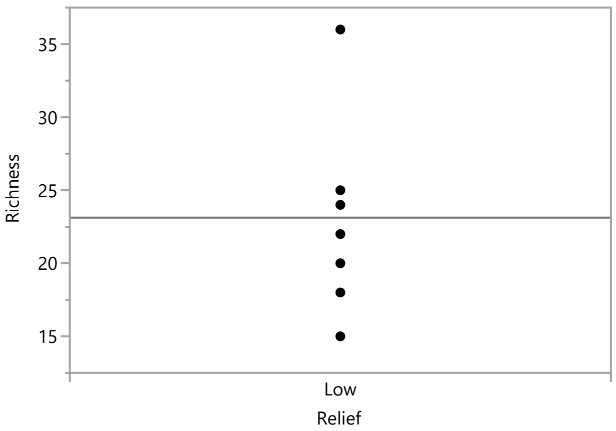

Where(:Depth == "Deep" & :Ecoregion == "South Palm Beach" &
:Type == "Hardbottom")

S2 Density and Richness between relief by ecoregion, depth, type

Fit Group

Oneway Analysis of TotalDensity By Relief

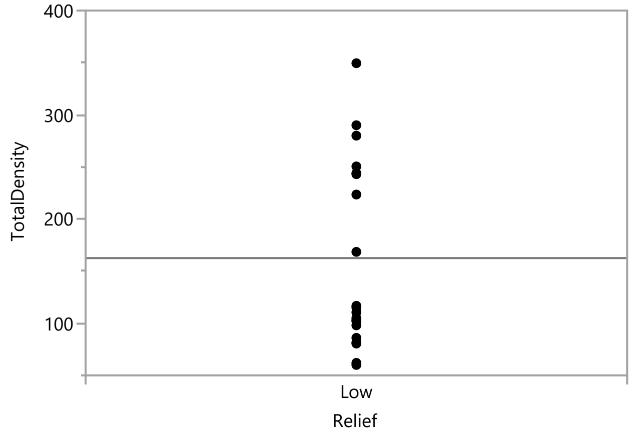

Oneway Analysis of Richness By Relief

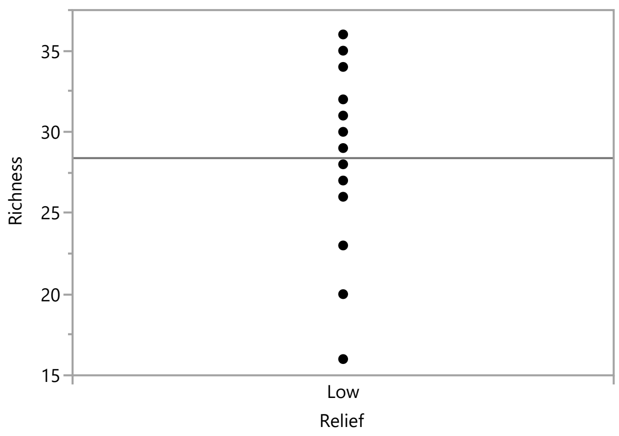

Where(:Depth == "Deep" & :Ecoregion == "South Palm Beach" & :Type == "Reef")

Fit Group

Oneway Analysis of TotalDensity By Relief

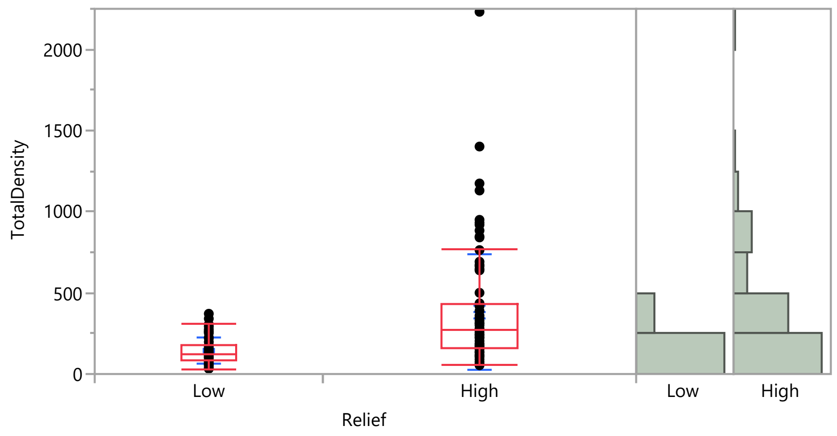

Means and Std Deviations

Level	Number	Mean	Std Dev	Std Err Mean	Lower 95%	Upper 95%
Low	59	144.47458	80.591906	10.492173	123.47221	165.47694
High	81	381.96605	356.02872	39.558747	303.24163	460.69047

Wilcoxon / Kruskal-Wallis Tests (Rank Sums)

Level	Count	Score Sum	Expected Score	Score Mean	(Mean-Mean0)/Std0
Low	59	2740.50	4159.50	46.4492	-5.986
High	81	7129.50	5710.50	88.0185	5.986

2-Sample Test, Normal Approximation

S	Z	Prob>|Z|
2740.5	-5.98614	<.0001*

1-Way Test, ChiSquare Approximation

ChiSquare	DF	Prob>ChiSq
35.8592	1	<.0001*

S2 Density and Richness between relief by ecoregion, depth, type

Fit Group

Oneway Analysis of Richness By Relief

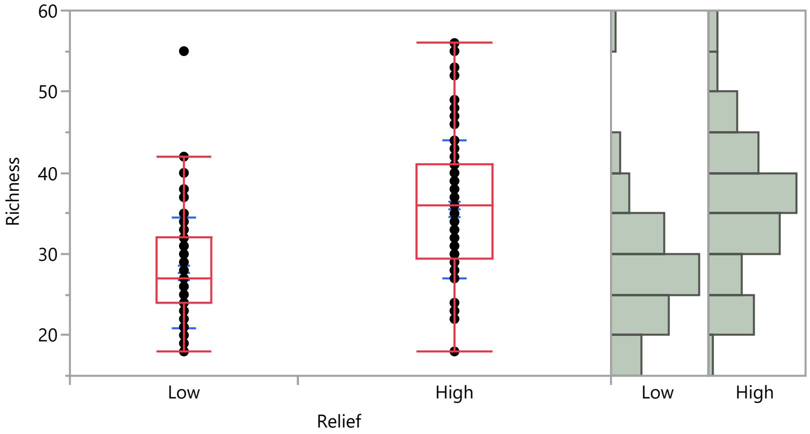

Means and Std Deviations

Level	Number	Mean	Std Dev	Std Err Mean	Lower 95%	Upper 95%
Low	59	27.677966	6.8214688	0.8880796	25.900282	29.455651
High	81	35.506173	8.5075312	0.9452812	33.625003	37.387342

Wilcoxon / Kruskal-Wallis Tests (Rank Sums)

Level	Count	Score Sum	Expected Score	Score Mean	(Mean-Mean0)/Std0
Low	59	2887.00	4159.50	48.9322	-5.373
High	81	6983.00	5710.50	86.2099	5.373

2-Sample Test, Normal Approximation

S	Z	Prob>|Z|
2887	-5.37269	<.0001*

1-Way Test, ChiSquare Approximation

ChiSquare	DF	Prob>ChiSq
28.8885	1	<.0001*

Where(:Depth == "Shallow" & :Ecoregion == "Broward-Miami" &
:Type == "Hardbottom")

Fit Group

S2 Density and Richness between relief by ecoregion, depth, type

Fit Group

Oneway Analysis of TotalDensity By Relief

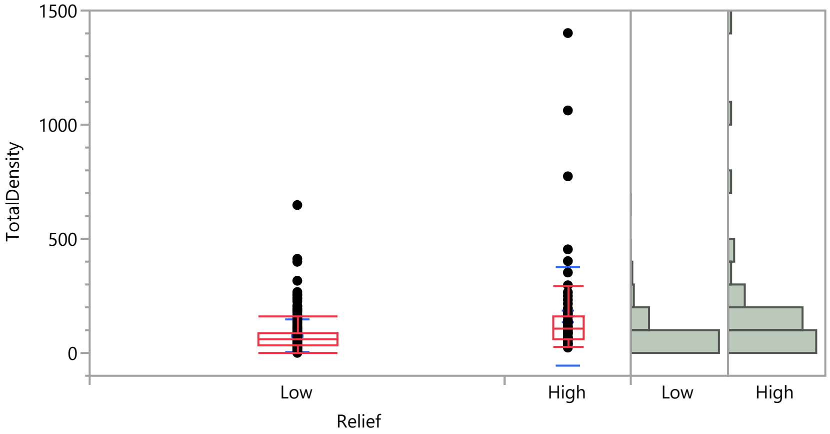

Means and Std Deviations

Level	Number	Mean	Std Dev	Std Err Mean	Lower 95%	Upper 95%
Low	241	75.437178	71.523387	4.6072264	66.361414	84.512943
High	73	160.28082	215.74979	25.251603	109.94267	210.61897

Wilcoxon / Kruskal-Wallis Tests (Rank Sums)

Level	Count	Score Sum	Expected Score	Score Mean	(Mean-Mean0)/Std0
Low	241	34131.0	37957.5	141.622	-5.630
High	73	15324.0	11497.5	209.918	5.630

2-Sample Test, Normal Approximation

S	Z	Prob>|Z|
15324	5.63016	<.0001*

1-Way Test, ChiSquare Approximation

ChiSquare	DF	Prob>ChiSq
31.7070	1	<.0001*

S2 Density and Richness between relief by ecoregion, depth, type

Fit Group

Oneway Analysis of Richness By Relief

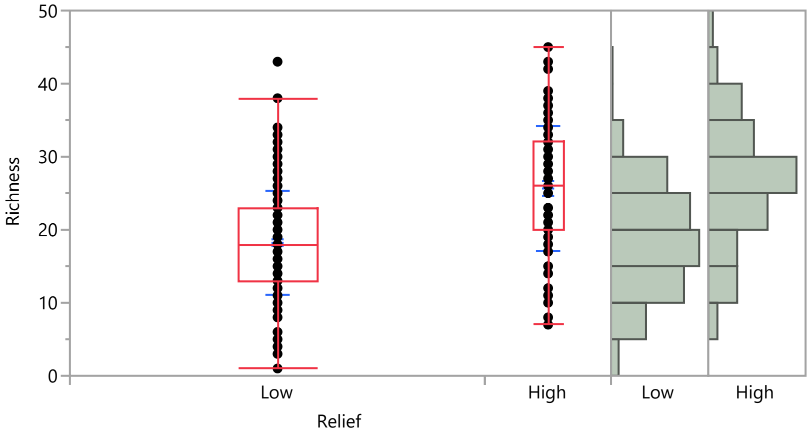

Means and Std Deviations

Level	Number	Mean	Std Dev	Std Err Mean	Lower 95%	Upper 95%
Low	241	18.215768	7.1229617	0.4588303	17.311919	19.119616
High	73	25.643836	8.5332192	0.9987378	23.652888	27.634783

Wilcoxon / Kruskal-Wallis Tests (Rank Sums)

Level	Count	Score Sum	Expected Score	Score Mean	(Mean-Mean0)/Std0
Low	241	33606.0	37957.5	139.444	-6.408
High	73	15849.0	11497.5	217.110	6.408

2-Sample Test, Normal Approximation

S	Z	Prob>|Z|
15849	6.40791	<.0001*

1-Way Test, ChiSquare Approximation

ChiSquare	DF	Prob>ChiSq
41.0707	1	<.0001*

Where(:Depth == "Shallow" & :Ecoregion == "Broward-Miami" &
:Type == "Reef")

Fit Group

S2 Density and Richness between relief by ecoregion, depth, type

Fit Group

Oneway Analysis of TotalDensity By Relief

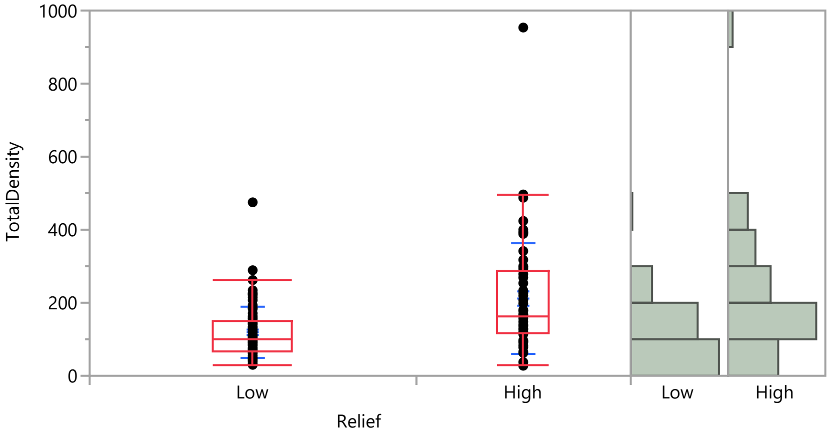

Means and Std Deviations

Level	Number	Mean	Std Dev	Std Err Mean	Lower 95%	Upper 95%
Low	91	119.08086	70.017025	7.3397786	104.49911	133.66261
High	60	211.41875	151.4655	19.554112	172.29106	250.54644

Wilcoxon / Kruskal-Wallis Tests (Rank Sums)

Level	Count	Score Sum	Expected Score	Score Mean	(Mean-Mean0)/Std0
Low	91	5688.00	6916.00	62.5055	-4.668
High	60	5788.00	4560.00	96.4667	4.668

2-Sample Test, Normal Approximation

S	Z	Prob>|Z|
5788	4.66768	<.0001*

1-Way Test, ChiSquare Approximation

ChiSquare	DF	Prob>ChiSq
21.8050	1	<.0001*

S2 Density and Richness between relief by ecoregion, depth, type

Fit Group

Oneway Analysis of Richness By Relief

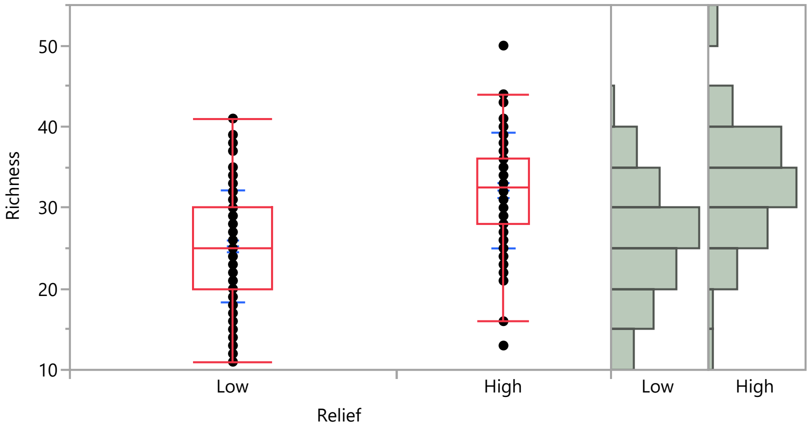

Means and Std Deviations

Level	Number	Mean	Std Dev	Std Err Mean	Lower 95%	Upper 95%
Low	91	25.230769	6.9074467	0.7240972	23.792224	26.669315
High	60	32.116667	7.1357103	0.9212162	30.273317	33.960016

Wilcoxon / Kruskal-Wallis Tests (Rank Sums)

Level	Count	Score Sum	Expected Score	Score Mean	(Mean-Mean0)/Std0
Low	91	5501.00	6916.00	60.4505	-5.383
High	60	5975.00	4560.00	99.5833	5.383

2-Sample Test, Normal Approximation

S	Z	Prob>|Z|
5975	5.38338	<.0001*

1-Way Test, ChiSquare Approximation

ChiSquare	DF	Prob>ChiSq
29.0013	1	<.0001*

Where(:Depth == "Shallow" & :Ecoregion == "Deerfield" & :Type == "Hardbottom")

Fit Group

Oneway Analysis of TotalDensity By Relief

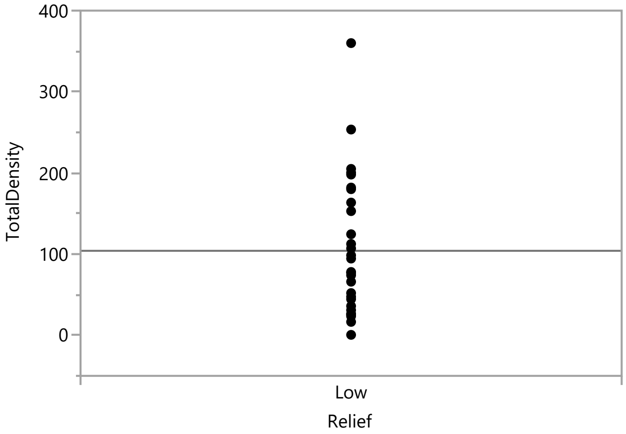

Oneway Analysis of Richness By Relief

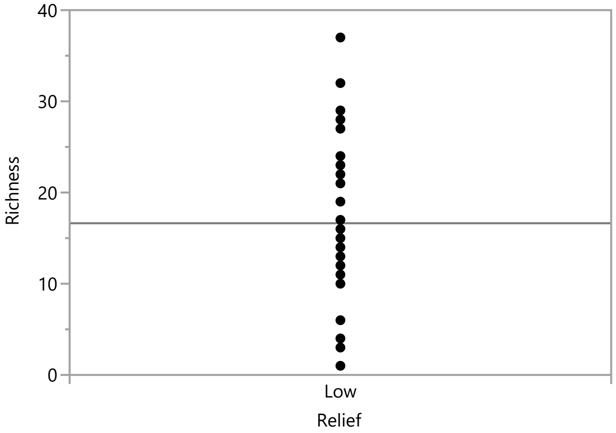

Where(:Depth == "Shallow" & :Ecoregion == "Martin" & :Type == "Hardbottom")

S2 Density and Richness between relief by ecoregion, depth, type

Fit Group

Oneway Analysis of TotalDensity By Relief

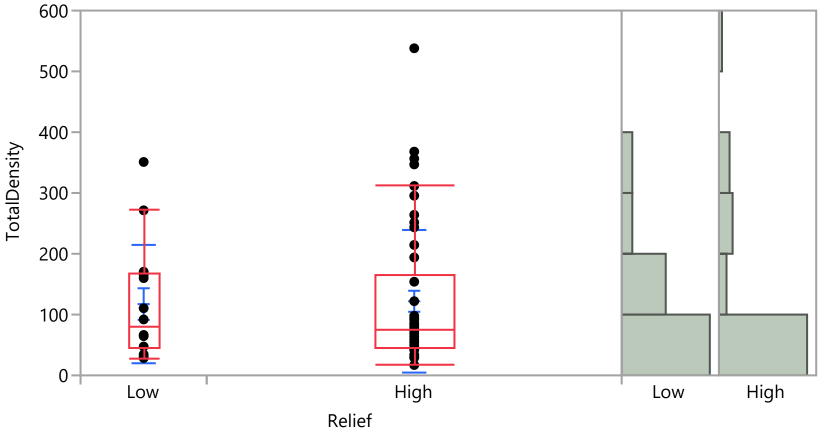

Means and Std Deviations

Level	Number	Mean	Std Dev	Std Err Mean	Lower 95%	Upper 95%
Low	14	117.25	97.338179	26.014723	61.048608	173.45139
High	46	121.88043	117.29645	17.294418	87.047688	156.71318

Wilcoxon / Kruskal-Wallis Tests (Rank Sums)

Level	Count	Score Sum	Expected Score	Score Mean	(Mean-Mean0)/Std0
Low	14	430.000	427.000	30.7143	0.044
High	46	1400.00	1403.00	30.4348	-0.044

2-Sample Test, Normal Approximation

S	Z	Prob>|Z|
430	0.04370	0.9651

1-Way Test, ChiSquare Approximation

ChiSquare	DF	Prob>ChiSq
0.0027	1	0.9582

S2 Density and Richness between relief by ecoregion, depth, type

Fit Group

Oneway Analysis of Richness By Relief

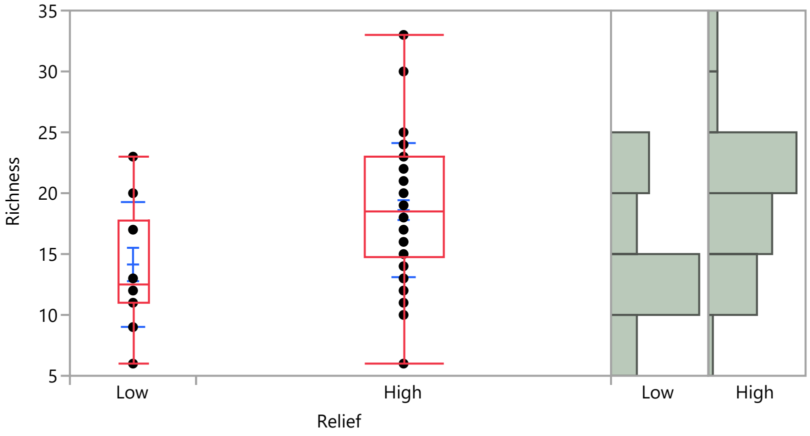

Means and Std Deviations

Level	Number	Mean	Std Dev	Std Err Mean	Lower 95%	Upper 95%
Low	14	14.142857	5.1269596	1.3702376	11.182639	17.103075
High	46	18.608696	5.5074828	0.812034	16.973175	20.244216

Wilcoxon / Kruskal-Wallis Tests (Rank Sums)

Level	Count	Score Sum	Expected Score	Score Mean	(Mean-Mean0)/Std0
Low	14	276.500	427.000	19.7500	-2.628
High	46	1553.50	1403.00	33.7717	2.628

2-Sample Test, Normal Approximation

S	Z	Prob>|Z|
276.5	-2.62782	0.0086*

1-Way Test, ChiSquare Approximation

ChiSquare	DF	Prob>ChiSq
6.9516	1	0.0084*

Where(:Depth == "Shallow" & :Ecoregion == "North Palm Beach" &
:Type == "Hardbottom")

Fit Group

Oneway Analysis of TotalDensity By Relief

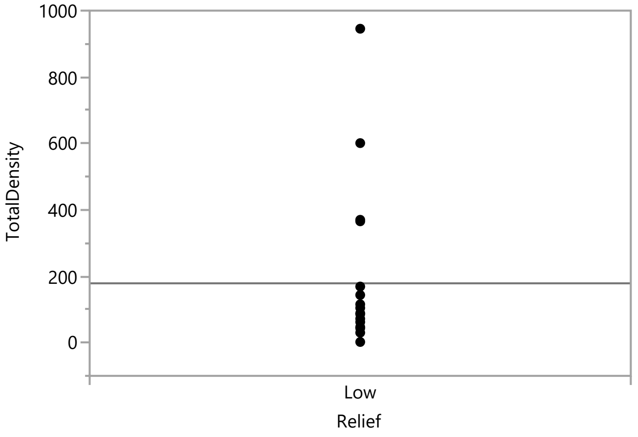

Oneway Analysis of Richness By Relief

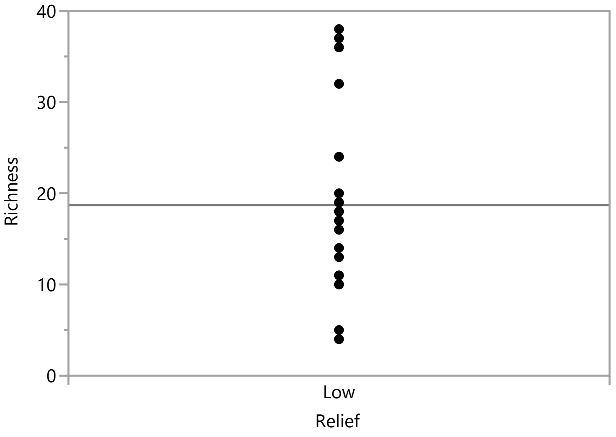

Where(:Depth == "Shallow" & :Ecoregion == "South Palm Beach" &
:Type == "Hardbottom")

S2 Density and Richness between relief by ecoregion, depth, type

Fit Group

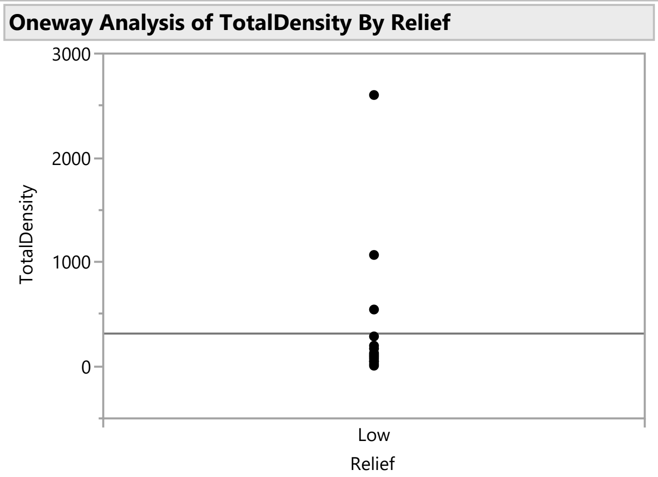

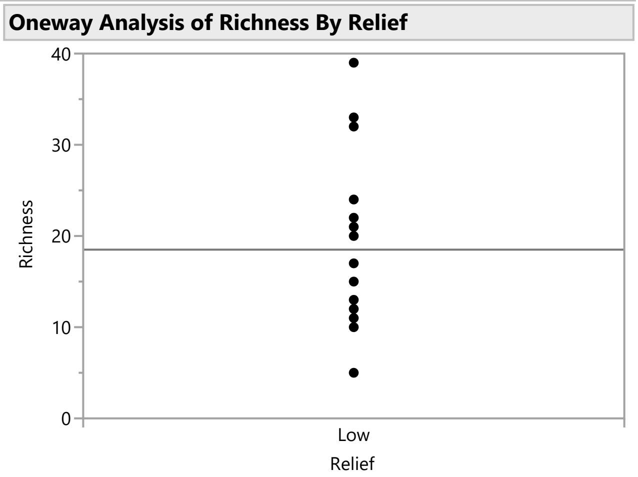

Supplement: Supplementary file 2 — Supplementary Information 2. [file 41598_2024_58185_MOESM2_ESM.pdf]
