# Supplementary material for: Regional reef fish assemblage maps provide baseline biogeography for tropicalization monitoring
Source: Sci Rep. 2024 Apr 3;14:7893. doi: 10.1038/s41598-024-58185-6 (PMC10991435; doi:10.1038/s41598-024-58185-6)
Supplement: Supplementary file 3 — Supplementary Information 3. [file 41598_2024_58185_MOESM3_ESM.pdf]

S3 Density and Richness between depth by ecoregion, type, relief

Where(:Ecoregion == "Broward-Miami" & :Type 2 == "Coral Reef"
& :Relief == "Low")

Fit Group

Oneway Analysis of TotalDensity By Depth

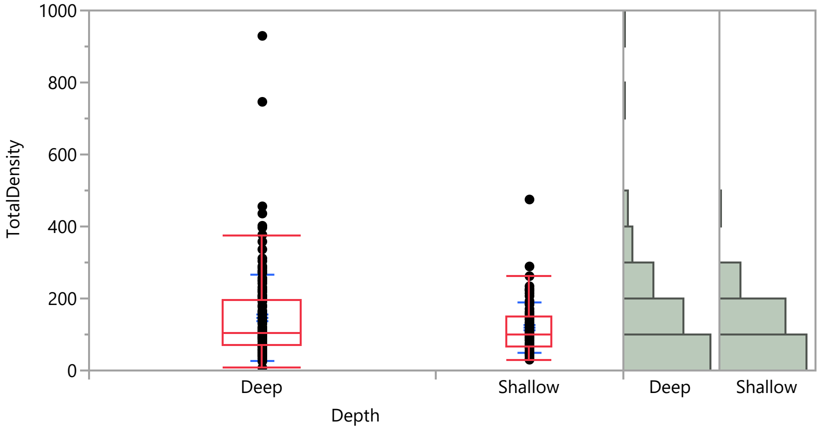

Means and Std Deviations

Level	Number	Mean	Std Dev	Std Err Mean	Lower 95%	Upper 95%
Deep	168	146.2691	119.83698	9.2456239	128.01573	164.52247
Shallow	91	119.08086	70.017025	7.3397786	104.49911	133.66261

Wilcoxon / Kruskal-Wallis Tests (Rank Sums)

Level	Count	Score Sum	Expected Score	Score Mean	(Mean-Mean0)/Std0
Deep	168	22492.5	21840.0	133.884	1.133
Shallow	91	11177.5	11830.0	122.830	-1.133

2-Sample Test, Normal Approximation

S	Z	Prob>|Z|
11177.5	-1.13288	0.2573

1-Way Test, ChiSquare Approximation

ChiSquare	DF	Prob>ChiSq
1.2854	1	0.2569

S3 Density and Richness between depth by ecoregion, type, relief

Fit Group

Oneway Analysis of Richness By Depth

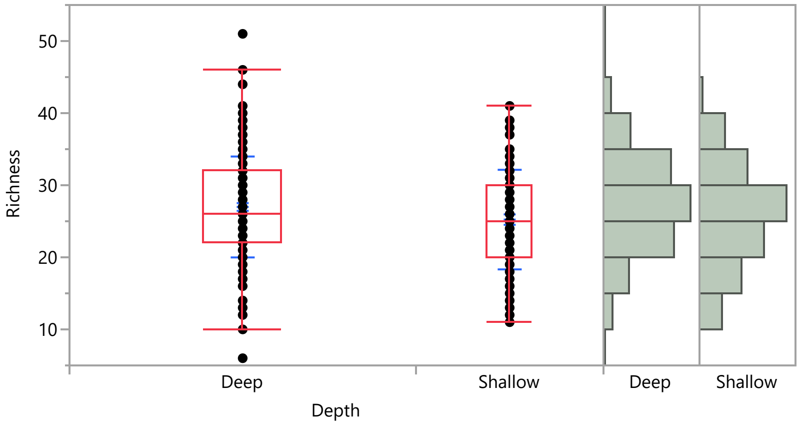

Means and Std Deviations

Level	Number	Mean	Std Dev	Std Err Mean	Lower 95%	Upper 95%
Deep	168	26.982143	6.9999771	0.54006	25.915918	28.048368
Shallow	91	25.230769	6.9074467	0.7240972	23.792224	26.669315

Wilcoxon / Kruskal-Wallis Tests (Rank Sums)

Level	Count	Score Sum	Expected Score	Score Mean	(Mean-Mean0)/Std0
Deep	168	22875.0	21840.0	136.161	1.800
Shallow	91	10795.0	11830.0	118.626	-1.800

2-Sample Test, Normal Approximation

S	Z	Prob>|Z|
10795	-1.79953	0.0719

1-Way Test, ChiSquare Approximation

ChiSquare	DF	Prob>ChiSq
3.2414	1	0.0718

Where(:Ecoregion == "Broward-Miami" & :Type 2 == "Coral Reef"
& :Relief == "High")

Fit Group

S3 Density and Richness between depth by ecoregion, type, relief

Fit Group

Oneway Analysis of TotalDensity By Depth

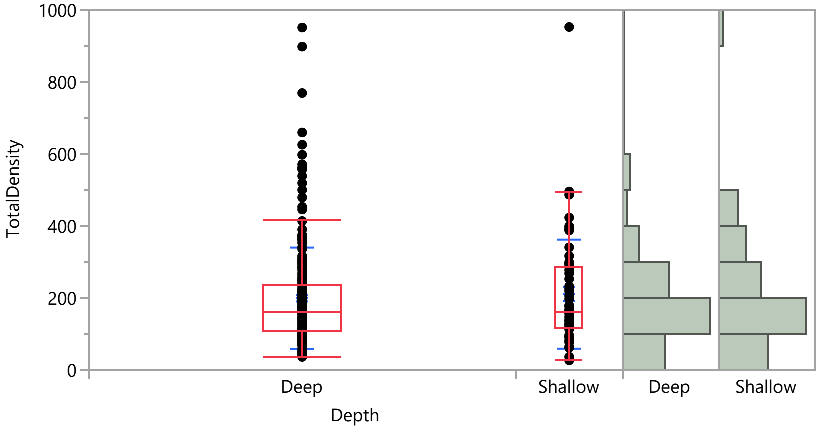

Means and Std Deviations

Level	Number	Mean	Std Dev	Std Err Mean	Lower 95%	Upper 95%
Deep	239	200.25131	140.5426	9.0909452	182.34232	218.16031
Shallow	60	211.41875	151.4655	19.554112	172.29106	250.54644

Wilcoxon / Kruskal-Wallis Tests (Rank Sums)

Level	Count	Score Sum	Expected Score	Score Mean	(Mean-Mean0)/Std0
Deep	239	35511.5	35850.0	148.584	-0.565
Shallow	60	9338.50	9000.00	155.642	0.565

2-Sample Test, Normal Approximation

S	Z	Prob>|Z|
9338.5	0.56452	0.5724

1-Way Test, ChiSquare Approximation

ChiSquare	DF	Prob>ChiSq
0.3196	1	0.5718

S3 Density and Richness between depth by ecoregion, type, relief

Fit Group

Oneway Analysis of Richness By Depth

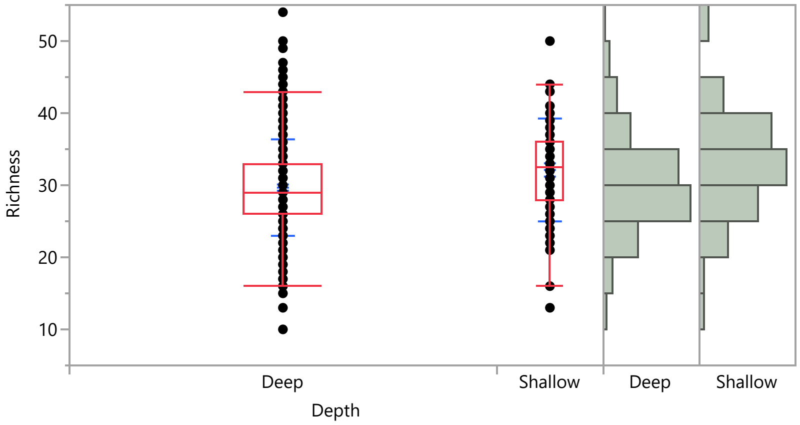

Means and Std Deviations

Level	Number	Mean	Std Dev	Std Err Mean	Lower 95%	Upper 95%
Deep	239	29.669456	6.6921775	0.432881	28.816689	30.522224
Shallow	60	32.116667	7.1357103	0.9212162	30.273317	33.960016

Wilcoxon / Kruskal-Wallis Tests (Rank Sums)

Level	Count	Score Sum	Expected Score	Score Mean	(Mean-Mean0)/Std0
Deep	239	34154.5	35850.0	142.906	-2.835
Shallow	60	10695.5	9000.00	178.258	2.835

2-Sample Test, Normal Approximation

S	Z	Prob>|Z|
10695.5	2.83479	0.0046*

1-Way Test, ChiSquare Approximation

ChiSquare	DF	Prob>ChiSq
8.0408	1	0.0046*

Where(:Ecoregion == "Broward-Miami" & :Type 2 == "Hardbottom"
& :Relief == "Low")

Fit Group

Oneway Analysis of TotalDensity By Depth

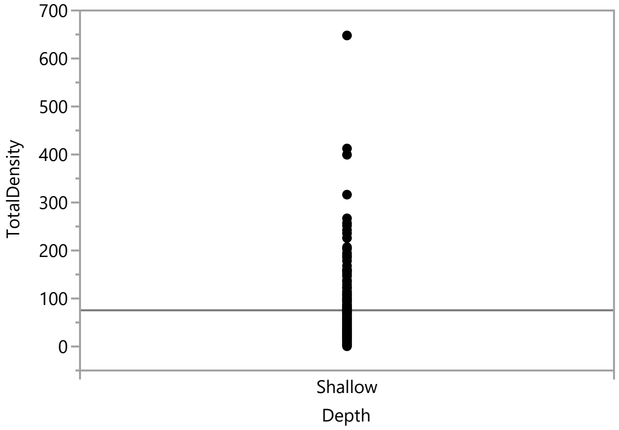

Oneway Analysis of Richness By Depth

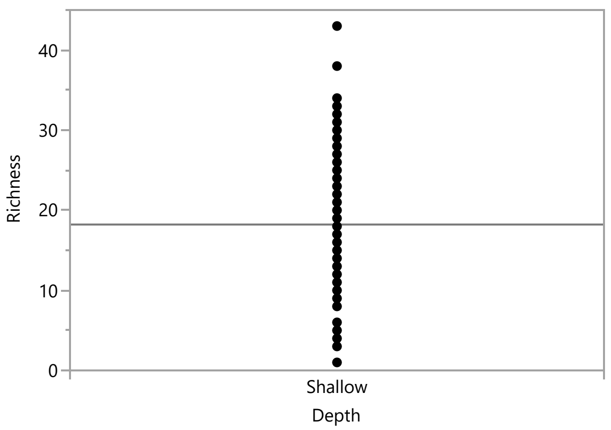

Where(:Ecoregion == "Broward-Miami" & :Type 2 == "Hardbottom"
& :Relief == "High")

S3 Density and Richness between depth by ecoregion, type, relief

Fit Group

Oneway Analysis of TotalDensity By Depth

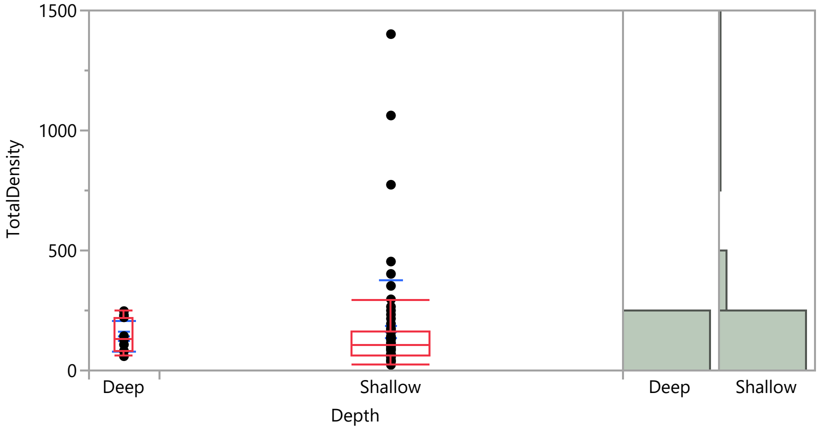

Means and Std Deviations

Level	Number	Mean	Std Dev	Std Err Mean	Lower 95%	Upper 95%
Deep	11	142.31818	63.919978	19.272599	99.376156	185.26021
Shallow	73	160.28082	215.74979	25.251603	109.94267	210.61897

Wilcoxon / Kruskal-Wallis Tests (Rank Sums)

Level	Count	Score Sum	Expected Score	Score Mean	(Mean-Mean0)/Std0
Deep	11	550.500	467.500	50.0455	1.094
Shallow	73	3019.50	3102.50	41.3630	-1.094

2-Sample Test, Normal Approximation

S	Z	Prob>|Z|
550.5	1.09398	0.2740

1-Way Test, ChiSquare Approximation

ChiSquare	DF	Prob>ChiSq
1.2113	1	0.2711

S3 Density and Richness between depth by ecoregion, type, relief

Fit Group

Oneway Analysis of Richness By Depth

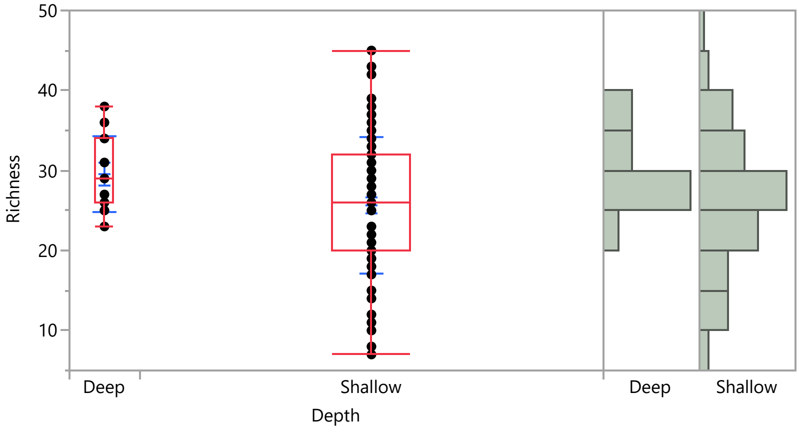

Means and Std Deviations

Level	Number	Mean	Std Dev	Std Err Mean	Lower 95%	Upper 95%
Deep	11	29.545455	4.7405408	1.4293268	26.360716	32.730193
Shallow	73	25.643836	8.5332192	0.9987378	23.652888	27.634783

Wilcoxon / Kruskal-Wallis Tests (Rank Sums)

Level	Count	Score Sum	Expected Score	Score Mean	(Mean-Mean0)/Std0
Deep	11	584.000	467.500	53.0909	1.540
Shallow	73	2986.00	3102.50	40.9041	-1.540

2-Sample Test, Normal Approximation

S	Z	Prob>|Z|
584	1.54025	0.1235

1-Way Test, ChiSquare Approximation

ChiSquare	DF	Prob>ChiSq
2.3929	1	0.1219

Where(:Ecoregion == "Deerfield" & :Type 2 == "Coral Reef" &
:Relief == "Low")

Fit Group

Oneway Analysis of TotalDensity By Depth

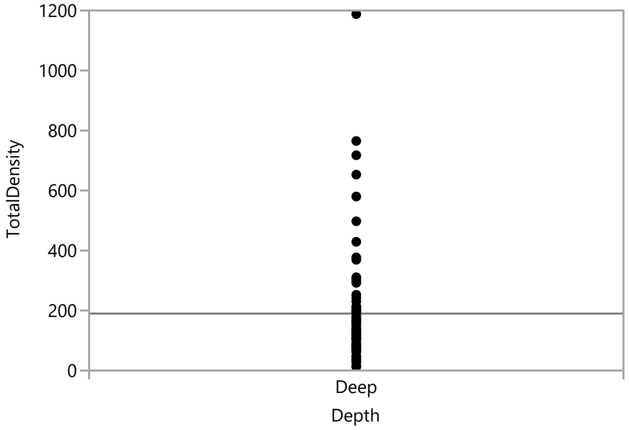

Oneway Analysis of Richness By Depth

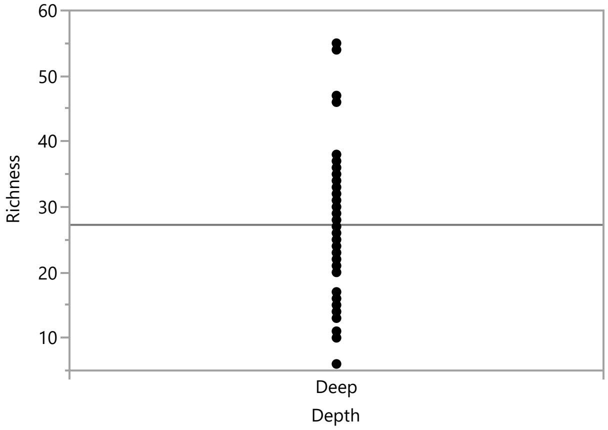

Where(:Ecoregion == "Deerfield" & :Type 2 == "Coral Reef" &
:Relief == "High")

S3 Density and Richness between depth by ecoregion, type, relief

Fit Group

Oneway Analysis of TotalDensity By Depth

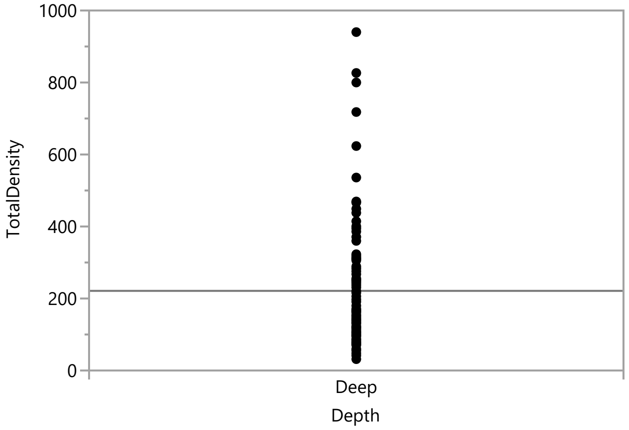

Oneway Analysis of Richness By Depth

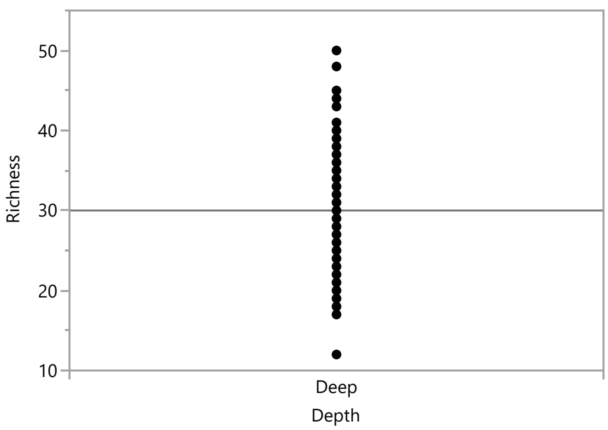

Where(:Ecoregion == "Deerfield" & :Type 2 == "Hardbottom" &
:Relief == "Low")

Fit Group

Oneway Analysis of TotalDensity By Depth

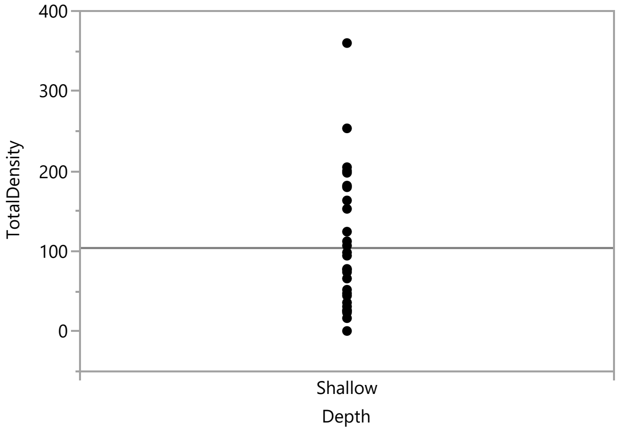

Oneway Analysis of Richness By Depth

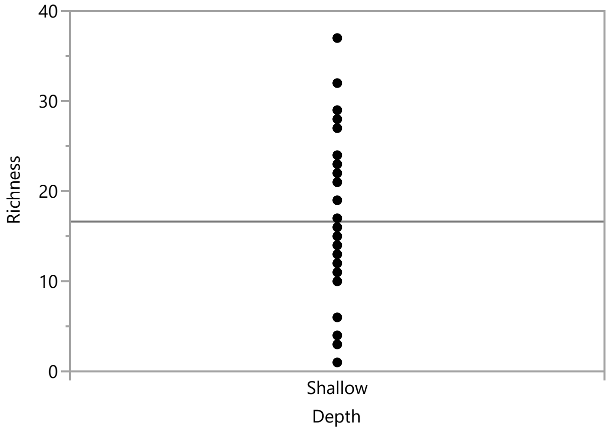

Where(:Ecoregion == "Martin" & :Type 2 == "Hardbottom" &
:Relief == "Low")

Fit Group

S3 Density and Richness between depth by ecoregion, type, relief

Fit Group

Oneway Analysis of TotalDensity By Depth

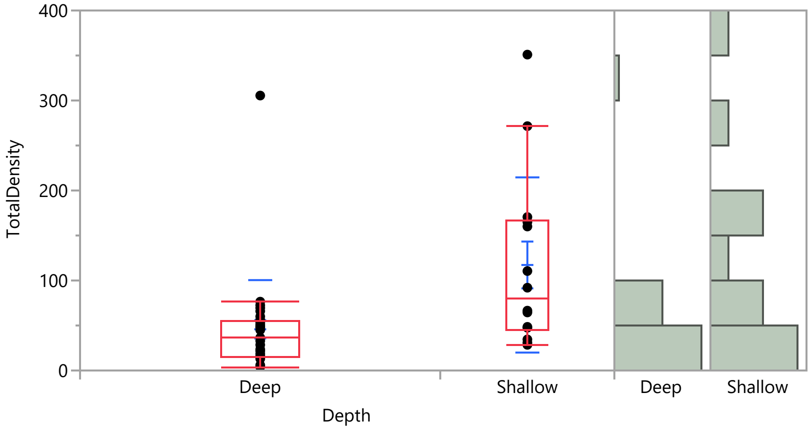

Means and Std Deviations

Level	Number	Mean	Std Dev	Std Err Mean	Lower 95%	Upper 95%
Deep	29	45.775862	54.648321	10.147939	24.988752	66.562972
Shallow	14	117.25	97.338179	26.014723	61.048608	173.45139

Wilcoxon / Kruskal-Wallis Tests (Rank Sums)

Level	Count	Score Sum	Expected Score	Score Mean	(Mean-Mean0)/Std0
Deep	29	522.000	638.000	18.0000	-2.994
Shallow	14	424.000	308.000	30.2857	2.994

2-Sample Test, Normal Approximation

S	Z	Prob>|Z|
424	2.99398	0.0028*

1-Way Test, ChiSquare Approximation

ChiSquare	DF	Prob>ChiSq
9.0417	1	0.0026*

S3 Density and Richness between depth by ecoregion, type, relief

Fit Group

Oneway Analysis of Richness By Depth

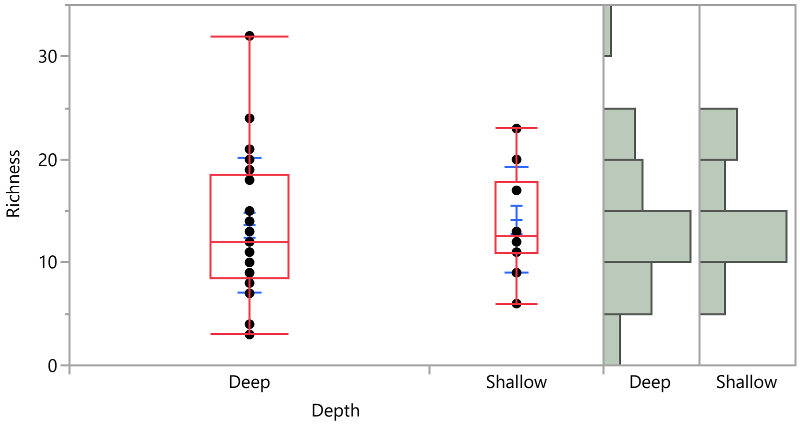

Means and Std Deviations

Level	Number	Mean	Std Dev	Std Err Mean	Lower 95%	Upper 95%
Deep	29	13.62069	6.5487937	1.2160805	11.129662	16.111718
Shallow	14	14.142857	5.1269596	1.3702376	11.182639	17.103075

Wilcoxon / Kruskal-Wallis Tests (Rank Sums)

Level	Count	Score Sum	Expected Score	Score Mean	(Mean-Mean0)/Std0
Deep	29	618.000	638.000	21.3103	-0.507
Shallow	14	328.000	308.000	23.4286	0.507

2-Sample Test, Normal Approximation

S	Z	Prob>|Z|
328	0.50711	0.6121

1-Way Test, ChiSquare Approximation

ChiSquare	DF	Prob>ChiSq
0.2705	1	0.6030

Where(:Ecoregion == "Martin" & :Type 2 == "Hardbottom" & :Relief == "High")

Fit Group

S3 Density and Richness between depth by ecoregion, type, relief

Fit Group

Oneway Analysis of TotalDensity By Depth

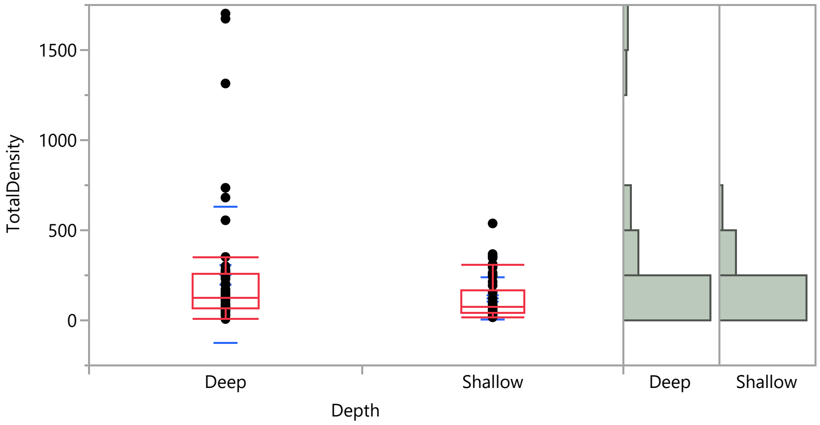

Means and Std Deviations

Level	Number	Mean	Std Dev	Std Err Mean	Lower 95%	Upper 95%
Deep	48	252.80208	377.84022	54.536538	143.08872	362.51545
Shallow	46	121.88043	117.29645	17.294418	87.047688	156.71318

Wilcoxon / Kruskal-Wallis Tests (Rank Sums)

Level	Count	Score Sum	Expected Score	Score Mean	(Mean-Mean0)/Std0
Deep	48	2576.00	2280.00	53.6667	2.235
Shallow	46	1889.00	2185.00	41.0652	-2.235

2-Sample Test, Normal Approximation

S	Z	Prob>|Z|
1889	-2.23511	0.0254*

1-Way Test, ChiSquare Approximation

ChiSquare	DF	Prob>ChiSq
5.0126	1	0.0252*

S3 Density and Richness between depth by ecoregion, type, relief

Fit Group

Oneway Analysis of Richness By Depth

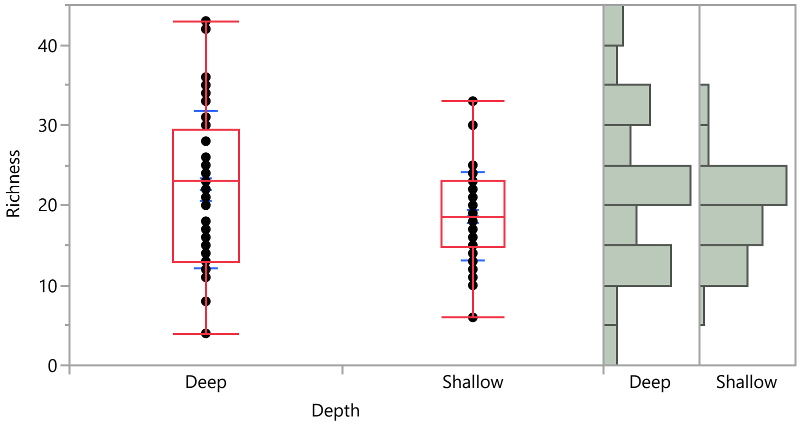

Means and Std Deviations

Level	Number	Mean	Std Dev	Std Err Mean	Lower 95%	Upper 95%
Deep	48	21.9375	9.8248626	1.4180968	19.084657	24.790343
Shallow	46	18.608696	5.5074828	0.812034	16.973175	20.244216

Wilcoxon / Kruskal-Wallis Tests (Rank Sums)

Level	Count	Score Sum	Expected Score	Score Mean	(Mean-Mean0)/Std0
Deep	48	2508.00	2280.00	52.2500	1.724
Shallow	46	1957.00	2185.00	42.5435	-1.724

2-Sample Test, Normal Approximation

S	Z	Prob>|Z|
1957	-1.72390	0.0847

1-Way Test, ChiSquare Approximation

ChiSquare	DF	Prob>ChiSq
2.9849	1	0.0840

Where(:Ecoregion == "North Palm Beach" & :Type 2 == "Coral Reef" & :Relief == "Low")

Fit Group

Oneway Analysis of TotalDensity By Depth

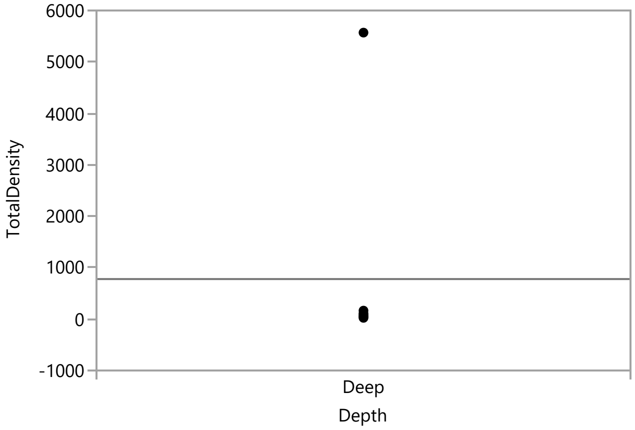

Oneway Analysis of Richness By Depth

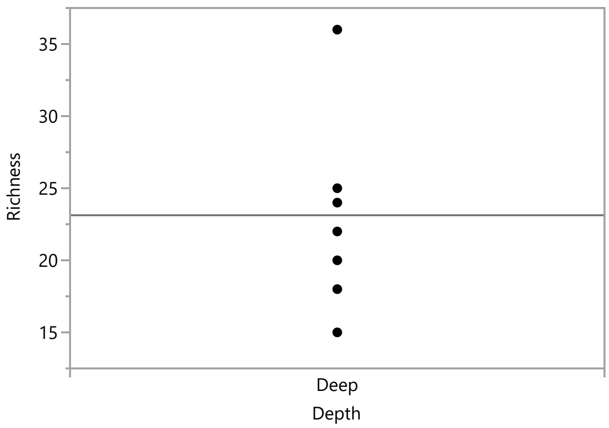

Where(:Ecoregion == "North Palm Beach" & :Type 2 == "Hardbottom" & :Relief == "Low")

S3 Density and Richness between depth by ecoregion, type, relief

Fit Group

Oneway Analysis of TotalDensity By Depth

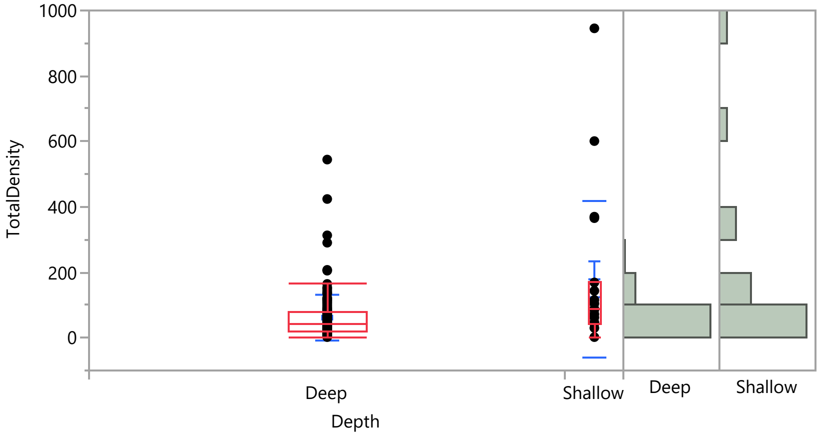

Means and Std Deviations

Level	Number	Mean	Std Dev	Std Err Mean	Lower 95%	Upper 95%
Deep	156	61.471154	69.916707	5.5978166	50.413299	72.529009
Shallow	19	178.68421	239.21934	54.880681	63.384177	293.98424

Wilcoxon / Kruskal-Wallis Tests (Rank Sums)

Level	Count	Score Sum	Expected Score	Score Mean	(Mean-Mean0)/Std0
Deep	156	13089.0	13728.0	83.904	-3.062
Shallow	19	2311.00	1672.00	121.632	3.062

2-Sample Test, Normal Approximation

S	Z	Prob>|Z|
2311	3.06245	0.0022*

1-Way Test, ChiSquare Approximation

ChiSquare	DF	Prob>ChiSq
9.3933	1	0.0022*

S3 Density and Richness between depth by ecoregion, type, relief

Fit Group

Oneway Analysis of Richness By Depth

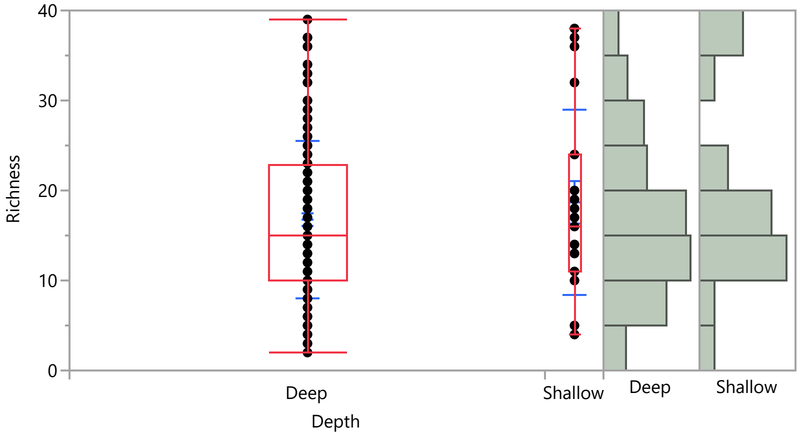

Means and Std Deviations

Level	Number	Mean	Std Dev	Std Err Mean	Lower 95%	Upper 95%
Deep	156	16.762821	8.7459574	0.700237	15.379581	18.14606
Shallow	19	18.684211	10.290517	2.3608065	13.72434	23.644081

Wilcoxon / Kruskal-Wallis Tests (Rank Sums)

Level	Count	Score Sum	Expected Score	Score Mean	(Mean-Mean0)/Std0
Deep	156	13582.0	13728.0	87.0641	-0.698
Shallow	19	1818.00	1672.00	95.6842	0.698

2-Sample Test, Normal Approximation

S	Z	Prob>|Z|
1818	0.69842	0.4849

1-Way Test, ChiSquare Approximation

ChiSquare	DF	Prob>ChiSq
0.4911	1	0.4834

Where(:Ecoregion == "North Palm Beach" & :Type 2 == "Hardbottom" & :Relief == "High")

Fit Group

Oneway Analysis of TotalDensity By Depth

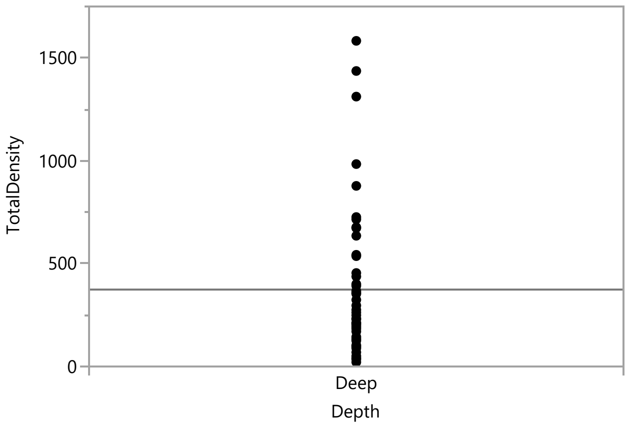

Oneway Analysis of Richness By Depth

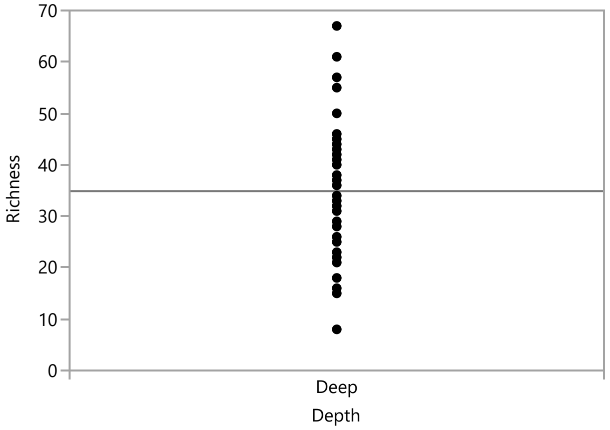

Where(:Ecoregion == "South Palm Beach" & :Type 2 == "Coral Reef" & :Relief == "Low")

S3 Density and Richness between depth by ecoregion, type, relief

Fit Group

Oneway Analysis of TotalDensity By Depth

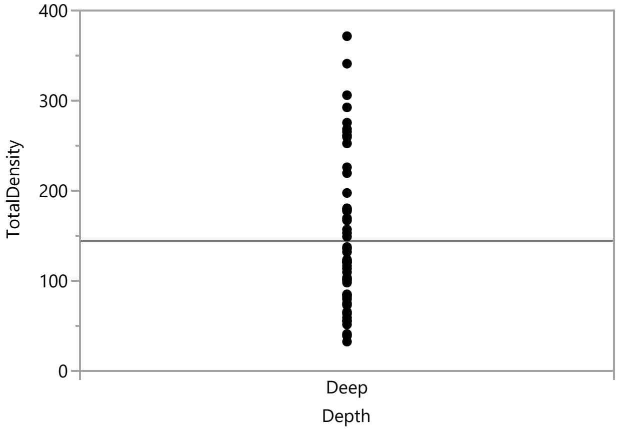

Oneway Analysis of Richness By Depth

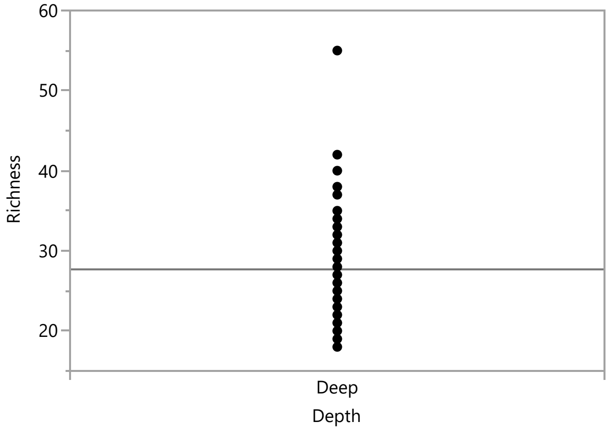

Where(:Ecoregion == "South Palm Beach" & :Type 2 == "Coral Reef" & :Relief == "High")

Fit Group

Oneway Analysis of TotalDensity By Depth

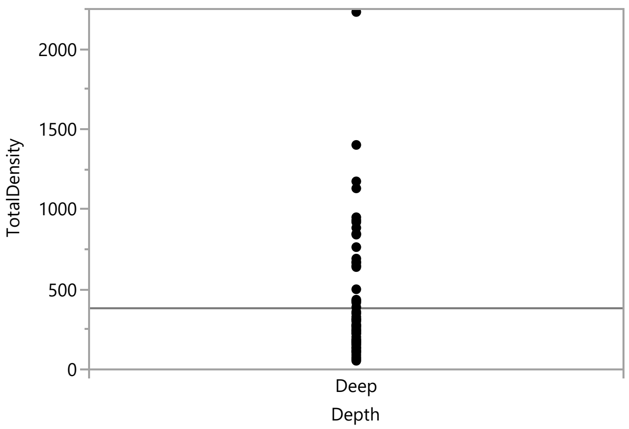

Oneway Analysis of Richness By Depth

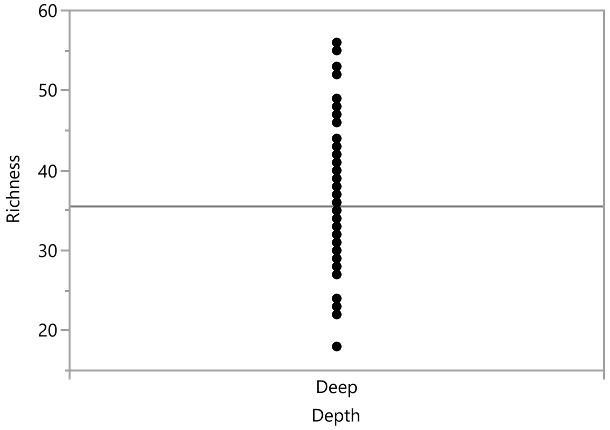

Where(:Ecoregion == "South Palm Beach" & :Type 2 == "Hardbottom" & :Relief == "Low")

Fit Group

S3 Density and Richness between depth by ecoregion, type, relief

Fit Group

Oneway Analysis of TotalDensity By Depth

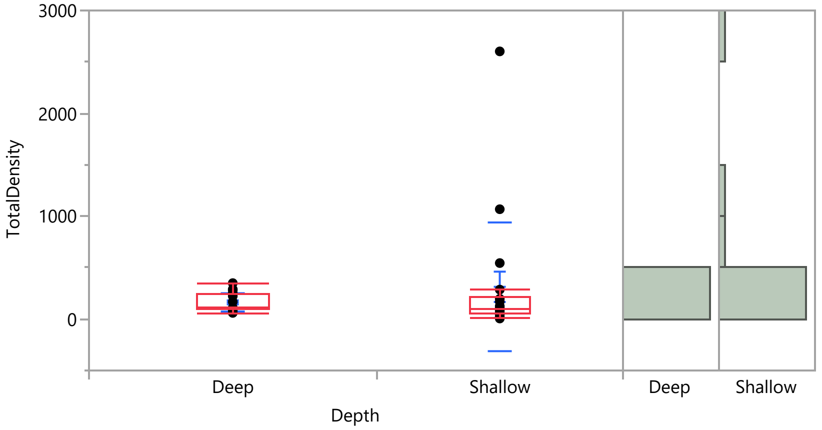

Means and Std Deviations

Level	Number	Mean	Std Dev	Std Err Mean	Lower 95%	Upper 95%
Deep	21	162.5119	88.791908	19.375983	122.09431	202.9295
Shallow	18	313.61111	625.68862	147.47622	2.4634795	624.75874

Wilcoxon / Kruskal-Wallis Tests (Rank Sums)

Level	Count	Score Sum	Expected Score	Score Mean	(Mean-Mean0)/Std0
Deep	21	463.000	420.000	22.0476	1.197
Shallow	18	317.000	360.000	17.6111	-1.197

2-Sample Test, Normal Approximation

S	Z	Prob>|Z|
317	-1.19730	0.2312

1-Way Test, ChiSquare Approximation

ChiSquare	DF	Prob>ChiSq
1.4675	1	0.2257

S3 Density and Richness between depth by ecoregion, type, relief

Fit Group

Oneway Analysis of Richness By Depth

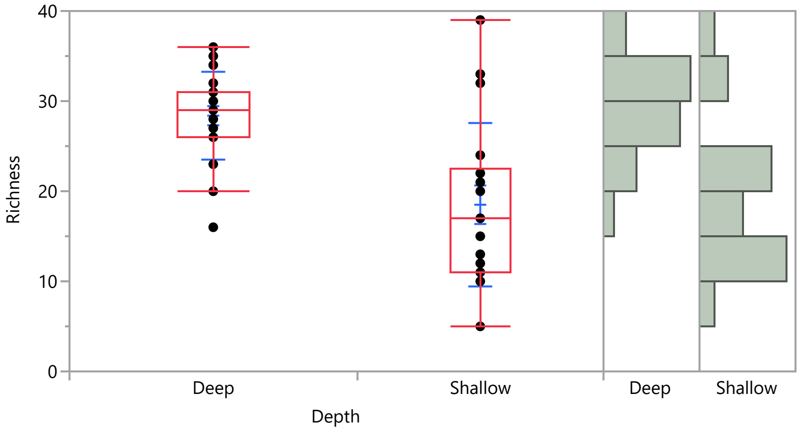

Means and Std Deviations

Level	Number	Mean	Std Dev	Std Err Mean	Lower 95%	Upper 95%
Deep	21	28.380952	4.8731529	1.0634091	26.16272	30.599185
Shallow	18	18.5	9.0699893	2.137817	13.9896	23.0104

Wilcoxon / Kruskal-Wallis Tests (Rank Sums)

Level	Count	Score Sum	Expected Score	Score Mean	(Mean-Mean0)/Std0
Deep	21	539.000	420.000	25.6667	3.342
Shallow	18	241.000	360.000	13.3889	-3.342

2-Sample Test, Normal Approximation

S	Z	Prob>|Z|
241	-3.34191	0.0008*

1-Way Test, ChiSquare Approximation

ChiSquare	DF	Prob>ChiSq
11.2628	1	0.0008*
