# Supplementary material for: Regional reef fish assemblage maps provide baseline biogeography for tropicalization monitoring
Source: Sci Rep. 2024 Apr 3;14:7893. doi: 10.1038/s41598-024-58185-6 (PMC10991435; doi:10.1038/s41598-024-58185-6)

S4 Density and Richness between habitat type by ecoregion, depth, relief

Where(:Ecoregion == "Broward-Miami" & :Depth == "Deep" & :Relief == "Low")

Fit Group

Oneway Analysis of TotalDensity By Type 2

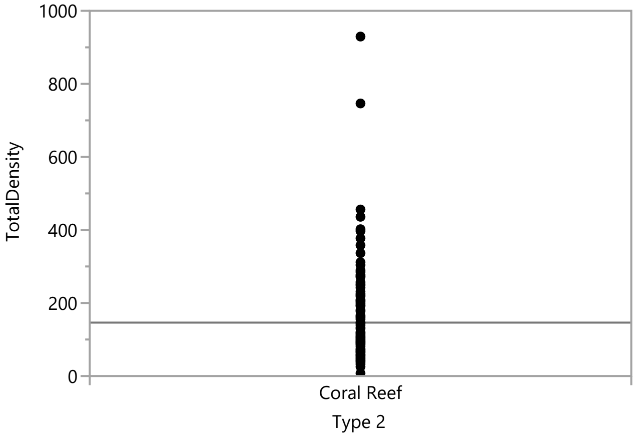

Oneway Analysis of Richness By Type 2

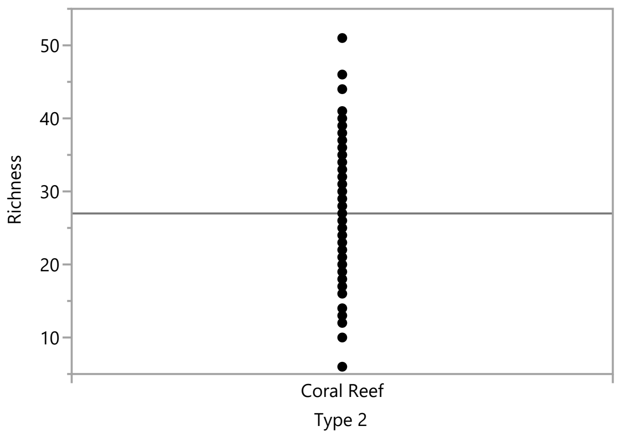

Where(:Ecoregion == "Broward-Miami" & :Depth == "Deep" & :Relief == "High")

Fit Group

Oneway Analysis of TotalDensity By Type 2

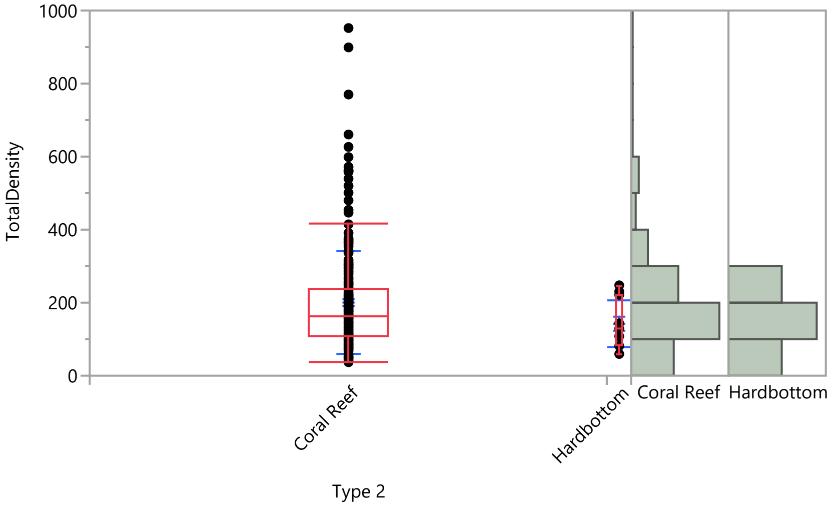

Means and Std Deviations

Level	Number	Mean	Std Dev	Std Err Mean	Lower 95%	Upper 95%
Coral Reef	239	200.25131	140.5426	9.0909452	182.34232	218.16031
Hardbottom	11	142.31818	63.919978	19.272599	99.376156	185.26021

Wilcoxon / Kruskal-Wallis Tests (Rank Sums)

Level	Count	Score Sum	Expected Score	Score Mean	(Mean-Mean0)/Std0
Coral Reef	239	30297.0	29994.5	126.766	1.288
Hardbottom	11	1078.00	1380.50	98.000	-1.288

2-Sample Test, Normal Approximation

S	Z	Prob>|Z|
1078	-1.28786	0.1978

1-Way Test, ChiSquare Approximation

ChiSquare	DF	Prob>ChiSq
1.6641	1	0.1971

S4 Density and Richness between habitat type by ecoregion, depth, relief

Fit Group

Oneway Analysis of Richness By Type 2

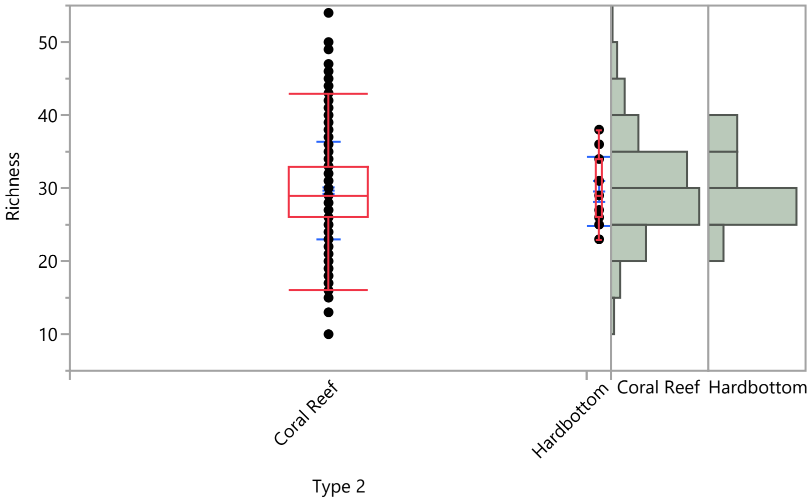

Means and Std Deviations

Level	Number	Mean	Std Dev	Std Err Mean	Lower 95%	Upper 95%
Coral Reef	239	29.669456	6.6921775	0.432881	28.816689	30.522224
Hardbottom	11	29.545455	4.7405408	1.4293268	26.360716	32.730193

Wilcoxon / Kruskal-Wallis Tests (Rank Sums)

Level	Count	Score Sum	Expected Score	Score Mean	(Mean-Mean0)/Std0
Coral Reef	239	30011.5	29994.5	125.571	0.070
Hardbottom	11	1363.50	1380.50	123.955	-0.070

2-Sample Test, Normal Approximation

S	Z	Prob>|Z|
1363.5	-0.07047	0.9438

1-Way Test, ChiSquare Approximation

ChiSquare	DF	Prob>ChiSq
0.0053	1	0.9421

Where(:Ecoregion == "Broward-Miami" & :Depth == "Shallow" & :Relief == "Low")

Fit Group

S4 Density and Richness between habitat type by ecoregion, depth, relief

Fit Group

Oneway Analysis of TotalDensity By Type 2

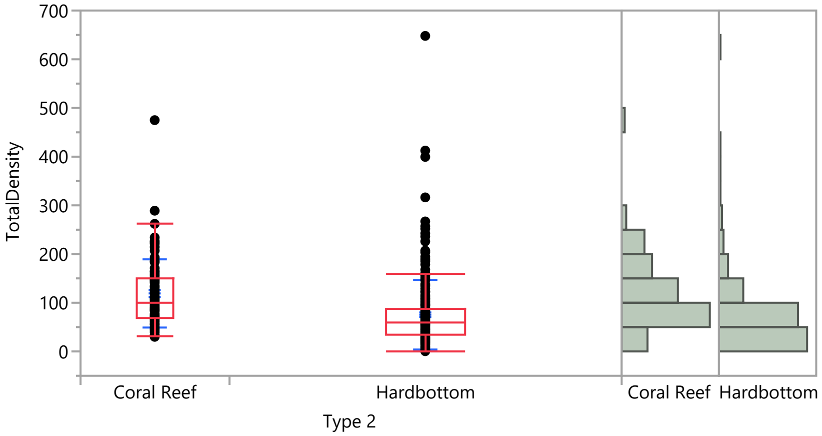

Means and Std Deviations

Level	Number	Mean	Std Dev	Std Err Mean	Lower 95%	Upper 95%
Coral Reef	91	119.08086	70.017025	7.3397786	104.49911	133.66261
Hardbottom	241	75.437178	71.523387	4.6072264	66.361414	84.512943

Wilcoxon / Kruskal-Wallis Tests (Rank Sums)

Level	Count	Score Sum	Expected Score	Score Mean	(Mean-Mean0)/Std0
Coral Reef	91	20418.5	15151.5	224.379	6.751
Hardbottom	241	34859.5	40126.5	144.645	-6.751

2-Sample Test, Normal Approximation

S	Z	Prob>|Z|
20418.5	6.75102	<.0001*

1-Way Test, ChiSquare Approximation

ChiSquare	DF	Prob>ChiSq
45.5850	1	<.0001*

S4 Density and Richness between habitat type by ecoregion, depth, relief

Fit Group

Oneway Analysis of Richness By Type 2

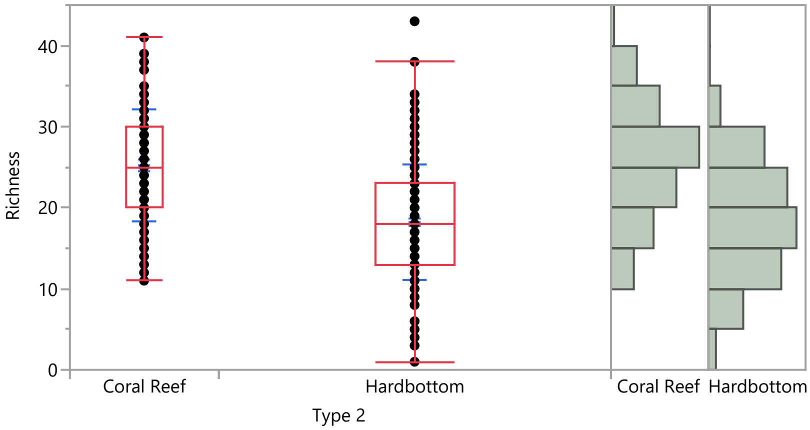

Means and Std Deviations

Level	Number	Mean	Std Dev	Std Err Mean	Lower 95%	Upper 95%
Coral Reef	91	25.230769	6.9074467	0.7240972	23.792224	26.669315
Hardbottom	241	18.215768	7.1229617	0.4588303	17.311919	19.119616

Wilcoxon / Kruskal-Wallis Tests (Rank Sums)

Level	Count	Score Sum	Expected Score	Score Mean	(Mean-Mean0)/Std0
Coral Reef	91	20794.5	15151.5	228.511	7.239
Hardbottom	241	34483.5	40126.5	143.085	-7.239

2-Sample Test, Normal Approximation

S	Z	Prob>|Z|
20794.5	7.23910	<.0001*

1-Way Test, ChiSquare Approximation

ChiSquare	DF	Prob>ChiSq
52.4139	1	<.0001*

Where(:Ecoregion == "Broward-Miami" & :Depth == "Shallow" & :Relief == "High")

Fit Group

S4 Density and Richness between habitat type by ecoregion, depth, relief

Fit Group

Oneway Analysis of TotalDensity By Type 2

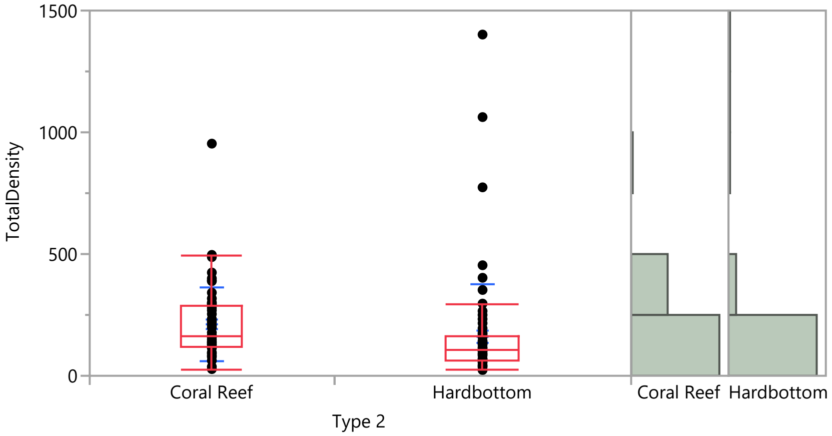

Means and Std Deviations

Level	Number	Mean	Std Dev	Std Err Mean	Lower 95%	Upper 95%
Coral Reef	60	211.41875	151.4655	19.554112	172.29106	250.54644
Hardbottom	73	160.28082	215.74979	25.251603	109.94267	210.61897

Wilcoxon / Kruskal-Wallis Tests (Rank Sums)

Level	Count	Score Sum	Expected Score	Score Mean	(Mean-Mean0)/Std0
Coral Reef	60	4894.00	4020.00	81.5667	3.950
Hardbottom	73	4017.00	4891.00	55.0274	-3.950

2-Sample Test, Normal Approximation

S	Z	Prob>|Z|
4894	3.94979	<.0001*

1-Way Test, ChiSquare Approximation

ChiSquare	DF	Prob>ChiSq
15.6187	1	<.0001*

S4 Density and Richness between habitat type by ecoregion, depth, relief

Fit Group

Oneway Analysis of Richness By Type 2

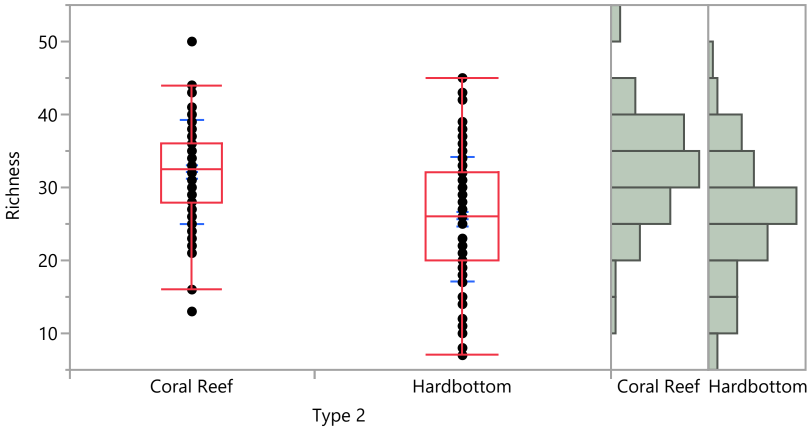

Means and Std Deviations

Level	Number	Mean	Std Dev	Std Err Mean	Lower 95%	Upper 95%
Coral Reef	60	32.116667	7.1357103	0.9212162	30.273317	33.960016
Hardbottom	73	25.643836	8.5332192	0.9987378	23.652888	27.634783

Wilcoxon / Kruskal-Wallis Tests (Rank Sums)

Level	Count	Score Sum	Expected Score	Score Mean	(Mean-Mean0)/Std0
Coral Reef	60	4991.50	4020.00	83.1917	4.394
Hardbottom	73	3919.50	4891.00	53.6918	-4.394

2-Sample Test, Normal Approximation

S	Z	Prob>|Z|
4991.5	4.39436	<.0001*

1-Way Test, ChiSquare Approximation

ChiSquare	DF	Prob>ChiSq
19.3303	1	<.0001*

Where(:Ecoregion == "Deerfield" & :Depth == "Deep" & :Relief == "Low")

S4 Density and Richness between habitat type by ecoregion, depth, relief

Fit Group

Oneway Analysis of TotalDensity By Type 2

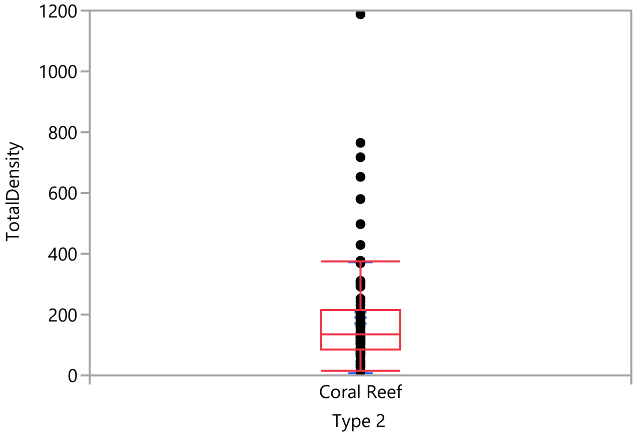

Means and Std Deviations

Level	Number	Mean	Std Dev	Std Err Mean	Lower 95%	Upper 95%
Coral Reef	88	189.78843	182.42862	19.446957	151.1355	228.44136

Wilcoxon / Kruskal-Wallis Tests (Rank Sums)

Level	Count	Score Sum	Expected Score	Score Mean	(Mean-Mean0)/Std0
Coral Reef	88	3916.00	3916.00	44.5000	.

1-Way Test, ChiSquare Approximation

ChiSquare	DF	Prob>ChiSq
0.0000	0	.

Oneway Analysis of Richness By Type 2

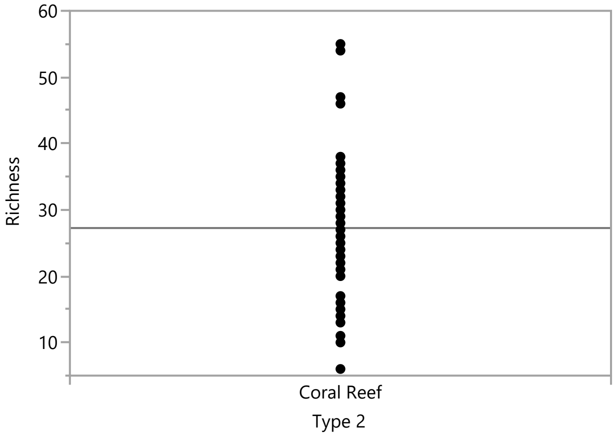

Where(:Ecoregion == "Deerfield" & :Depth == "Deep" & :Relief == "High")

S4 Density and Richness between habitat type by ecoregion, depth, relief

Fit Group

Oneway Analysis of TotalDensity By Type 2

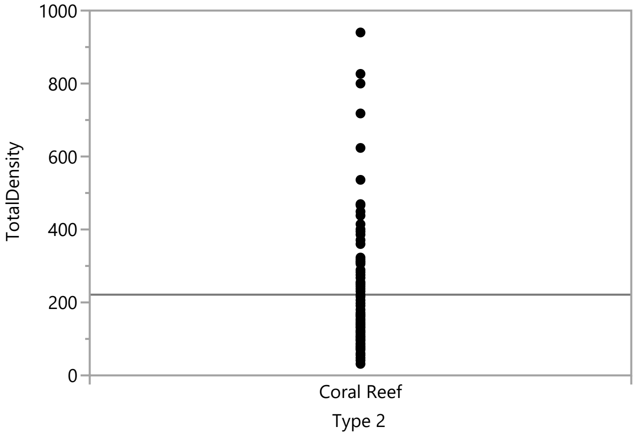

Oneway Analysis of Richness By Type 2

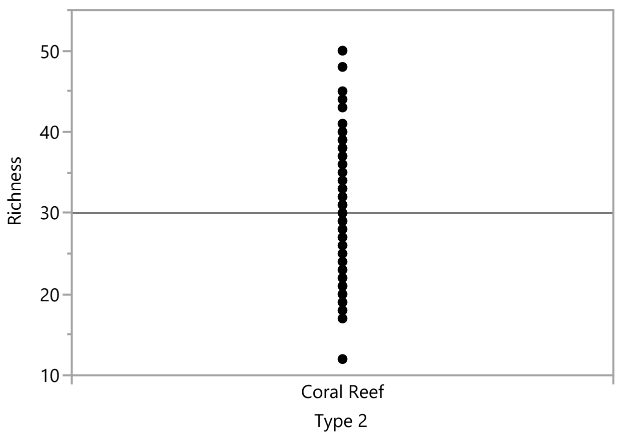

Where(:Ecoregion == "Deerfield" & :Depth == "Shallow" & :Relief == "Low")

Fit Group

Oneway Analysis of TotalDensity By Type 2

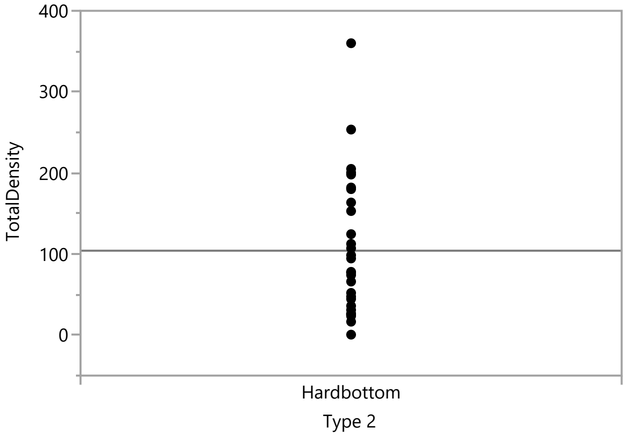

Oneway Analysis of Richness By Type 2

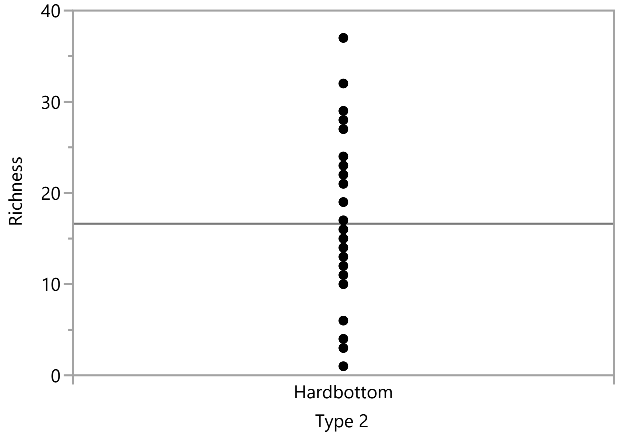

Where(:Ecoregion == "Martin" & :Depth == "Deep" & :Relief == "Low")

Fit Group

Oneway Analysis of TotalDensity By Type 2

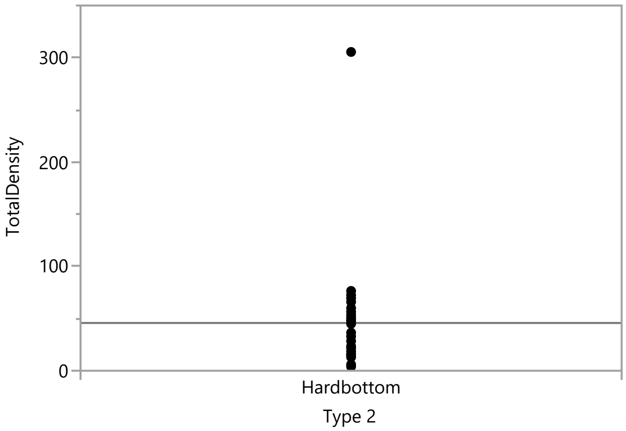

Oneway Analysis of Richness By Type 2

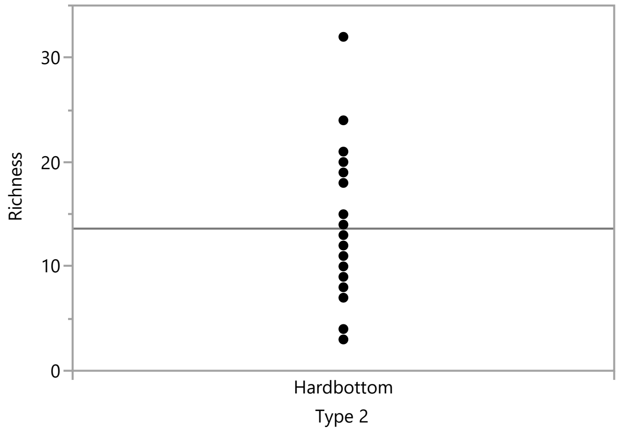

Where(:Ecoregion == "Martin" & :Depth == "Deep" & :Relief == "High")

S4 Density and Richness between habitat type by ecoregion, depth, relief

Fit Group

Oneway Analysis of TotalDensity By Type 2

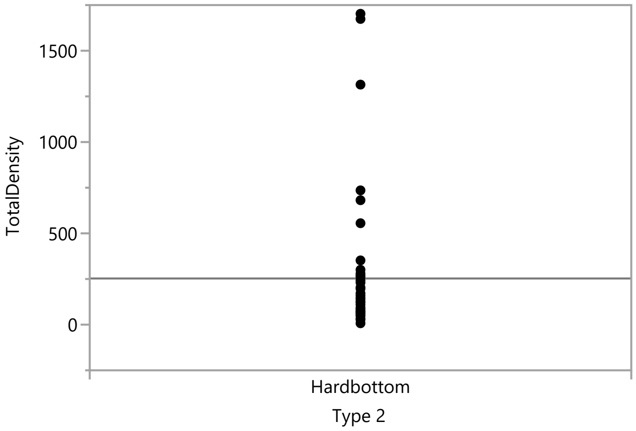

Oneway Analysis of Richness By Type 2

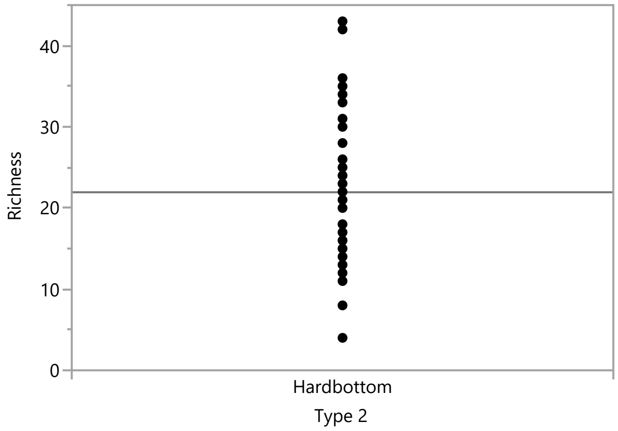

Where(:Ecoregion == "Martin" & :Depth == "Shallow" & :Relief == "Low")

Fit Group

Oneway Analysis of TotalDensity By Type 2

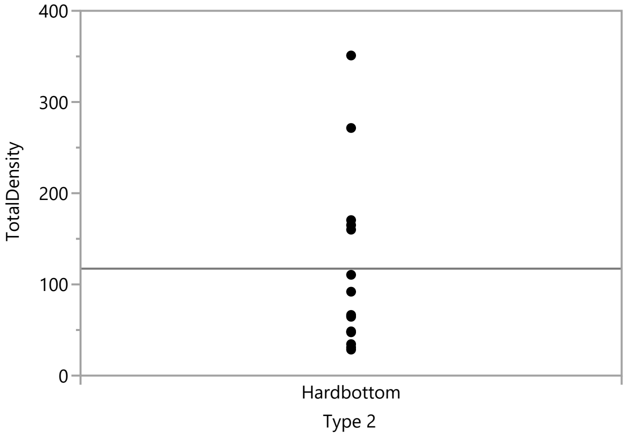

Oneway Analysis of Richness By Type 2

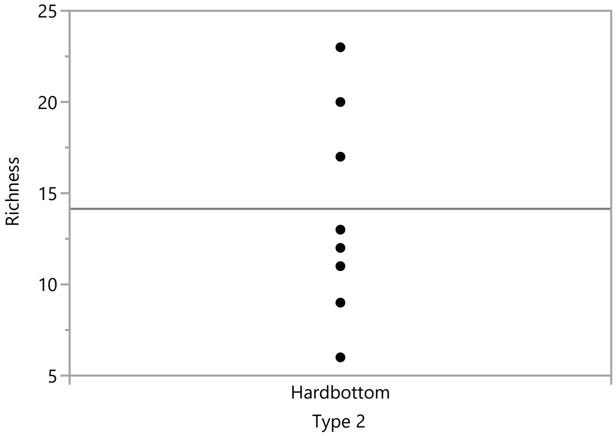

Where(:Ecoregion == "Martin" & :Depth == "Shallow" & :Relief == "High")

Fit Group

Oneway Analysis of TotalDensity By Type 2

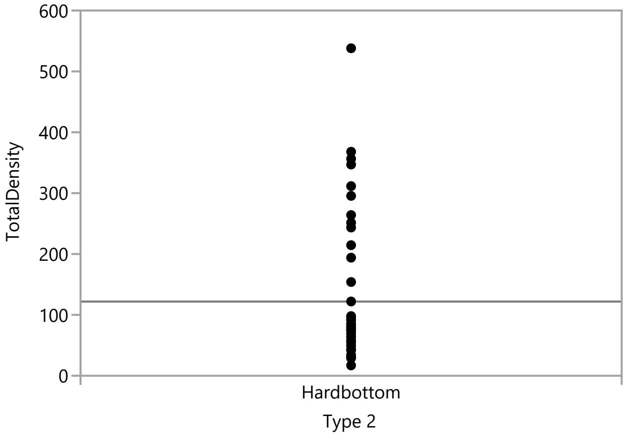

Oneway Analysis of Richness By Type 2

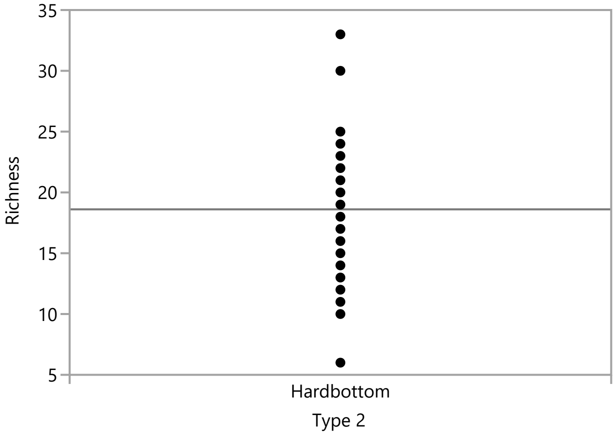

Where(:Ecoregion == "North Palm Beach" & :Depth == "Deep" & :Relief == "Low")

S4 Density and Richness between habitat type by ecoregion, depth, relief

Fit Group

Oneway Analysis of TotalDensity By Type 2

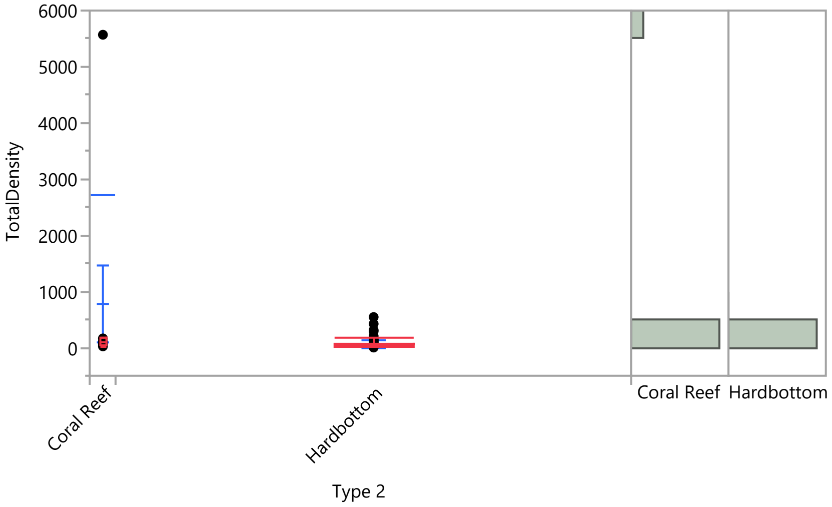

Means and Std Deviations

Level	Number	Mean	Std Dev	Std Err Mean	Lower 95%	Upper 95%
Coral Reef	8	777.8125	1937.0508	684.85087	-841.6025	2397.2275
Hardbottom	156	61.471154	69.916707	5.5978166	50.413299	72.529009

Wilcoxon / Kruskal-Wallis Tests (Rank Sums)

Level	Count	Score Sum	Expected Score	Score Mean	(Mean-Mean0)/Std0
Coral Reef	8	973.500	660.000	121.688	2.389
Hardbottom	156	12556.5	12870.0	80.490	-2.389

2-Sample Test, Normal Approximation

S	Z	Prob>|Z|
973.5	2.38948	0.0169*

1-Way Test, ChiSquare Approximation

ChiSquare	DF	Prob>ChiSq
5.7279	1	0.0167*

S4 Density and Richness between habitat type by ecoregion, depth, relief

Fit Group

Oneway Analysis of Richness By Type 2

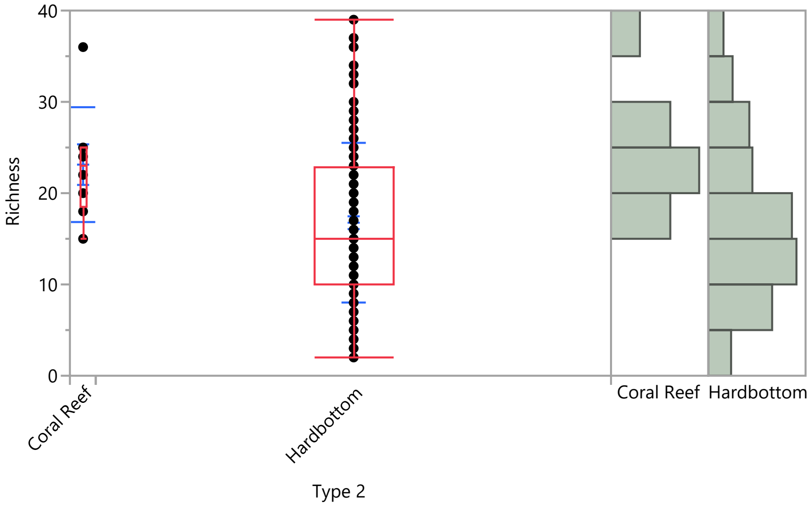

Means and Std Deviations

Level	Number	Mean	Std Dev	Std Err Mean	Lower 95%	Upper 95%
Coral Reef	8	23.125	6.289163	2.2235549	17.867128	28.382872
Hardbottom	156	16.762821	8.7459574	0.700237	15.379581	18.14606

Wilcoxon / Kruskal-Wallis Tests (Rank Sums)

Level	Count	Score Sum	Expected Score	Score Mean	(Mean-Mean0)/Std0
Coral Reef	8	955.000	660.000	119.375	2.250
Hardbottom	156	12575.0	12870.0	80.609	-2.250

2-Sample Test, Normal Approximation

S	Z	Prob>|Z|
955	2.24993	0.0245*

1-Way Test, ChiSquare Approximation

ChiSquare	DF	Prob>ChiSq
5.0794	1	0.0242*

Where(:Ecoregion == "North Palm Beach" & :Depth == "Deep" &
:Relief == "High")

Fit Group

Oneway Analysis of TotalDensity By Type 2

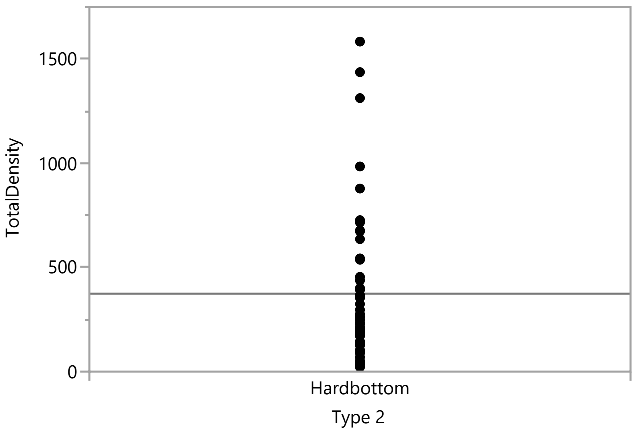

Oneway Analysis of Richness By Type 2

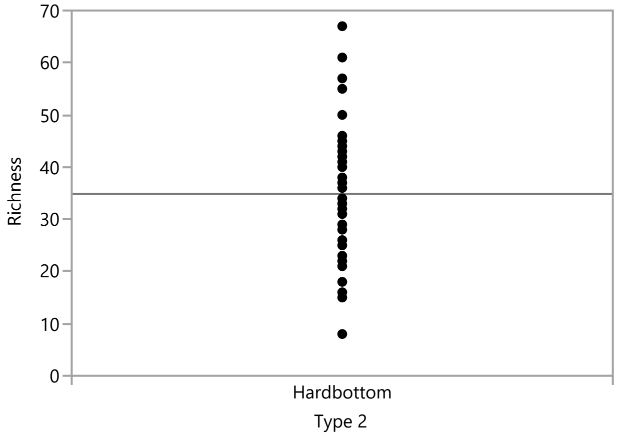

Where(:Ecoregion == "North Palm Beach" & :Depth == "Shallow" &
:Relief == "Low")

S4 Density and Richness between habitat type by ecoregion, depth, relief

Fit Group

Oneway Analysis of TotalDensity By Type 2

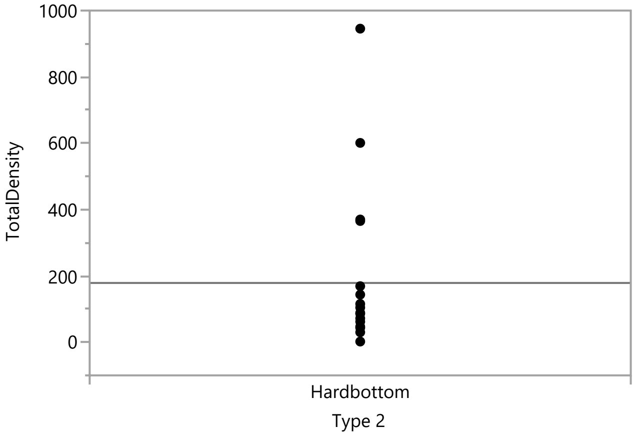

Oneway Analysis of Richness By Type 2

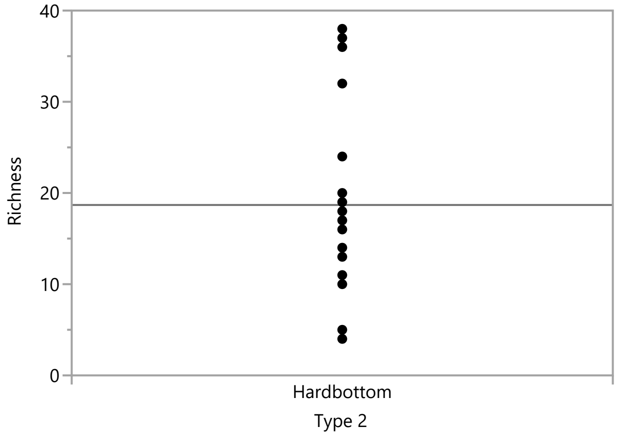

Where(:Ecoregion == "South Palm Beach" & :Depth == "Deep" & :Relief == "Low")

Fit Group

Oneway Analysis of TotalDensity By Type 2

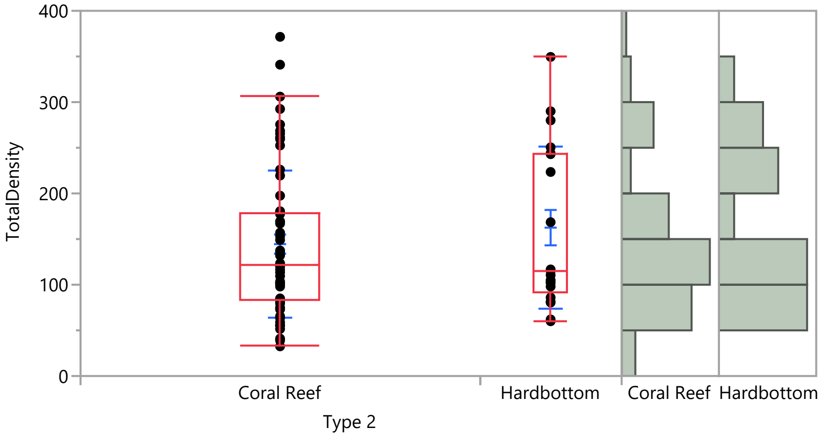

Means and Std Deviations

Level	Number	Mean	Std Dev	Std Err Mean	Lower 95%	Upper 95%
Coral Reef	59	144.47458	80.591906	10.492173	123.47221	165.47694
Hardbottom	21	162.5119	88.791908	19.375983	122.09431	202.9295

Wilcoxon / Kruskal-Wallis Tests (Rank Sums)

Level	Count	Score Sum	Expected Score	Score Mean	(Mean-Mean0)/Std0
Coral Reef	59	2342.00	2389.50	39.6949	-0.514
Hardbottom	21	898.000	850.500	42.7619	0.514

2-Sample Test, Normal Approximation

S	Z	Prob>|Z|
898	0.51395	0.6073

1-Way Test, ChiSquare Approximation

ChiSquare	DF	Prob>ChiSq
0.2698	1	0.6035

S4 Density and Richness between habitat type by ecoregion, depth, relief

Fit Group

Oneway Analysis of Richness By Type 2

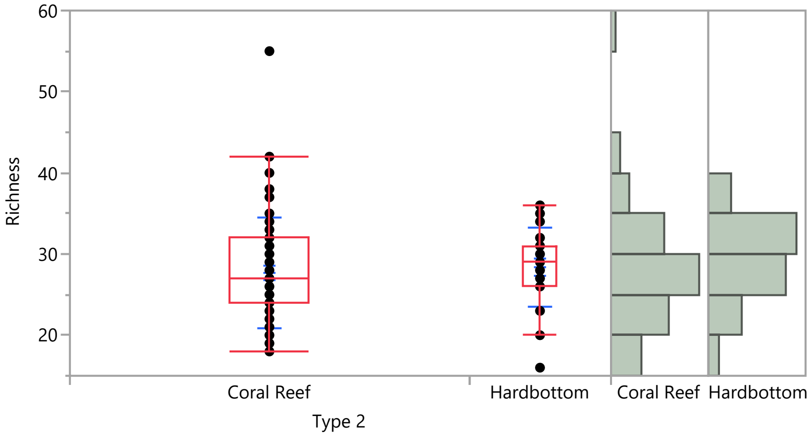

Means and Std Deviations

Level	Number	Mean	Std Dev	Std Err Mean	Lower 95%	Upper 95%
Coral Reef	59	27.677966	6.8214688	0.8880796	25.900282	29.455651
Hardbottom	21	28.380952	4.8731529	1.0634091	26.16272	30.599185

Wilcoxon / Kruskal-Wallis Tests (Rank Sums)

Level	Count	Score Sum	Expected Score	Score Mean	(Mean-Mean0)/Std0
Coral Reef	59	2297.00	2389.50	38.9322	-1.008
Hardbottom	21	943.000	850.500	44.9048	1.008

2-Sample Test, Normal Approximation

S	Z	Prob>|Z|
943	1.00787	0.3135

1-Way Test, ChiSquare Approximation

ChiSquare	DF	Prob>ChiSq
1.0269	1	0.3109

Where(:Ecoregion == "South Palm Beach" & :Depth == "Deep" & :Relief == "High")

Fit Group

Oneway Analysis of TotalDensity By Type 2

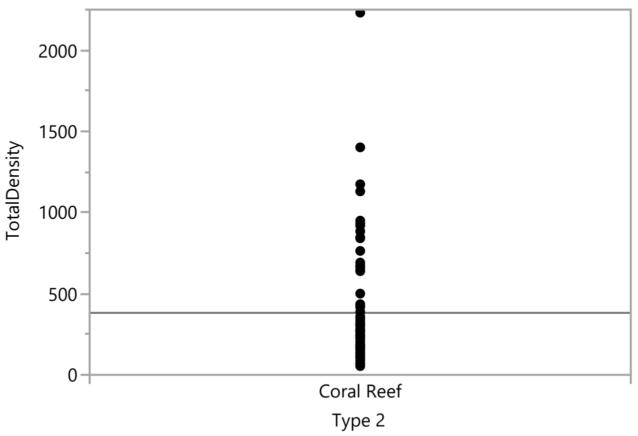

Oneway Analysis of Richness By Type 2

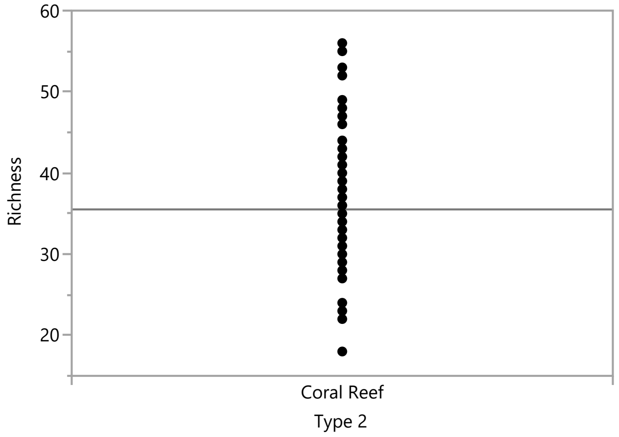

Where(:Ecoregion == "South Palm Beach" & :Depth == "Shallow" & :Relief == "Low")

S4 Density and Richness between habitat type by ecoregion, depth, relief

Fit Group

Oneway Analysis of TotalDensity By Type 2

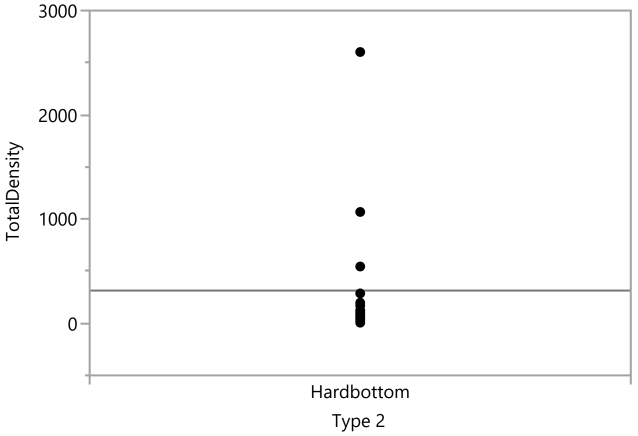

Oneway Analysis of Richness By Type 2

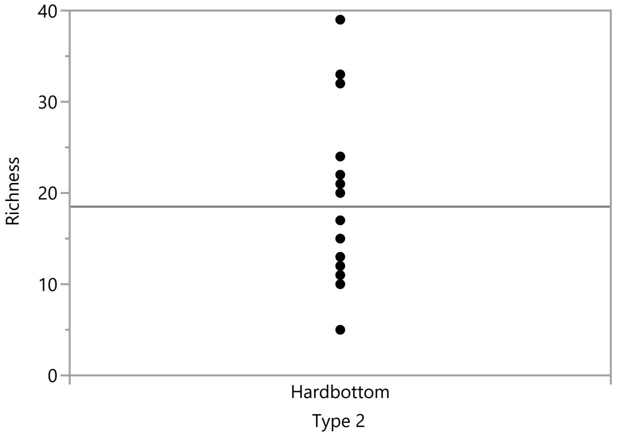

Supplement: Supplementary file 4 — Supplementary Information 4. [file 41598_2024_58185_MOESM4_ESM.pdf]
