# Supplementary material for: Regional reef fish assemblage maps provide baseline biogeography for tropicalization monitoring
Source: Sci Rep. 2024 Apr 3;14:7893. doi: 10.1038/s41598-024-58185-6 (PMC10991435; doi:10.1038/s41598-024-58185-6)
Supplement: Supplementary file 5 — Supplementary Information 5. [file 41598_2024_58185_MOESM5_ESM.pdf]

S5 Density and Richness between ecoregion by depth, type, relief

Where(:Depth == "Deep" & :Type 2 == "Coral Reef" & :Relief == "Low")

Fit Group

Oneway Analysis of TotalDensity By Ecoregion

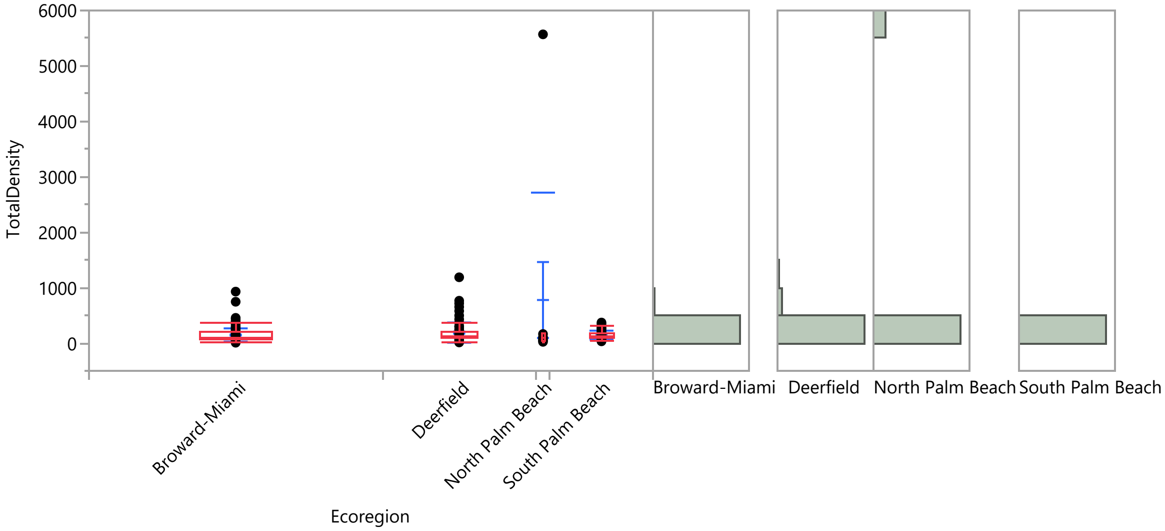

Means and Std Deviations

Level	Number	Mean	Std Dev	Std Err Mean	Lower 95%	Upper 95%
Broward-Miami	168	146.2691	119.83698	9.2456239	128.01573	164.52247
Deerfield	88	189.78843	182.42862	19.446957	151.1355	228.44136
North Palm Beach	8	777.8125	1937.0508	684.85087	-841.6025	2397.2275
South Palm Beach	59	144.47458	80.591906	10.492173	123.47221	165.47694

Nonparametric Comparisons For Each Pair Using Wilcoxon Method

q*	Alpha
1.95996	0.05

Level	- Level	Score Mean Difference	Std Err Dif	Z	p-Value	Hodges-Lehmann	Lower CL	Upper CL	Difference Plot
Deerfield	Broward-Miami	19.2987	9.74351	1.98067	0.0476*	22.0000	0.009	45.50000	
South Palm Beach	Broward-Miami	10.7065	9.93836	1.07729	0.2814	11.0000	-10.500	31.50000	
South Palm Beach	North Palm Beach	6.4587	7.34092	0.87982	0.3790	24.2500	-41.000	86.50000	
South Palm Beach	Deerfield	-6.7243	7.16448	-0.93856	0.3480	-13.2500	-42.000	14.50000	
North Palm Beach	Broward-Miami	-10.1488	18.43743	-0.55045	0.5820	-16.0000	-71.500	46.50000	
North Palm Beach	Deerfield	-11.9318	10.28669	-1.15993	0.2461	-37.5000	-107.500	34.50000	

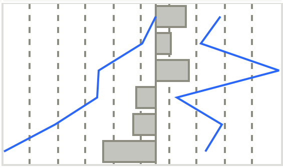

S5 Density and Richness between ecoregion by depth, type, relief

Fit Group

Oneway Analysis of Richness By Ecoregion

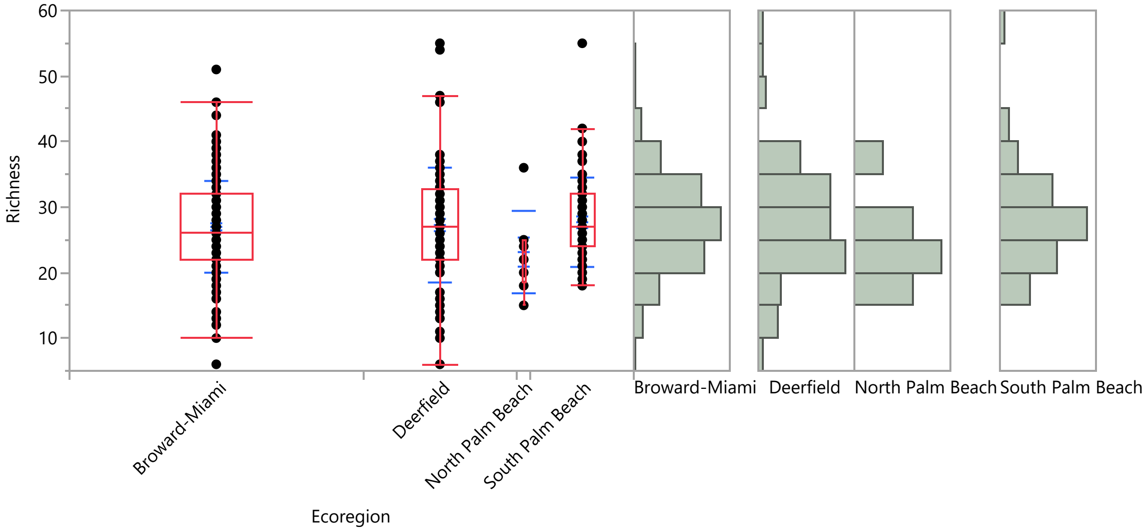

Means and Std Deviations

Level	Number	Mean	Std Dev	Std Err Mean	Lower 95%	Upper 95%
Broward-Miami	168	26.982143	6.9999771	0.54006	25.915918	28.048368
Deerfield	88	27.25	8.7641511	0.9342616	25.393054	29.106946
North Palm Beach	8	23.125	6.289163	2.2235549	17.867128	28.382872
South Palm Beach	59	27.677966	6.8214688	0.8880796	25.900282	29.455651

Nonparametric Comparisons For Each Pair Using Wilcoxon Method

q*	Alpha
1.95996	0.05

Level	- Level	Score Mean Difference	Std Err Dif	Z	p-Value	Hodges-Lehmann	Lower CL	Upper CL	Difference Plot
South Palm Beach	North Palm Beach	13.9820	7.32266	1.90942	0.0562	4.00000	0.0000	9.000000	
South Palm Beach	Broward-Miami	3.3894	9.92612	0.34147	0.7328	0.00000	-2.0000	2.000000	
Deerfield	Broward-Miami	1.8788	9.73333	0.19303	0.8469	0.00000	-2.0000	2.000000	
South Palm Beach	Deerfield	1.3024	7.15485	0.18203	0.8556	0.00000	-2.0000	3.000000	
North Palm Beach	Deerfield	-15.6818	10.27283	-1.52653	0.1269	-4.00000	-10.0000	1.000000	
North Palm Beach	Broward-Miami	-31.8214	18.41283	-1.72822	0.0839	-4.00000	-9.0000	1.000000	

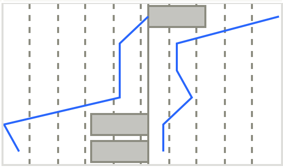

Where(:Depth == "Deep" & :Type 2 == "Coral Reef" & :Relief == "High")

Fit Group

S5 Density and Richness between ecoregion by depth, type, relief

Fit Group

Oneway Analysis of TotalDensity By Ecoregion

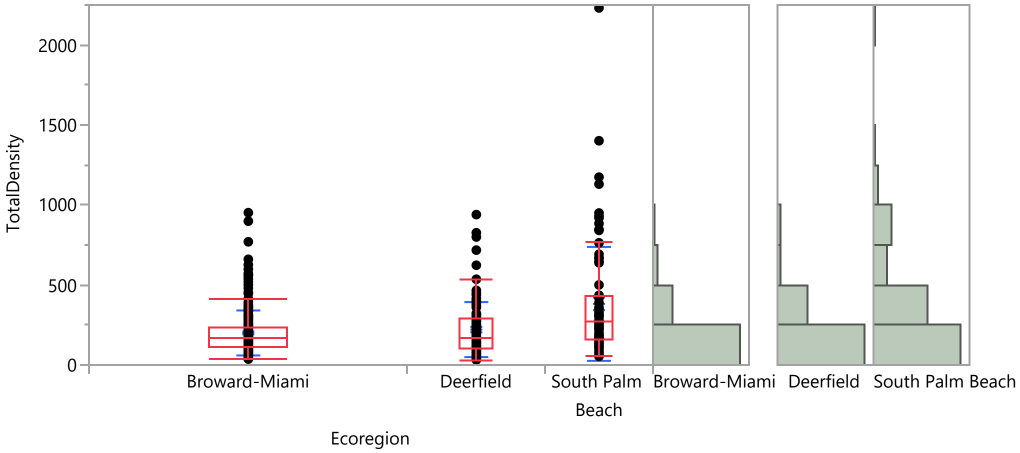

Means and Std Deviations

Level	Number	Mean	Std Dev	Std Err Mean	Lower 95%	Upper 95%
Broward-Miami	239	200.25131	140.5426	9.0909452	182.34232	218.16031
Deerfield	103	221.2233	171.91067	16.938862	187.62515	254.82145
South Palm Beach	81	381.96605	356.02872	39.558747	303.24163	460.69047

Nonparametric Comparisons For Each Pair Using Wilcoxon Method

q*	Alpha
1.95996	0.05

Level	- Level	Score Mean Difference	Std Err Dif	Z	p-Value	Hodges-Lehmann	Lower CL	Upper CL	Difference Plot
South Palm Beach	Broward-Miami	63.69751	11.89507	5.354949	<.0001*	89.50000	56.0000	130.1860	
South Palm Beach	Deerfield	31.29522	7.90948	3.956672	<.0001*	81.00000	41.0000	130.0000	
Deerfield	Broward-Miami	6.17537	11.65364	0.529909	0.5962	6.00000	-16.5000	29.0000	

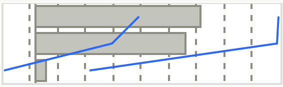

S5 Density and Richness between ecoregion by depth, type, relief

Fit Group

Oneway Analysis of Richness By Ecoregion

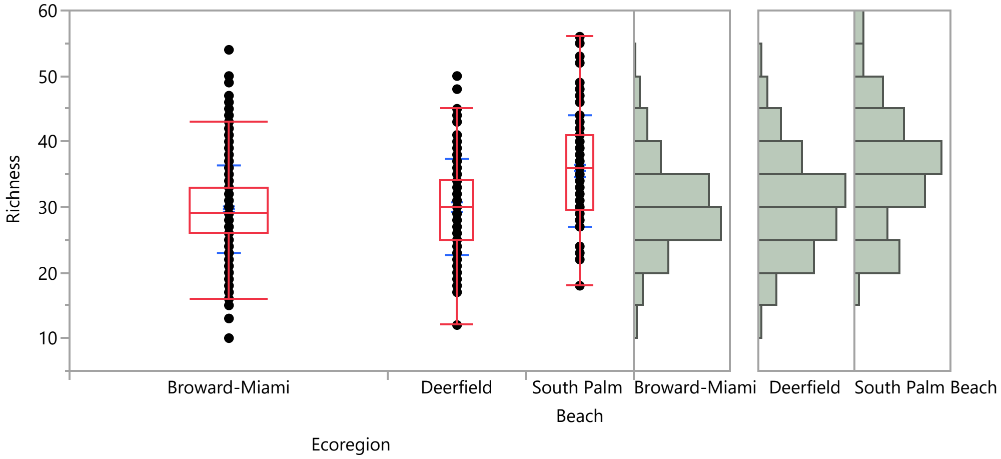

Means and Std Deviations

Level	Number	Mean	Std Dev	Std Err Mean	Lower 95%	Upper 95%
Broward-Miami	239	29.669456	6.6921775	0.432881	28.816689	30.522224
Deerfield	103	30	7.3431307	0.7235402	28.564862	31.435138
South Palm Beach	81	35.506173	8.5075312	0.9452812	33.625003	37.387342

Nonparametric Comparisons For Each Pair Using Wilcoxon Method

q*	Alpha
1.95996	0.05

Level	- Level	Score Mean Difference	Std Err Dif	Z	p-Value	Hodges-Lehmann	Lower CL	Upper CL	Difference Plot
South Palm Beach	Broward-Miami	65.49099	11.88191	5.511821	<.0001*	6.000000	4.00000	8.000000	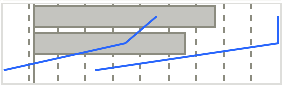
South Palm Beach	Deerfield	34.43797	7.90198	4.358142	<.0001*	5.000000	3.00000	8.000000	
Deerfield	Broward-Miami	4.32762	11.63861	0.371833	0.7100	0.000000	-1.00000	2.000000	

Where(:Depth == "Deep" & :Type 2 == "Hardbottom" & :Relief == "Low")

Fit Group

S5 Density and Richness between ecoregion by depth, type, relief

Fit Group

Oneway Analysis of TotalDensity By Ecoregion

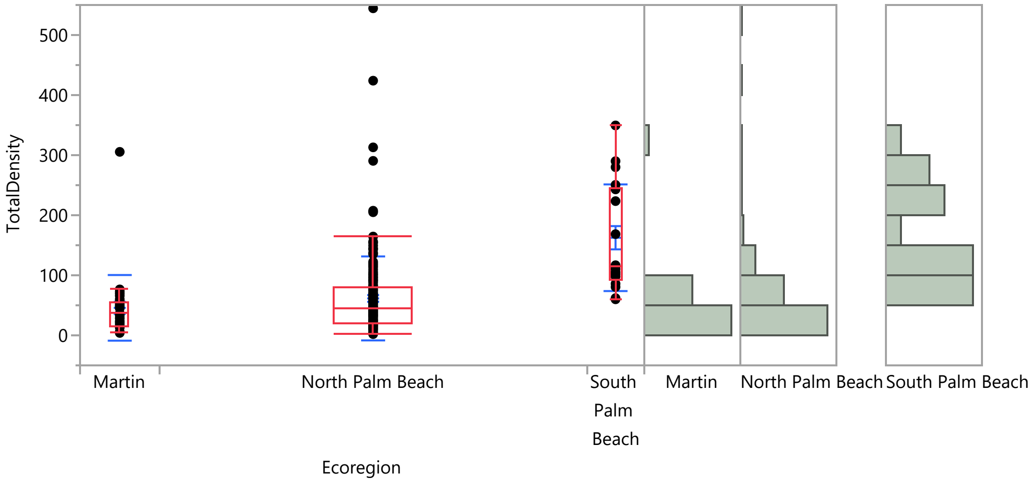

Means and Std Deviations

Level	Number	Mean	Std Dev	Std Err Mean	Lower 95%	Upper 95%
Martin	29	45.775862	54.648321	10.147939	24.988752	66.562972
North Palm Beach	156	61.471154	69.916707	5.5978166	50.413299	72.529009
South Palm Beach	21	162.5119	88.791908	19.375983	122.09431	202.9295

Nonparametric Comparisons For Each Pair Using Wilcoxon Method

q*	Alpha
1.95996	0.05

Level	- Level	Score Mean Difference	Std Err Dif	Z	p-Value	Hodges-Lehmann	Lower CL	Upper CL	Difference Plot
South Palm Beach	North Palm Beach	67.10440	11.90987	5.634351	<.0001*	83.25000	58.5000	129.5000	
South Palm Beach	Martin	22.61905	4.17662	5.415640	<.0001*	89.50000	61.0000	174.5000	
North Palm Beach	Martin	15.53935	10.82823	1.435077	0.1513	9.00000	-3.0000	23.0000	

S5 Density and Richness between ecoregion by depth, type, relief

Fit Group

Oneway Analysis of Richness By Ecoregion

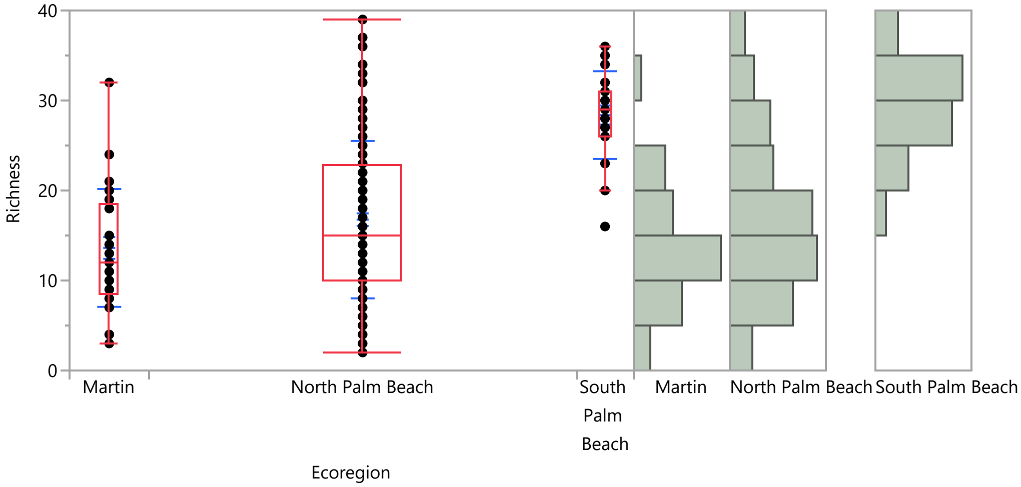

Means and Std Deviations

Level	Number	Mean	Std Dev	Std Err Mean	Lower 95%	Upper 95%
Martin	29	13.62069	6.5487937	1.2160805	11.129662	16.111718
North Palm Beach	156	16.762821	8.7459574	0.700237	15.379581	18.14606
South Palm Beach	21	28.380952	4.8731529	1.0634091	26.16272	30.599185

Nonparametric Comparisons For Each Pair Using Wilcoxon Method

q*	Alpha
1.95996	0.05

Level	- Level	Score Mean Difference	Std Err Dif	Z	p-Value	Hodges-Lehmann	Lower CL	Upper CL	Difference Plot
South Palm Beach	North Palm Beach	63.53846	11.90194	5.338498	<.0001*	13.00000	9.00000	16.00000	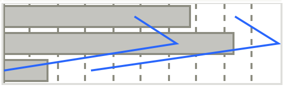
South Palm Beach	Martin	22.24959	4.17260	5.332305	<.0001*	16.00000	12.00000	19.00000	
North Palm Beach	Martin	19.24016	10.81934	1.778312	0.0754	3.00000	0.00000	6.00000	

Where(:Depth == "Deep" & :Type 2 == "Hardbottom" & :Relief == "High")

Fit Group

S5 Density and Richness between ecoregion by depth, type, relief

Fit Group

Oneway Analysis of TotalDensity By Ecoregion

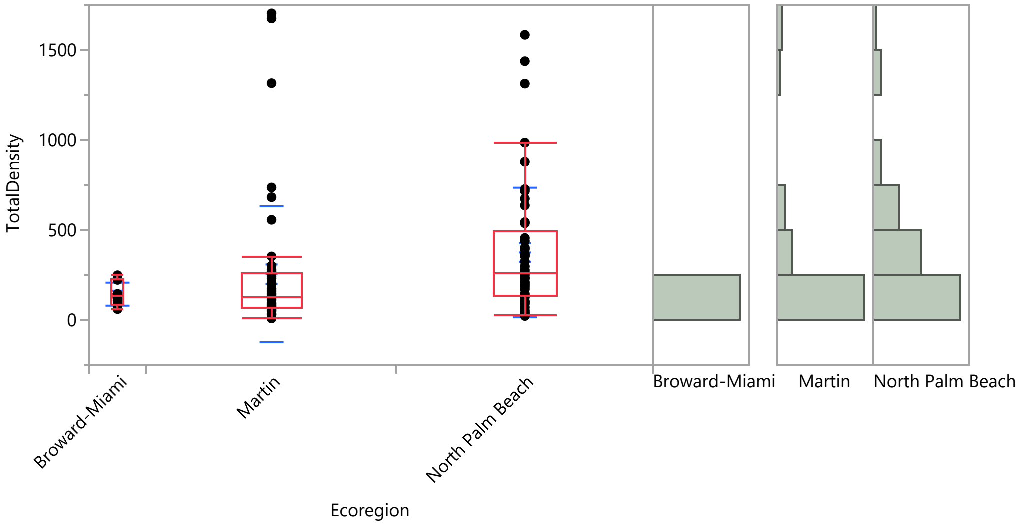

Means and Std Deviations

Level	Number	Mean	Std Dev	Std Err Mean	Lower 95%	Upper 95%
Broward-Miami	11	142.31818	63.919978	19.272599	99.376156	185.26021
Martin	48	252.80208	377.84022	54.536538	143.08872	362.51545
North Palm Beach	49	373.92347	360.26943	51.467062	270.44201	477.40493

Nonparametric Comparisons For Each Pair Using Wilcoxon Method

q*	Alpha
1.95996	0.05

Level	- Level	Score Mean Difference	Std Err Dif	Z	p-Value	Hodges-Lehmann	Lower CL	Upper CL	Difference Plot
North Palm Beach	Martin	16.9090	5.715761	2.95831	0.0031*	105.000	33.0000	183.5000	
North Palm Beach	Broward-Miami	14.0260	5.826733	2.40718	0.0161*	123.500	16.0000	270.5000	
Martin	Broward-Miami	-0.8939	5.741262	-0.15570	0.8763	-4.250	-58.0000	74.5000	

S5 Density and Richness between ecoregion by depth, type, relief

Fit Group

Oneway Analysis of Richness By Ecoregion

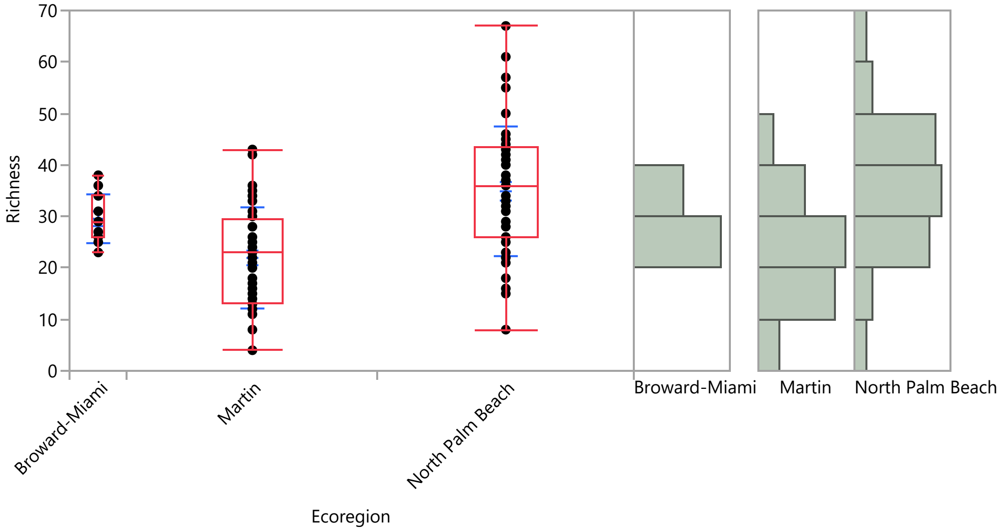

Means and Std Deviations

Level	Number	Mean	Std Dev	Std Err Mean	Lower 95%	Upper 95%
Broward-Miami	11	29.545455	4.7405408	1.4293268	26.360716	32.730193
Martin	48	21.9375	9.8248626	1.4180968	19.084657	24.790343
North Palm Beach	49	34.877551	12.594034	1.7991477	31.260122	38.49498

Nonparametric Comparisons For Each Pair Using Wilcoxon Method

q*	Alpha
1.95996	0.05

Level	- Level	Score Mean Difference	Std Err Dif	Z	p-Value	Hodges-Lehmann	Lower CL	Upper CL	Difference Plot
North Palm Beach	Martin	28.7247	5.712453	5.02844	<.0001*	13.0000	8.0000	18.0000	
North Palm Beach	Broward-Miami	9.1837	5.821226	1.57762	0.1147	5.0000	-1.0000	12.0000	
Martin	Broward-Miami	-15.6998	5.733791	-2.73812	0.0062*	-8.0000	-14.0000	-2.0000	

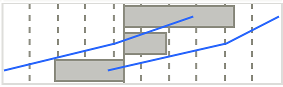

Where(:Depth == "Shallow" & :Type 2 == "Coral Reef" & :Relief == "Low")

Fit Group

Oneway Analysis of TotalDensity By Ecoregion

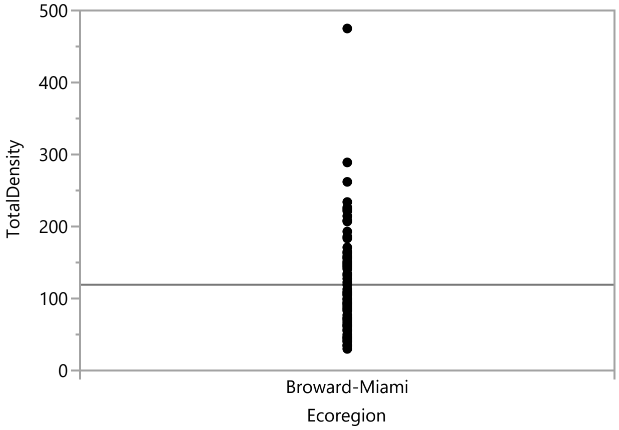

Oneway Analysis of Richness By Ecoregion

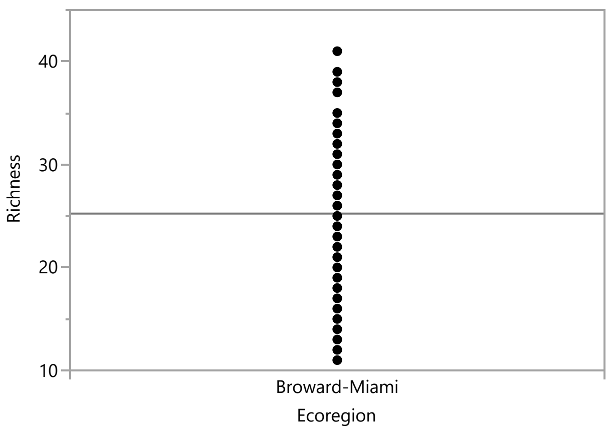

Where(:Depth == "Shallow" & :Type 2 == "Coral Reef" & :Relief == "High")

S5 Density and Richness between ecoregion by depth, type, relief

Fit Group

Oneway Analysis of TotalDensity By Ecoregion

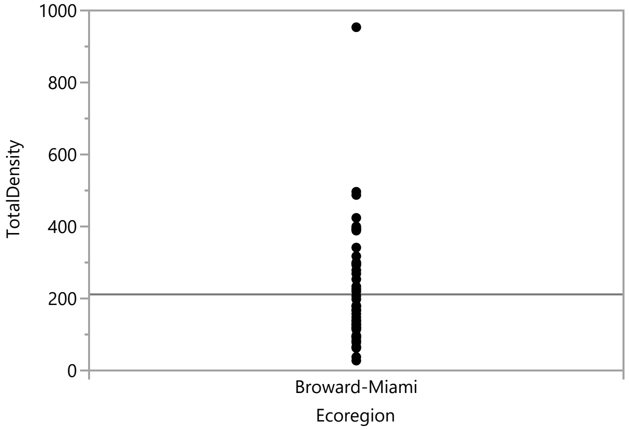

Oneway Analysis of Richness By Ecoregion

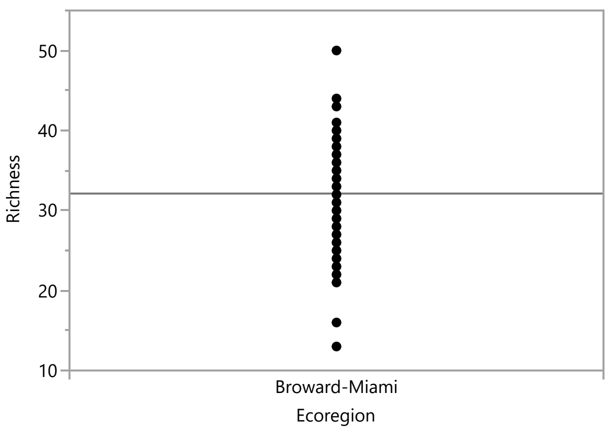

Where(:Depth == "Shallow" & :Type 2 == "Hardbottom" & :Relief == "Low")

Fit Group

Oneway Analysis of TotalDensity By Ecoregion

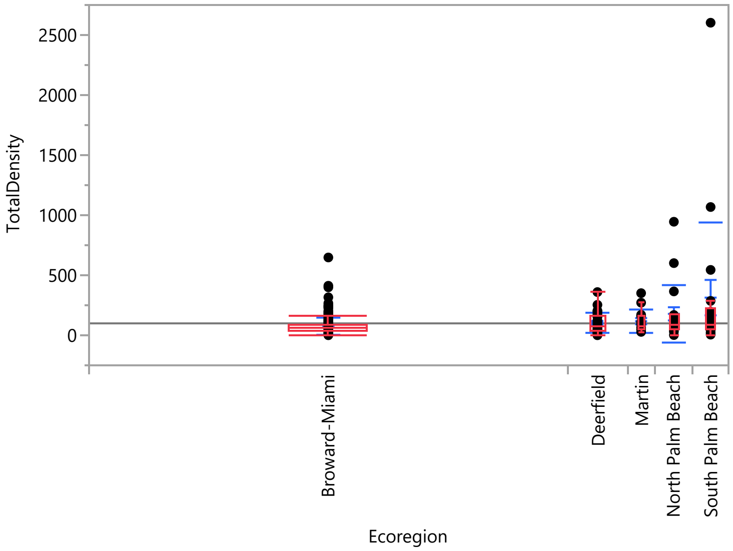

Means and Std Deviations

Level	Number	Mean	Std Dev	Std Err Mean	Lower 95%	Upper 95%
Broward-Miami	241	75.437178	71.523387	4.6072264	66.361414	84.512943
Deerfield	30	104.066	83.735814	15.287998	72.798533	135.33347
Martin	14	117.25	97.338179	26.014723	61.048608	173.45139
North Palm Beach	19	178.68421	239.21934	54.880681	63.384177	293.98424
South Palm Beach	18	313.61111	625.68862	147.47622	2.4634795	624.75874

Nonparametric Comparisons For Each Pair Using Wilcoxon Method

q*	Alpha
1.95996	0.05

Level	- Level	Score Mean Difference	Std Err Dif	Z	p-Value	Hodges-Lehmann	Lower CL	Upper CL	Difference Plot
South Palm Beach	Broward-Miami	42.27109	18.30375	2.309422	0.0209*	36.50000	4.5000	75.50000	
North Palm Beach	Broward-Miami	38.41232	17.91874	2.143695	0.0321*	26.50000	2.0000	57.50000	
Martin	Broward-Miami	35.18154	20.27618	1.735117	0.0827	24.50000	-3.0000	66.50000	
Deerfield	Broward-Miami	28.37441	15.17343	1.870007	0.0615	19.50000	-1.0000	45.25000	
South Palm Beach	Deerfield	2.80000	4.17388	0.670839	0.5023	18.75000	-28.0000	75.50000	
North Palm Beach	Deerfield	1.93421	4.18930	0.461702	0.6443	10.25000	-34.0000	59.50000	
South Palm Beach	Martin	1.71429	3.34284	0.512823	0.6081	12.75000	-48.0000	86.50000	

S5 Density and Richness between ecoregion by depth, type, relief

Fit Group

Oneway Analysis of TotalDensity By Ecoregion

Nonparametric Comparisons For Each Pair Using Wilcoxon Method

Level	- Level	Score Mean Difference	Std Err Dif	Z	p-Value	Hodges-Lehmann	Lower CL	Upper CL	Difference Plot
Martin	Deerfield	1.30952	4.15717	0.315004	0.7528	6.25000	-40.5000	61.00000	
South Palm Beach	North Palm Beach	0.70322	3.56033	0.197515	0.8434	8.25000	-49.5000	79.00000	
North Palm Beach	Martin	0.55827	3.40582	0.163917	0.8698	5.25000	-55.0000	60.00000	

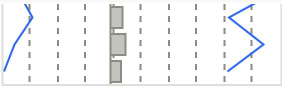

Oneway Analysis of Richness By Ecoregion

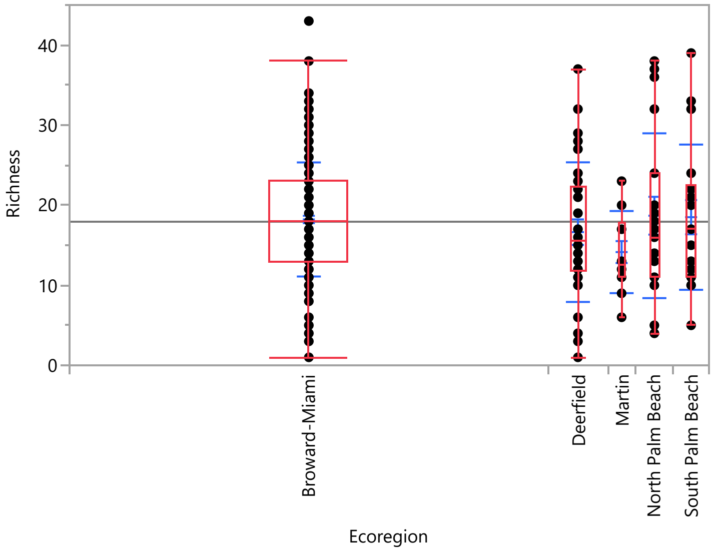

Means and Std Deviations

Level	Number	Mean	Std Dev	Std Err Mean	Lower 95%	Upper 95%
Broward-Miami	241	18.215768	7.1229617	0.4588303	17.311919	19.119616
Deerfield	30	16.633333	8.7197095	1.5919939	13.37734	19.889326
Martin	14	14.142857	5.1269596	1.3702376	11.182639	17.103075
North Palm Beach	19	18.684211	10.290517	2.3608065	13.72434	23.644081
South Palm Beach	18	18.5	9.0699893	2.137817	13.9896	23.0104

Nonparametric Comparisons For Each Pair Using Wilcoxon Method

q*	Alpha
1.95996	0.05

Level	- Level	Score Mean Difference	Std Err Dif	Z	p-Value	Hodges-Lehmann	Lower CL	Upper CL	Difference Plot
North Palm Beach	Martin	4.0940	3.39642	1.20538	0.2281	3.00000	-2.00000	8.000000	
South Palm Beach	Martin	3.9365	3.32995	1.18215	0.2371	3.50000	-1.00000	9.000000	
North Palm Beach	Deerfield	1.9342	4.18492	0.46219	0.6439	1.00000	-4.00000	7.000000	
South Palm Beach	Deerfield	1.6889	4.16753	0.40525	0.6853	1.00000	-4.00000	7.000000	
South Palm Beach	North Palm Beach	0.3246	3.55484	0.09130	0.9273	0.00000	-6.00000	6.000000	
Martin	Deerfield	-4.4000	4.14734	-1.06092	0.2887	-2.00000	-7.00000	2.000000	
South Palm Beach	Broward-Miami	-5.2540	18.28451	-0.28735	0.7738	-1.00000	-4.00000	4.000000	
North Palm Beach	Broward-Miami	-7.3815	17.89970	-0.41238	0.6801	-1.00000	-5.00000	3.000000	
Deerfield	Broward-Miami	-17.3358	15.15801	-1.14367	0.2528	-2.00000	-5.00000	1.000000	
Martin	Broward-Miami	-45.4601	20.25278	-2.24464	0.0248*	-4.00000	-8.00000	0.000000	

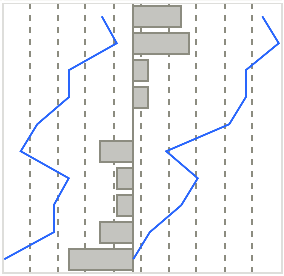

Where(:Depth == "Shallow" & :Type 2 == "Hardbottom" & :Relief == "High")

S5 Density and Richness between ecoregion by depth, type, relief

Fit Group

Oneway Analysis of TotalDensity By Ecoregion

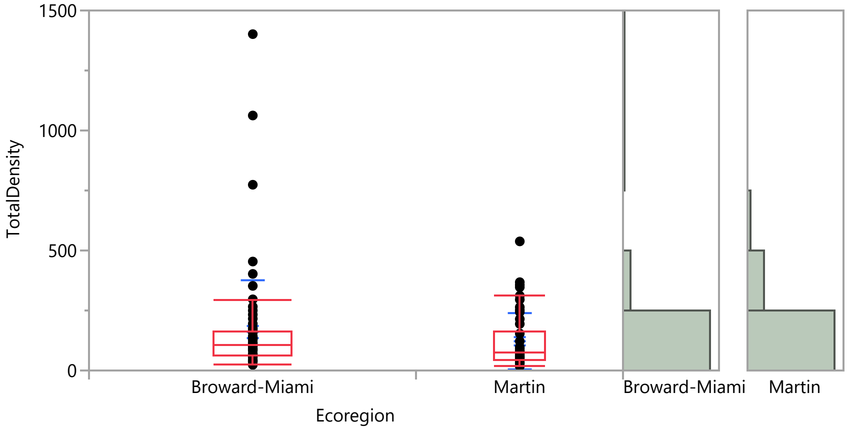

Means and Std Deviations

Level	Number	Mean	Std Dev	Std Err Mean	Lower 95%	Upper 95%
Broward-Miami	73	160.28082	215.74979	25.251603	109.94267	210.61897
Martin	46	121.88043	117.29645	17.294418	87.047688	156.71318

Wilcoxon / Kruskal-Wallis Tests (Rank Sums)

Level	Count	Score Sum	Expected Score	Score Mean	(Mean-Mean0)/Std0
Broward-Miami	73	4685.00	4380.00	64.1781	1.662
Martin	46	2455.00	2760.00	53.3696	-1.662

2-Sample Test, Normal Approximation

S	Z	Prob>|Z|
2455	-1.66179	0.0966

1-Way Test, ChiSquare Approximation

ChiSquare	DF	Prob>ChiSq
2.7706	1	0.0960

S5 Density and Richness between ecoregion by depth, type, relief

Fit Group

Oneway Analysis of Richness By Ecoregion

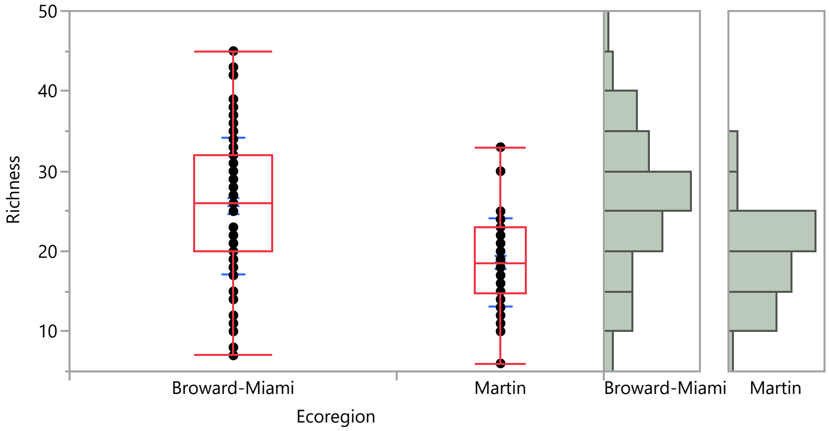

Quantiles

Level	Minimum	10%	25%	Median	75%	90%	Maximum
Broward-Miami	7	14	20	26	32	36.6	45
Martin	6	11.7	14.75	18.5	23	24.3	33

Means and Std Deviations

Level	Number	Mean	Std Dev	Std Err Mean	Lower 95%	Upper 95%
Broward-Miami	73	25.643836	8.5332192	0.9987378	23.652888	27.634783
Martin	46	18.608696	5.5074828	0.812034	16.973175	20.244216

Wilcoxon / Kruskal-Wallis Tests (Rank Sums)

Level	Count	Score Sum	Expected Score	Score Mean	(Mean-Mean0)/Std0
Broward-Miami	73	5231.50	4380.00	71.6644	4.649
Martin	46	1908.50	2760.00	41.4891	-4.649

2-Sample Test, Normal Approximation

S	Z	Prob>|Z|
1908.5	-4.64867	<.0001*

1-Way Test, ChiSquare Approximation

ChiSquare	DF	Prob>ChiSq
21.6355	1	<.0001*
