# Supplementary material for: Regional reef fish assemblage maps provide baseline biogeography for tropicalization monitoring
Source: Sci Rep. 2024 Apr 3;14:7893. doi: 10.1038/s41598-024-58185-6 (PMC10991435; doi:10.1038/s41598-024-58185-6)
Supplement: Supplementary file 6 — Supplementary Information 6. [file 41598_2024_58185_MOESM6_ESM.pdf]

| | | | Shallow assemblage percent occurrence | | | | | | | | |
|---|---|---|---|---|---|---|---|---|---|---|---|
| | | | Martin | | North Palm Beach | South Palm Beach | Deerfield | Broward-Miami | | | |
| | | | Hardbottom | | Hardbottom | Hardbottom | Hardbottom | Reef | | Hardbottom | |
| | | | High | Low | Low | Low | Low | High | Low | High | Low |
| Scientific name | Common Name | Thermal Affinity (Fishbase.org) | SMNHBH | SMNHBL | SNPHBL | SSPHBL | SDFHBL | SBMCRH | SBMCRL | SBMHBH | SBMHBL |
| Halichoeres bivittatus | slippery dick | Tropical; 37°N - 26°S | 87.5% | 100.0% | 90.0% | 94.4% | 59.4% | 89.5% | 84.5% | 90.9% | 86.3% |
| Acanthurus tractus | ocean surgeon | Tropical; 21°C - 25°C; 44°N - 7°N | 47.9% | 43.8% | 70.0% | 94.4% | 65.6% | 92.1% | 89.7% | 89.8% | 82.0% |
| Canthigaster rostrata | sharpnose puffer | Tropical; 34°N - 8°N | 39.6% | 37.5% | 75.0% | 77.8% | 56.3% | 90.8% | 82.8% | 93.2% | 85.3% |
| Stegastes xanthurus | cocoa damselfish | Tropical; 6°S - 30°S | 83.3% | 87.5% | 70.0% | 61.1% | 46.9% | 78.9% | 72.4% | 61.4% | 49.6% |
| Thalassoma bifasciatum | bluehead | Tropical; 23°C - 26°C | 47.9% | 31.3% | 55.0% | 66.7% | 50.0% | 96.1% | 94.8% | 93.2% | 73.0% |
| Acanthurus chirurgus | doctorfish | Subtropical; 22°C - 25°C ; 43°N - 30°S | 87.5% | 56.3% | 65.0% | 72.2% | 37.5% | 57.9% | 69.0% | 80.7% | 58.3% |
| Stegastes partitus | bicolor damselfish | Tropical; 33°N - 5°S, | 16.7% | 6.3% | 85.0% | 55.6% | 25.0% | 98.7% | 96.6% | 67.0% | 46.8% |
| Haemulon plumierii | white grunt | Subtropical; 39°N - 23°S | 62.5% | 62.5% | 15.0% | 16.7% | 40.6% | 69.7% | 50.0% | 83.0% | 66.9% |
| Anisotremus virginicus | porkfish | Subtropical; 32°N - 33°S | 95.8% | 43.8% | 80.0% | 44.4% | 37.5% | 59.2% | 36.2% | 47.7% | 21.2% |
| Halichoeres maculipinna | clown wrasse | Tropical; 36°N - 25°S | 6.3% | 37.5% | 10.0% | 77.8% | 46.9% | 77.6% | 81.0% | 67.0% | 59.0% |
| Sparisoma aurofrenatum | redband parrotfish | Subtropical; 33°N - 8°N | 2.1% | | 40.0% | 33.3% | 18.8% | 100.0% | 83.6% | 94.3% | 65.1% |
| Pseudupeneus maculatus | spotted goatfish | Subtropical; 40°N - 30°S | 29.2% | 18.8% | 35.0% | 44.4% | 62.5% | 59.2% | 61.2% | 62.5% | 63.3% |
| Stegastes leucostictus | beaugregory | Tropical; 33°N - 35°S | 85.4% | 50.0% | 40.0% | 38.9% | 18.8% | 46.1% | 33.6% | 43.2% | 23.4% |
| Scarus iseri | striped parrotfish | Subtropical; 23°C - 26°C | 4.2% | 12.5% | 35.0% | 33.3% | 28.1% | 81.6% | 68.1% | 38.6% | 30.9% |
| Acanthurus coeruleus | blue tang | Tropical; 43°N - 27°S | 12.5% | 6.3% | 25.0% | 11.1% | 15.6% | 86.8% | 58.6% | 76.1% | 38.5% |
| Abudefduf saxatilis | sergeant major | Subtropical; 41°N - 37°S | 25.0% | 25.0% | 45.0% | 88.9% | 34.4% | 43.4% | 17.2% | 35.2% | 11.2% |
| Haemulon flavolineatum | French grunt | Subtropical; 34°N - 34°S | 37.5% | 18.8% | 40.0% | 27.8% | 21.9% | 73.7% | 27.6% | 56.8% | 20.9% |
| Haemulon sp. | grunt species | Subtropical; 22°C - 25°C | 47.9% | 31.3% | 45.0% | 61.1% | 43.8% | 11.8% | 11.2% | 29.5% | 38.8% |
| Ocyurus chrysurus | yellowtail snapper | Subtropical; 42°N - 26°S | 29.2% | 18.8% | 15.0% | | 18.8% | 73.7% | 50.9% | 51.1% | 55.4% |
| Haemulon aurolineatum | tomtate | Subtropical; 43°N - 33°S | 56.3% | 56.3% | 35.0% | 38.9% | 21.9% | 17.1% | 5.2% | 37.5% | 17.6% |
| Halichoeres garnoti | yellowhead wrasse | Tropical; 23°C - 27°C | | | 5.0% | 5.6% | 18.8% | 98.7% | 81.9% | 46.6% | 21.9% |
| Caranx ruber | bar jack | Subtropical; 35°N - 33°N | 16.7% | 18.8% | 25.0% | 44.4% | 53.1% | 19.7% | 31.0% | 31.8% | 22.7% |
| Parablennius marmoreus | seaweed blenny | Subtropical; 36°N - 27°S | 54.2% | 31.3% | 40.0% | 66.7% | 25.0% | 3.9% | 8.6% | 9.1% | 24.5% |
| Pomacanthus paru | French angelfish | Subtropical; 33°N - 30°S | 31.3% | 6.3% | 40.0% | 27.8% | 21.9% | 32.9% | 36.2% | 30.7% | 22.7% |
| Haemulon sciurus | bluestriped grunt | Subtropical; 33°N - 19°S | 6.3% | 12.5% | 25.0% | 22.2% | 34.4% | 48.7% | 27.6% | 52.3% | 19.1% |
| Sparisoma viride | stoplight parrotfish | Subtropical; 34°N - 21°S | | | 20.0% | | 3.1% | 78.9% | 51.7% | 67.0% | 26.6% |
| Pomacanthus arcuatus | gray angelfish | Tropical; 22°C - 28°C | 12.5% | 6.3% | 5.0% | 5.6% | 3.1% | 60.5% | 51.7% | 52.3% | 46.4% |
| Pareques acuminatus | high-hat | Tropical; 38°N - 33°S | 31.3% | 43.8% | 20.0% | 38.9% | 40.6% | 14.5% | 7.8% | 22.7% | 21.2% |
| Balistes capriscus | gray triggerfish | Tropical; 58°N - 37°S | 10.4% | | 25.0% | 16.7% | 50.0% | 25.0% | 31.9% | 27.3% | 39.6% |
| Lutjanus synagris | lane snapper | Subtropical; 38°N - 38°S | 31.3% | 68.8% | 20.0% | 66.7% | 12.5% | 2.6% | 3.4% | 6.8% | 6.1% |
| Sparisoma atomarium | greenblotch parrotfish | Tropical; 32°N | | | 20.0% | 11.1% | 34.4% | 40.8% | 35.3% | 38.6% | 25.5% |
| Bodianus rufus | Spanish hogfish | Tropical; 22°C - 28°C ); 33°N - 26°S | 12.5% | | 20.0% | 5.6% | 3.1% | 64.5% | 44.8% | 28.4% | 10.1% |
| Malacoctenus triangulatus | saddled blenny | Subtropical; 32°N - 26°S | 35.4% | 12.5% | 10.0% | 27.8% | 12.5% | 14.5% | 19.0% | 33.0% | 24.1% |
| Diplodus holbrookii | spottail pinfish | Subtropical; 40°N - 20°N | 81.3% | 43.8% | 10.0% | 22.2% | | | 2.6% | 11.4% | 3.6% |
| Chaetodon sedentarius | reef butterflyfish | Subtropical; 36°N - 25°S | 8.3% | 12.5% | 5.0% | | 3.1% | 60.5% | 50.9% | 17.0% | 10.8% |
| Serranus tigrinus | harlequin bass | Tropical; 33°N - 7°N | 4.2% | | 10.0% | | | 75.0% | 54.3% | 12.5% | 4.7% |
| Lutjanus griseus | gray snapper | Subtropical; 42°N - 9°N | 39.6% | 6.3% | 15.0% | 5.6% | 28.1% | 14.5% | 14.7% | 22.7% | 11.2% |
| Stegastes adustus | dusky damselfish | Tropical; 32°N - 8°N | 12.5% | 18.8% | 10.0% | 16.7% | 15.6% | 34.2% | 12.9% | 20.5% | 9.4% |
| Scarus taeniopterus | princess parrotfish | Tropical; 33°N - 34°S | | | | | | 60.5% | 43.1% | 30.7% | 13.7% |
| Caranx crysos | blue runner | Subtropical; 46°N - 26°S | 20.8% | 25.0% | 15.0% | 27.8% | 15.6% | 9.2% | 7.8% | 12.5% | 10.8% |
| Coryphopterus personatus | masked goby | Tropical; 32°N - | 8.3% | 6.3% | 5.0% | | 3.1% | 60.5% | 19.8% | 33.0% | 5.8% |
| Lachnolaimus maximus | hogfish | Subtropical; 46°N - 4°N | 4.2% | | | | 3.1% | 40.8% | 35.3% | 22.7% | 30.2% |
| Hypoplectrus unicolor | butter hamlet | Tropical; 32°N - 6°N | 4.2% | 6.3% | | | 9.4% | 57.9% | 35.3% | 15.9% | 5.0% |
| Cantherhines pullus | orangespotted filefish | Subtropical; 42°N - 26°S | 16.7% | 6.3% | 15.0% | | 31.3% | 25.0% | 13.8% | 14.8% | 7.2% |
| Chaetodon ocellatus | spotfin butterflyfish | Tropical; 45°N - 8°N | 12.5% | 12.5% | 20.0% | | | 36.8% | 18.1% | 18.2% | 10.8% |
| Coryphopterus glaucofraenum | bridled goby | Tropical; 37°N - 33°S | 14.6% | | 10.0% | 5.6% | | 25.0% | 31.0% | 14.8% | 25.5% |
| Xyrichtys splendens | green razorfish | Subtropical; 32°N - 33°S | 14.6% | 18.8% | 15.0% | 44.4% | 6.3% | 2.6% | 9.5% | 2.3% | 12.6% |
| Lutjanus analis | mutton snapper | Tropical; 20°C - 28°C; 42°N - 28°S | 16.7% | 12.5% | 20.0% | | 18.8% | 14.5% | 12.9% | 15.9% | 10.4% |
| Sparisoma rubripinne | yellowtail parrotfish | Tropical; 42°N - 7°N | 33.3% | 12.5% | 5.0% | 5.6% | 9.4% | 10.5% | 11.2% | 19.3% | 13.7% |
| Labrisomus nuchipinnis | hairy blenny | Tropical; 34°N - 34°S | 54.2% | 50.0% | 5.0% | 5.6% | | | | 3.4% | 0.7% |
| Halichoeres poeyi | blackear wrasse | Tropical; 30°N - 33°S | | 12.5% | 20.0% | 16.7% | 6.3% | 11.8% | 10.3% | 25.0% | 12.6% |
| Elacatinus oceanops | neon goby | Subtropical; 21°C - 28°C | 4.2% | | | | | 31.6% | 23.3% | 34.1% | 19.8% |
| Halichoeres radiatus | puddingwife | Tropical; 23°C - 27°C; 37°N - 19°S | 10.4% | 6.3% | 20.0% | 27.8% | 6.3% | 11.8% | 2.6% | 20.5% | 5.4% |
| Haemulon carbonarium | caesar grunt | Subtropical; 33°N - 34°S | 12.5% | | 10.0% | 11.1% | 15.6% | 17.1% | 6.0% | 30.7% | 4.7% |
| Holacanthus tricolor | rock beauty | Tropical; 37°N - 29°S | | | 25.0% | | 6.3% | 39.5% | 24.1% | 8.0% | 3.6% |
| Chaetodon capistratus | foureye butterflyfish | Subtropical; 21°C - 28°C | 2.1% | | | | | 57.9% | 31.9% | 10.2% | 3.6% |
| Diodon holocanthus | balloonfish | Subtropical; 37°N - 39°S | 2.1% | | 10.0% | | 31.3% | 15.8% | 12.9% | 19.3% | 13.0% |
| Cryptotomus roseus | bluelip parrotfish | Tropical; 32°N - 33°S | | | 25.0% | | 6.3% | 17.1% | 21.6% | 12.5% | 19.1% |
| Cephalopholis cruentata | graysby | Subtropical; 36°N - 6°N | | | 10.0% | | | 55.3% | 28.4% | 5.7% | 1.1% |
| Holacanthus ciliaris | queen angelfish | Subtropical; 33°N - 35°S | 4.2% | | 15.0% | | 3.1% | 28.9% | 18.1% | 15.9% | 6.8% |
| Haemulon parra | sailors choice | Subtropical; 31°N - 30°S | 33.3% | 6.3% | 15.0% | 5.6% | 9.4% | 1.3% | 5.2% | 8.0% | 3.6% |
| Sphoeroides spengleri | bandtail puffer | Subtropical; 43°N - 30°S | 2.1% | 6.3% | 5.0% | | 25.0% | 5.3% | 4.3% | 11.4% | 20.9% |
| Kyphosus sectatrix | Bermuda chub | Subtropical; 46°N - 29°S | 14.6% | 6.3% | 5.0% | 11.1% | 3.1% | 9.2% | 3.4% | 20.5% | 4.7% |
| Calamus calamus | saucereye porgy | Subtropical; 37°N - 33°S | 20.8% | 6.3% | | | 12.5% | 5.3% | 19.0% | 2.3% | 8.6% |
| Acanthostracion quadricornis | scrawled cowfish | Subtropical; 43°N - 39°S | | | 20.0% | | 37.5% | 7.9% | 5.2% | 1.1% | 2.2% |
| Haemulon melanurum | cottonwick | Subtropical; 34°N - 12°S | | 6.3% | 5.0% | 22.2% | 9.4% | 2.6% | 6.0% | 12.5% | 8.6% |
| Sparisoma chrysopterum | redtail parrotfish | Tropical; 28°N - 4°N | | | | 11.1% | 15.6% | 9.2% | 6.9% | 15.9% | 13.3% |
| Diplodus argenteus | silver porgy | Subtropical; 20°S - 35°S | 8.3% | 12.5% | 25.0% | 11.1% | 9.4% | 2.6% | | | 2.2% |
| Serranus baldwini | lantern bass | Tropical; 33°N - 36°S | | | 10.0% | | | 13.2% | 17.2% | 11.4% | 15.1% |
| Sparisoma radians | bucktooth parrotfish | Tropical; 33°N - 30°S | | | 5.0% | | 12.5% | 14.5% | 6.0% | 19.3% | 6.8% |
| Anisotremus surinamensis | black margate | Subtropical; 31°N - 35°S | 29.2% | 6.3% | | | 6.3% | 7.9% | 2.6% | 9.1% | 1.8% |
| Holacanthus bermudensis | blue angelfish | Subtropical; 35°N - 18°N | 12.5% | | | | | 21.1% | 13.8% | 10.2% | 4.0% |
| Scorpaena plumieri | spotted scorpionfish | Subtropical; 43°N - 30°S | 12.5% | 12.5% | 10.0% | | 9.4% | | 3.4% | 9.1% | 3.6% |
| Malacoctenus macropus | rosy blenny | Subtropical; 32°N - | 6.3% | 18.8% | 10.0% | | 3.1% | | 4.3% | 6.8% | 11.2% |
| Azurina multilineata | brown chromis | Subtropical; 33°N - 31°S | | | 10.0% | 11.1% | | 26.3% | 4.3% | 6.8% | 0.4% |
| Urobatis jamaicensis | yellow stingray | Tropical; 37°N - 7°N | | | 5.0% | | 12.5% | 10.5% | 6.9% | 10.2% | 11.5% |
| Gnatholepis thompsoni | goldspot goby | Tropical; 43°N - 2°S | | | 10.0% | 5.6% | | 21.1% | 4.3% | 8.0% | 6.8% |
| Gerres cinereus | yellowfin mojarra | Subtropical; 33°N - 23°S | 8.3% | | 15.0% | 11.1% | 12.5% | | | 2.3% | 4.3% |
| Caranx bartholomaei | yellow jack | Subtropical; 42°N - 25°S | 2.1% | 12.5% | 10.0% | 5.6% | 9.4% | 1.3% | | 9.1% | 2.9% |
| Stephanolepis hispidus | planehead filefish | Subtropical; 46°N - 37°S | | | | | 40.6% | | 1.7% | 5.7% | 3.6% |
| Epinephelus morio | red grouper | Subtropical; 35°N - 27°S | | | 5.0% | | 3.1% | 9.2% | 13.8% | 10.2% | 7.9% |
| Lactophrys triqueter | smooth trunkfish | Subtropical; 44°N - 35°S | | | | | 15.6% | 10.5% | 12.9% | 2.3% | 7.9% |
| Stegastes planifrons | threespot damselfish | Tropical | 2.1% | | | | | 13.2% | 11.2% | 17.0% | 3.6% |
| Aluterus scriptus | scrawled filefish | Subtropical; 46°N - 30°S | 2.1% | | | | 15.6% | 11.8% | 7.8% | 6.8% | 2.2% |
| Ptereleotris calliura | blue dartfish | Tropical | | | 15.0% | | | 6.6% | 11.2% | 3.4% | 9.4% |
| Scarus guacamaia | rainbow parrotfish | Subtropical; 33°N - 41°S | 8.3% | 6.3% | | | 6.3% | 7.9% | 9.5% | 4.5% | 2.5% |
| Chromis scotti | purple reeffish | Tropical; 35°N - 23°S | | | | 27.8% | | 13.2% | 2.6% | 1.1% | |
| Haemulon striatum | striped grunt | Subtropical; 35°N - 33°S | | | 10.0% | | 15.6% | 3.9% | | 11.4% | 1.4% |
| Azurina cyanea | blue chromis | Tropical; 34°N - 7°N | | | 5.0% | | | 25.0% | 5.2% | 5.7% | 0.7% |
| Chaetodon striatus | banded butterflyfish | Tropical; 41°N - 29°S | 2.1% | | | | 9.4% | 13.2% | 7.8% | 4.5% | 3.2% |
| Aulostomus maculatus | Atlantic trumpetfish | Subtropical; 33°N - 0°N | | | 5.0% | | 12.5% | 2.6% | 0.9% | 13.6% | 4.3% |
| Serranus subligarius | belted sandfish | #N/A | 27.1% | 6.3% | | 5.6% | | | | | |
| Pterois volitans | red lionfish | Tropical; 22°C - 28°C; 43°N - 40°S | | | 5.0% | 11.1% | | 11.8% | 6.0% | 3.4% | 1.1% |
| Calamus penna | sheepshead porgy | Tropical; 30°N - 33°S | | 12.5% | | | 15.6% | 2.6% | 1.7% | 3.4% | 2.2% |
| Diplectrum formosum | sand perch | Subtropical; 42°N - 35°S | 2.1% | | 5.0% | 5.6% | | 2.6% | 6.9% | 1.1% | 13.7% |
| Serranus tabacarius | tobaccofish | Subtropical; 34°N - 10°S | | | | | 9.4% | 3.9% | 12.9% | 3.4% | 4.0% |
| Coryphopterus dicrus | colon goby | Subtropical | | | 5.0% | | | 9.2% | 5.2% | 10.2% | 3.6% |
| Microspathodon chrysurus | yellowtail damselfish | Subtropical; 33°N - 25°S | 2.1% | | | | | 13.2% | 3.4% | 12.5% | 1.8% |
| Eques punctatus | spotted drum | Tropical; 36°N - 24°S | | 12.5% | 5.0% | | 6.3% | | | 1.1% | 7.2% |
| Haemulon macrostoma | Spanish grunt | Tropical; 32°N - 34°S | 6.3% | | | | | 10.5% | 0.9% | 12.5% | 0.4% |
| Holocentrus adscensionis | squirrelfish | Subtropical; 37°N - 25°S | | | | | | 9.2% | 4.3% | 14.8% | 2.2% |
| Monacanthus tuckeri | slender filefish | Subtropical; 37°N - 10°N | | | 10.0% | 5.6% | | | 6.9% | 4.5% | 2.5% |
| Chaetodipterus faber | Atlantic spadefish | Subtropical; 43°N - 34°S | 4.2% | 6.3% | | | | 6.6% | | 10.2% | 2.2% |
| Seriola rivoliana | almaco jack | Subtropical; 43°N - 38°S | 4.2% | 6.3% | | | 9.4% | 3.9% | 5.2% | | 0.4% |
| Odontoscion dentex | reef croaker | Tropical; 22°N - 25°S | 29.2% | | | | | | | | |
| Calamus proridens | littlehead porgy | Subtropical; 30°N - 18°N | 2.1% | | 10.0% | 5.6% | 3.1% | 1.3% | 3.4% | 1.1% | 1.1% |
| Scarus vetula | queen parrotfish | Tropical; 33°N - 8°N | | | | | | 6.6% | 6.0% | 11.4% | 3.2% |
| Eques lanceolatus | jackknife-fish | Tropical; 37°N - 34°S | 4.2% | 18.8% | | | | | | | 1.4% |
| Chloroscombrus chrysurus | Atlantic bumper | Subtropical; 42°N - 41°S | | 6.3% | | 11.1% | | 1.3% | 0.9% | 3.4% | 1.1% |
| Hypoplectrus gemma | blue hamlet | Tropical | | | | | | 14.5% | 7.8% | 1.1% | 0.4% |
| Stegastes diencaeus | longfin damselfish | Tropical | | | | | | 6.6% | 3.4% | 9.1% | 4.0% |
| Ptereleotris helenae | hovering dartfish | Tropical | | | 5.0% | | | 6.6% | 5.2% | 1.1% | 3.6% |
| Bodianus parrae | creole wrasse | Tropical; 34°N - 8°N | | | 5.0% | | | 10.5% | 2.6% | 2.3% | 0.7% |
| Brachygenys chrysargyrea | smallmouth grunt | Subtropical; 32°N - 34°S | 2.1% | | | | | | 1.7% | 13.6% | 2.2% |
| Gymnothorax moringa | spotted moray | Subtropical; 37°N - 26°S | 6.3% | | 5.0% | 5.6% | | 2.6% | | | |
| Xyrichtys martinicensis | rosy razorfish | Subtropical | | | 5.0% | 5.6% | | | 3.4% | | 5.0% |
| Megalops atlanticus | tarpon | Subtropical; 10°C - 40°C; 49°N - 44°S | | | 10.0% | | 6.3% | | | 1.1% | 1.1% |
| Scomberomorus regalis | cero | Tropical; 41°N - 24°S | | | | | | 6.6% | 4.3% | 5.7% | 1.8% |
| Ginglymostoma cirratum | nurse shark | Subtropical; 44°N - 35°S | 2.1% | | | | 3.1% | | 1.7% | 5.7% | 5.8% |
| Nicholsina usta | emerald parrotfish | Tropical; 41°N - 30°S | 2.1% | 12.5% | | | 3.1% | | | | 0.4% |
| Acanthostracion polygonium | honeycomb cowfish | Tropical; 42°N - 35°S | | | | 5.6% | 6.3% | 2.6% | 1.7% | | 1.1% |
| Lutjanus apodus | schoolmaster | Subtropical; 42°N - 19°S | 4.2% | | | | | 5.3% | 0.9% | 6.8% | |
| Diodon hystrix | porcupinefish | Subtropical; 35°N - 31°S | | | 5.0% | | 6.3% | 2.6% | 1.7% | 1.1% | 0.4% |
| Fistularia tabacaria | bluespotted cornetfish | Tropical; 48°N - 29°S | | | 5.0% | | 6.3% | 1.3% | 1.7% | | 2.2% |
| Xyrichtys novacula | pearly razorfish | Subtropical | | 6.3% | 5.0% | | 3.1% | | | | 1.4% |
| Halichoeres cyanocephalus | yellowcheek wrasse | Tropical; 30°N - 33°S | | | 5.0% | 5.6% | | 1.3% | 3.4% | | 0.4% |
| Pempheris schomburgkii | glassy sweeper | Tropical | | | | 11.1% | | 3.9% | | | 0.4% |
| Lutjanus mahogoni | mahogany snapper | Subtropical; 36°N - 9°N | 2.1% | | | | 6.3% | 3.9% | 2.6% | | 0.4% |
| Hypoplectrus puella | barred hamlet | Tropical | | | 5.0% | | | 3.9% | 1.7% | 3.4% | 1.1% |
| Apogon maculatus | flamefish | Tropical; 46°N - 7°N | | | | 5.6% | 3.1% | 1.3% | 1.7% | 2.3% | 1.1% |
| Decapterus punctatus | round scad | Subtropical; 45°N - 35°S | 8.3% | | | | 6.3% | | | | |
| Holocanthus sp. | townsend angelfish | | | | | | | 9.2% | 3.4% | 1.1% | |
| Pareques umbrosus | cubbyu | Subtropical; 37°N - 4°N | 12.5% | | | | | | | 1.1% | |
| Apogon pseudomaculatus | twospot cardinalfish | Tropical; 43°N - 34°S | | | 5.0% | 5.6% | | | 1.7% | | 1.1% |
| Opistognathus aurifrons | yellowhead jawfish | Tropical; 22°C - 28°C; 32°N - 23°S | | | | | | 1.3% | 9.5% | | 2.5% |
| Malacanthus plumieri | sand tilefish | Subtropical; 34°N - 8°S | | | | 11.1% | | | 1.7% | | 0.4% |
| Chromis insolata | sunshinefish | Tropical; 37°N - 7°N | | | 5.0% | | 3.1% | 2.6% | 1.7% | | 0.4% |
| Serranus tortugarum | chalk bass | Tropical; 32°N - 7°N | | | | | | 3.9% | 7.8% | | 1.1% |
| Calamus bajonado | jolthead porgy | Subtropical; 42°N - 18°S | 2.1% | | | 5.6% | 3.1% | | 0.9% | | 0.7% |
| Scarus coeruleus | blue parrotfish | Tropical; 39°N - 24°S | | | | 5.6% | 3.1% | | 1.7% | 1.1% | 0.7% |
| Cephalopholis fulva | coney | Subtropical; 36°N - 28°S | | | 5.0% | | | 2.6% | 3.4% | 1.1% | |
| Emblemaria pandionis | sailfin blenny | Tropical; 32°N - 4°N | | | | | 6.3% | | 3.4% | | 2.5% |
| Euthynnus alletteratus | little tunny | Tropical; 56°N - 30°S | | 6.3% | 5.0% | | | | | | |
| Sphyraena barracuda | great barracuda | Subtropical; 42°N - 35°S | 4.2% | 6.3% | | | | | | | 0.7% |
| Gymnothorax vicinus | purplemouth moray | Subtropical | 4.2% | | 5.0% | | | | 0.9% | | 1.1% |
| Scomberomorus maculatus | Spanish mackerel | Subtropical; 20°C - 30°C; 44°N - 19°N | 4.2% | | | | 6.3% | | | | 0.4% |
| Narcine bancroftii | lesser electric ray | Tropical; 35°N - 10°N | 4.2% | 6.3% | | | | | | | |
| Lactophrys bicaudalis | spotted trunkfish | Subtropical; 32°N - 35°S | | | 5.0% | | | 5.3% | | | |
| Archosargus probatocephalus | sheepshead | Subtropical; 49°N - 6°S | 8.3% | | | | | | 1.7% | | |
| Synodus intermedius | sand diver | Subtropical; 37°N - 30°S | 2.1% | | | | | | 2.6% | 4.5% | 0.7% |
| Aluterus schoepfii | orange filefish | Subtropical; 46°N - 27°S | 6.3% | | | | 3.1% | | | | 0.4% |
| Haemulon album | margate | Tropical; 33°N - 33°S | 2.1% | | | | 3.1% | 1.3% | 0.9% | | 2.2% |
| Scartella cristata | molly miller | Tropical | | | | 5.6% | | | 1.7% | | 2.2% |
| Cantherhines macrocerus | whitespotted filefish | Tropical; 33°N - 26°S | | | | | 3.1% | | 2.6% | 2.3% | 1.1% |
| Holocentrus rufus | longspine squirrelfish | Tropical; 36°N - 28°S | | | | | | | 6.0% | 2.3% | 0.7% |
| Hypanus americanus | southern stingray | Subtropical; 36°N - 4°S | | | 5.0% | | | | 2.6% | | 1.4% |
| Schultzea beta | school bass | Subtropical | | | | 5.6% | | 1.3% | 0.9% | 1.1% | |
| Decapterus macarellus | mackerel scad | Subtropical; 54°N - 46°S | 8.3% | | | | | | | | 0.4% |
| Heteropriacanthus cruentatus | glasseye snapper | Subtropical; 33°N - 32°S | | | 5.0% | | | 1.3% | 1.7% | | 0.4% |
| Seriola zonata | banded rudderfish | Subtropical; 46°N - 23°S | 2.1% | 6.3% | | | | | | | |
| Elacatinus evelynae | sharknose goby | Tropical; 22°C - 27°C | | | | | 6.3% | | 0.9% | | 0.4% |
| Rypticus saponaceus | greater soapfish | Tropical; 33°N - 35°S | | | 5.0% | | | | | 1.1% | 1.1% |
| Synodus foetens | inshore lizardfish | Tropical; 25°C - 35°C; 43°N - 29°S | | | | | 3.1% | 2.6% | | | 1.4% |
| Hemiramphus brasiliensis | ballyhoo | Subtropical; 43°N - 13°S | | | 5.0% | | | | | | 1.4% |
| Mulloidichthys martinicus | yellow goatfish | Subtropical; 33°N - 26°S | | | 5.0% | | | | 0.9% | | 0.4% |
| Epinephelus adscensionis | rock hind | Tropical; 40°N - 24°S | | | | | | 3.9% | 1.7% | | 0.4% |
| Mycteroperca bonaci | black grouper | Subtropical; 33°N - 27°S | | | | | | 5.3% | | | 0.7% |
| Ophioblennius macclurei | redlip blenny | Tropical | | | | 5.6% | | | | | 0.4% |
| Bodianus pulchellus | spotfin hogfish | Tropical; 24°C - 28°C; 29°N - 7°N | 2.1% | | | | | 2.6% | | 1.1% | |
| Acanthemblemaria aspera | roughhead blenny | Subtropical; 20°N - 10°N | | | | | 3.1% | | 0.9% | | 1.8% |
| Gymnothorax miliaris | goldentail moray | Subtropical; 41°N - 26°S | | | | | | 1.3% | 1.7% | 2.3% | 0.4% |
| Neoniphon vexillarium | dusky squirrelfish | Tropical | | | | 5.6% | | | | | |
| Callionymus bairdi | lancer dragonet | Tropical; 32°N - | | | | 5.6% | | | | | |
| Epinephelus guttatus | red hind | Tropical; 36°N - 8°N | | | | | | 1.3% | 3.4% | | 0.4% |
| Aetobatus narinari | spotted eagle ray | Subtropical; 36°N - 24°S | | | | | | 2.6% | 1.7% | | 0.7% |
| Chromis enchrysurus | yellowtail reeffish | Tropical; 32°N - | | | 5.0% | | | | | | |
| Calamus leucosteus | whitebone porgy | Subtropical; 37°N - 10°N | | | 5.0% | | | | | | |
| Lutjanus jocu | dog snapper | Subtropical; 42°N - 26°S | 2.1% | | | | | | 1.7% | 1.1% | |
| Priacanthus arenatus | bigeye | Subtropical; 44°N - 40°S | | | | | 3.1% | | 1.7% | | |
| Sphoeroides testudineus | checkered puffer | Subtropical; 42°N - 35°S | | | | | | | 1.7% | 1.1% | 1.8% |
| Chilomycterus schoepfii | striped burrfish | Tropical; 46°N - 5°S | | | | | 3.1% | 1.3% | | | |
| Lactophrys trigonus | trunkfish | Subtropical; 45°N - 34°S | | | | | | | 0.9% | 2.3% | 1.1% |
| Gymnothorax funebris | green moray | Tropical; 42°N - 30°S | | | | | | 2.6% | 0.9% | | 0.7% |
| Mycteroperca microlepis | gag | Subtropical; 40°N - 27°S | 4.2% | | | | | | | | |
| Lutjanus cyanopterus | cubera snapper | Subtropical; 46°N - 26°S | 4.2% | | | | | | | | |
| Sardinella aurita | Spanish sardine | Subtropical; 18°C - 25°C; 47°N - 40°S | 4.2% | | | | | | | | |
| Opistognathus whitehursti | dusky jawfish | Tropical; 32°N - 7°N | | | | | | | | 3.4% | |
| Alphestes afer | mutton hamlet | Tropical; 32°N - 27°S | | | | | 3.1% | | | | |
| Gobioclinus kalisherae | downy blenny | Subtropical | | | | | 3.1% | | | | |
| Apogon binotatus | barred cardinalfish | Tropical; 32°N - 9°N | | | | | | 2.6% | | | 0.4% |
| Mycteroperca phenax | scamp | Subtropical; 41°N - 8°N | | | | | | 1.3% | 0.9% | | 0.7% |
| Remora remora | remora | Subtropical; 60°N - 36°S | | | | | | | | 2.3% | 0.4% |
| Centropyge argi | cherubfish | Subtropical; 35°N - 10°N | | | | | | 1.3% | | 1.1% | |
| Rypticus maculatus | whitespotted soapfish | Subtropical; 41°N - 24°N | 2.1% | | | | | | | | 0.4% |
| Elacatinus xanthiprora | yellowprow goby | Subtropical; 37°N - 8°N | | | | | | | | 2.3% | |
| Synodus foetens | red lizardfish | Tropical; 25°C - 35°C; 43°N - 29°S | | | | | | | | 2.3% | |
| Seriola dumerili | greater amberjack | Subtropical; 49°N - 36°S | 2.1% | | | | | | | | |
| Rhomboplites aurorubens | vermilion snapper | Subtropical; 42°N - 33°S | 2.1% | | | | | | | | |
| Centropomus undecimalis | common snook | Tropical; 25°C - 31°C | 2.1% | | | | | | | | |
| Trachinotus goodei | palometa | Subtropical; 43°N - 37°S | 2.1% | | | | | | | | |
| Myrichthys breviceps | sharptail eel | Subtropical; 33°N - 25°S | | | | | | | 0.9% | 1.1% | |
| Ctenogobius saepepallens | dash goby | Tropical; - 9°N | | | | | | | 0.9% | 1.1% | |
| Caranx hippos | crevalle jack | Subtropical | | | | | | | | 1.1% | 0.7% |
| Apogon townsendi | belted cardinalfish | Tropical | | | | | | | 1.7% | | |
| Halichoeres pictus | rainbow wrasse | Tropical; 32°N - 9°N | | | | | | | 0.9% | | 0.7% |
| Malacoctenus gilli | dusky blenny | Tropical | | | | | | | 0.9% | | 0.7% |
| Scarus coelestinus | midnight parrotfish | Tropical; 34°N - 26°S | | | | | | | | 1.1% | 0.4% |
| Balistes vetula | queen triggerfish | Tropical; ? - 28°C; 43°N | | | | | | 1.3% | | | |
| Hypoplectrus guttavarius | shy hamlet | Tropical | | | | | | 1.3% | | | |
| Anchoa lyolepis | dusky anchovy | Subtropical; 38°N - 27°S | | | | | | 1.3% | | | |
| Hypoplectrus indigo | indigo hamlet | Tropical | | | | | | 1.3% | | | |
| Amblycirrhitus pinos | redspotted hawkfish | Tropical; 36°N - 24°S | | | | | | | 0.9% | | 0.4% |
| Neoniphon coruscum | reef squirrelfish | Tropical; 33°N - 7°N | | | | | | | | 1.1% | |
| Rachycentron canadum | cobia | Subtropical; 17°C - 32°C; 47°N - 37°S | | | | | | | | 1.1% | |
| Oligoplites saurus | leatherjack | Subtropical; 43°N - 35°S | | | | | | | | 1.1% | |
| Opistognathus macrognathus | banded jawfish | Tropical | | | | | | | 0.9% | | |
| Chaenopsis limbaughi | yellowface pikeblenny | Tropical | | | | | | | 0.9% | | |
| Calamus nodosus | knobbed porgy | Subtropical; 37°N - 18°N | | | | | | | | | 0.7% |
| Jenkinsia sp. | herring species | | | | | | | | | | 0.7% |
| Microgobius carri | Seminole goby | Subtropical; 37°N - 10°N | | | | | | | | | 0.7% |
| Prionotus rubio | blackwing searobin | Subtropical; 37°N - 19°N | | | | | | | | | 0.7% |
| Coryphopterus lipernes | peppermint goby | Tropical; 25°N | | | | | | | | | 0.7% |
| Canthidermis sufflamen | ocean triggerfish | Tropical; 43°N - 17°S | | | | | | | | | 0.4% |
| Halichoeres caudalis | painted wrasse | Subtropical; 37°N - 6°N | | | | | | | | | 0.4% |
| Elacatinus horsti | yellowline goby | Tropical; - 9°N | | | | | | | | | 0.4% |
| Pseudobatos lentiginosus | Atlantic guitarfish | Subtropical; 36°N - 18°N | | | | | | | | | 0.4% |
| Chilomycterus reticulatus | spotted burrfish | Subtropical; - 24°S | | | | | | | | | 0.4% |
| Caranx lugubris | black jack | Subtropical; 30°N - 30°S | | | | | | | | | 0.4% |
| Mobula birostris | giant manta | Subtropical; 42°N - 38°S | | | | | | | | | 0.4% |
| Paralichthys albigutta | Gulf flounder | Subtropical; 37°N - 7°N | | | | | | | | | 0.4% |
| Sphyrna tiburo | bonnethead | Subtropical; 45°N - 36°S, | | | | | | | | | 0.4% |

| Percent Occurrence |
|---|
| 100% |
| 90% |
| 80% |
| 70% |
| 60% |
| 50% |
| 40% |
| 30% |
| 20% |
| 10% |
| 0% |

Present only in South Plam Beach, Deerfield, and Broward-Miami

Present only in North Plam Beach and Martin
