# Supplementary material for: Regional reef fish assemblage maps provide baseline biogeography for tropicalization monitoring
Source: Sci Rep. 2024 Apr 3;14:7893. doi: 10.1038/s41598-024-58185-6 (PMC10991435; doi:10.1038/s41598-024-58185-6)
Supplement: Supplementary file 7 — Supplementary Information 7. [file 41598_2024_58185_MOESM7_ESM.pdf]

| | | | Shallow assemblage mean densities | | | | | | | | |
|---|---|---|---|---|---|---|---|---|---|---|---|
| | | | Martin | | North Palm Beach | South Palm Beach | Deerfield | Broward-Miami | | | |
| | | | Hardbottom | | Hardbottom | Hardbottom | Hardbottom | Reef | | Hardbottom | |
| | | | High | Low | Low | Low | Low | High | Low | High | Low |
| Scientific name | Common Name | Thermal Affinity (Fishbase.org) | SMNHBH | SMNHBL | SNPHBL | SSPHBL | SDFHBL | SBMCRH | SBMCRL | SBMHBH | SBMHBL |
| *Haemulon sp.* | grunt species | Subtropical; 22°C - 25°C | 25.521 | 24.813 | 66.300 | 67.861 | 18.990 | 1.375 | 2.716 | 4.017 | 6.358 |
| *Haemulon aurolineatum* | tomtate | Subtropical; 43°N - 33°S | 25.958 | 29.469 | 23.550 | 25.528 | 4.463 | 7.671 | 2.384 | 38.574 | 3.188 |
| *Thalassoma bifasciatum* | bluehead | Tropical; 23°C - 26°C | 1.073 | 3.000 | 4.125 | 7.389 | 4.641 | 28.987 | 18.956 | 18.392 | 9.932 |
| *Haemulon flavolineatum* | French grunt | Subtropical; 34°N - 34°S | 1.198 | 0.906 | 12.225 | 44.528 | 2.208 | 11.717 | 2.716 | 9.722 | 2.719 |
| *Stegastes partitus* | bicolor damselfish | Tropical; 33°N - 5°S, | 0.177 | 0.031 | 8.775 | 3.278 | 0.297 | 30.579 | 18.609 | 10.102 | 2.885 |
| *Halichoeres bivittatus* | slippery dick | Tropical; 37°N - 26°S | 5.052 | 13.938 | 14.800 | 12.944 | 3.240 | 5.007 | 5.990 | 4.273 | 5.948 |
| *Coryphopterus personatus* | masked goby | Tropical; 32°N - | 0.479 | 0.031 | 0.675 | | 1.563 | 33.151 | 4.806 | 7.523 | 0.777 |
| *Acanthurus tractus* | ocean surgeon | Tropical; 21°C - 25°C; 44°N - 7°N | 1.510 | 2.563 | 3.550 | 3.917 | 4.489 | 6.919 | 7.165 | 9.756 | 5.879 |
| *Haemulon sciurus* | bluestriped grunt | Subtropical; 33°N - 19°S | 0.052 | 0.156 | 0.225 | 28.917 | 5.865 | 1.934 | 0.716 | 2.688 | 0.584 |
| *Abudefduf saxatilis* | sergeant major | Subtropical; 41°N - 37°S | 1.375 | 0.469 | 5.175 | 12.194 | 7.083 | 2.237 | 1.190 | 3.023 | 1.832 |
| *Haemulon carbonarium* | caesar grunt | Subtropical; 33°N - 34°S | 0.208 | | 0.050 | 30.000 | 0.620 | 0.355 | 0.112 | 1.523 | 0.245 |
| *Acanthurus chirurgus* | doctorfish | Subtropical; 22°C - 25°C ; 43°N - 30°S | 3.010 | 3.813 | 5.175 | 2.972 | 0.557 | 2.974 | 4.138 | 4.847 | 2.835 |
| *Haemulon parra* | sailors choice | Subtropical; 31°N - 30°S | 0.906 | 0.063 | 0.550 | 27.778 | 0.078 | 0.007 | 0.198 | 0.233 | 0.074 |
| *Anisotremus virginicus* | porkfish | Subtropical; 32°N - 33°S | 8.094 | 5.469 | 1.450 | 2.694 | 2.104 | 1.322 | 0.724 | 1.301 | 0.478 |
| *Sparisoma aurofrenatum* | redband parrotfish | Subtropical; 33°N - 8°N | 0.010 | | 0.400 | 0.222 | 0.375 | 7.743 | 4.680 | 5.835 | 3.058 |
| *Scarus iseri* | striped parrotfish | Subtropical; 23°C - 26°C | 0.021 | 0.188 | 0.800 | 3.194 | 3.266 | 4.638 | 5.200 | 2.426 | 1.579 |
| *Decapterus punctatus* | round scad | Subtropical; 45°N - 35°S | 7.604 | | | | 13.281 | | | | |
| *Haemulon plumierii* | white grunt | Subtropical; 39°N - 23°S | 2.573 | 1.031 | 0.725 | 1.222 | 3.531 | 3.191 | 1.261 | 3.960 | 3.104 |
| *Stegastes xanthurus* | cocoa damselfish | Tropical; 6°S - 30°S | 4.344 | 4.125 | 3.375 | 1.528 | 1.365 | 1.533 | 1.224 | 1.392 | 0.695 |
| *Halichoeres maculipinna* | clown wrasse | Tropical; 36°N - 25°S | 0.042 | 0.313 | 0.425 | 1.444 | 1.917 | 4.941 | 4.203 | 3.875 | 2.248 |
| *Caranx ruber* | bar jack | Subtropical; 35°N - 33°N | 1.063 | 1.000 | 3.025 | 2.333 | 2.547 | 0.447 | 2.250 | 0.693 | 0.861 |
| *Canthigaster rostrata* | sharpnose puffer | Tropical; 34°N - 8°N | 0.385 | 0.313 | 1.725 | 1.333 | 1.297 | 2.059 | 2.237 | 1.926 | 1.887 |
| *Halichoeres garnoti* | yellowhead wrasse | Tropical; 23°C - 27°C | | | 0.125 | 0.556 | 0.266 | 5.664 | 3.019 | 1.585 | 0.666 |
| *Acanthurus coeruleus* | blue tang | Tropical; 43°N - 27°S | 0.156 | 0.031 | 0.500 | 0.222 | 0.141 | 3.855 | 1.721 | 4.045 | 0.830 |
| *Caranx crysos* | blue runner | Subtropical; 46°N - 26°S | 0.323 | 2.656 | 1.550 | 1.194 | 1.323 | 2.375 | 0.483 | 0.239 | 0.541 |
| *Pseudupeneus maculatus* | spotted goatfish | Subtropical; 40°N - 30°S | 0.354 | 0.250 | 0.725 | 1.750 | 2.193 | 0.809 | 1.043 | 0.932 | 1.453 |
| *Chloroscombrus chrysurus* | Atlantic bumper | Subtropical; 42°N - 41°S | | 0.875 | | 5.667 | | 0.033 | 0.431 | 0.682 | 1.813 |
| *Stegastes leucostictus* | beaugregory | Tropical; 33°N - 35°S | 2.823 | 1.906 | 0.975 | 0.833 | 0.250 | 0.618 | 0.621 | 0.773 | 0.312 |
| *Ocyurus chrysurus* | yellowtail snapper | Subtropical; 42°N - 26°S | 0.344 | 0.344 | 0.125 | | 1.109 | 2.697 | 1.747 | 0.989 | 0.934 |
| *Azurina multilineata* | brown chromis | Subtropical; 33°N - 31°S | | | 0.150 | 0.333 | | 6.757 | 0.043 | 0.443 | 0.016 |
| *Pempheris schomburgkii* | glassy sweeper | Tropical | | | | 7.056 | | 0.072 | | | 0.027 |
| *Diplodus holbrookii* | spottail pinfish | Subtropical; 40°N - 20°N | 3.125 | 1.938 | 0.050 | 0.306 | | | 0.875 | 0.653 | 0.043 |
| *Lutjanus synagris* | lane snapper | Subtropical; 38°N - 38°S | 0.490 | 0.656 | 0.625 | 1.389 | 0.313 | 2.211 | 0.017 | 0.841 | 0.155 |
| *Decapterus macarellus* | mackerel scad | Subtropical; 54°N - 46°S | 6.198 | | | | | | | | 0.135 |
| *Lutjanus griseus* | gray snapper | Subtropical; 42°N - 9°N | 0.604 | 0.031 | 0.900 | 0.056 | 0.766 | 0.717 | 0.431 | 1.443 | 0.362 |
| *Sparisoma viride* | stoplight parrotfish | Subtropical; 34°N - 21°S | | | 0.200 | | 0.047 | 2.007 | 1.115 | 1.472 | 0.419 |
| *Cryptotomus roseus* | bluelip parrotfish | Tropical; 32°N - 33°S | | | 1.150 | | 0.578 | 0.809 | 0.859 | 0.460 | 0.649 |
| *Haemulon melanurum* | cottonwick | Subtropical; 34°N - 12°S | | 0.094 | 0.050 | 1.778 | 0.641 | 0.013 | 1.194 | 0.330 | 0.275 |
| *Sparisoma atomarium* | greenblotch parrotfish | Tropical; 32°N | | | 0.225 | 0.083 | 0.953 | 1.059 | 0.494 | 0.972 | 0.480 |
| *Scarus taeniopterus* | princess parrotfish | Tropical; 33°N - 34°S | | | | | | 1.934 | 1.307 | 0.500 | 0.340 |
| *Balistes capriscus* | gray triggerfish | Tropical; 58°N - 37°S | 0.135 | | 0.200 | 0.194 | 1.224 | 0.309 | 0.720 | 0.773 | 0.476 |
| *Kyphosus sectatrix* | Bermuda chub | Subtropical; 46°N - 29°S | 0.354 | 0.563 | 0.125 | 0.972 | 0.047 | 0.493 | 0.108 | 1.006 | 0.335 |
| *Pareques acuminatus* | high-hat | Tropical; 38°N - 33°S | 0.688 | 0.563 | 0.100 | 0.222 | 1.354 | 0.105 | 0.302 | 0.256 | 0.255 |
| *Parablennius marmoreus* | seaweed blenny | Subtropical; 36°N - 27°S | 1.031 | 0.563 | 0.700 | 0.833 | 0.297 | 0.020 | 0.060 | 0.057 | 0.194 |
| *Hemiramphus brasiliensis* | ballyhoo | Subtropical; 43°N - 13°S | | | 2.500 | | | | | | 1.187 |
| *oryphopterus glaucofraenum* | bridled goby | Tropical; 37°N - 33°S | 0.250 | | 0.350 | 0.028 | | 0.743 | 0.914 | 0.284 | 0.448 |
| *Pomacanthus arcuatus* | gray angelfish | Tropical; 22°C - 28°C | 0.094 | 0.031 | 0.025 | 0.028 | 0.047 | 0.684 | 0.668 | 0.693 | 0.519 |
| *Stegastes adustus* | dusky damselfish | Tropical; 32°N - 8°N | 0.104 | 0.250 | 0.050 | 0.139 | 0.068 | 0.474 | 0.134 | 1.170 | 0.094 |
| *Odontoscion dentex* | reef croaker | Tropical; 22°N - 25°S | 2.271 | | | | | | | | |
| *Chaetodon sedentarius* | reef butterflyfish | Subtropical; 36°N - 25°S | 0.073 | 0.094 | 0.025 | | 0.016 | 0.987 | 0.770 | 0.170 | 0.103 |
| *Haemulon striatum* | striped grunt | Subtropical; 35°N - 33°S | | | 0.300 | | 1.307 | 0.020 | | 0.205 | 0.178 |
| *Pomacanthus paru* | French angelfish | Subtropical; 33°N - 30°S | 0.271 | 0.031 | 0.250 | 0.222 | 0.115 | 0.303 | 0.353 | 0.261 | 0.190 |
| *Xyrichtys splendens* | green razorfish | Subtropical; 32°N - 33°S | 0.469 | 0.281 | 0.075 | 0.556 | 0.047 | 0.013 | 0.155 | 0.091 | 0.228 |
| *Chromis scotti* | purple reeffish | Tropical; 35°N - 23°S | | | | 1.361 | | 0.408 | 0.034 | 0.011 | |
| *Bodianus rufus* | Spanish hogfish | Tropical; 22°C - 28°C ); 33°N - 26°S | 0.094 | | 0.325 | 0.028 | 0.016 | 0.618 | 0.365 | 0.210 | 0.074 |
| *Sardinella aurita* | Spanish sardine | Subtropical; 18°C - 25°C; 47°N - 40°S | 1.646 | | | | | | | | |
| *Serranus tigrinus* | harlequin bass | Tropical; 33°N - 7°N | 0.042 | | 0.075 | | | 0.776 | 0.569 | 0.125 | 0.032 |
| *Brachygenys chrysargyrea* | smallmouth grunt | Subtropical; 32°N - 34°S | 0.021 | | | | | | 0.052 | 1.119 | 0.415 |
| *Gerres cinereus* | yellowfin mojarra | Subtropical; 33°N - 23°S | 0.115 | | 0.100 | 0.056 | 0.193 | | | 0.017 | 1.120 |
| *Sparisoma radians* | bucktooth parrotfish | Tropical; 33°N - 30°S | | | 0.025 | | 0.641 | 0.237 | 0.082 | 0.483 | 0.128 |
| *Malacoctenus triangulatus* | saddled blenny | Subtropical; 32°N - 26°S | 0.302 | 0.188 | 0.125 | 0.194 | 0.052 | 0.079 | 0.129 | 0.261 | 0.194 |
| *Chaetodon ocellatus* | spotfin butterflyfish | Tropical; 45°N - 8°N | 0.115 | 0.125 | 0.125 | | | 0.513 | 0.228 | 0.256 | 0.103 |
| *Elacatinus oceanops* | neon goby | Subtropical; 21°C - 28°C | 0.042 | | | | | 0.572 | 0.297 | 0.352 | 0.198 |
| *Jenkinsia sp.* | herring species | | | | | | | | | | 1.439 |
| *Lachnolaimus maximus* | hogfish | Subtropical; 46°N - 4°N | 0.031 | | | | 0.016 | 0.493 | 0.355 | 0.193 | 0.323 |
| *Hypoplectrus unicolor* | butter hamlet | Tropical; 32°N - 6°N | 0.031 | 0.031 | | | 0.073 | 0.691 | 0.341 | 0.125 | 0.101 |
| *Halichoeres poeyi* | blackear wrasse | Tropical; 30°N - 33°S | | 0.063 | 0.200 | 0.361 | 0.094 | 0.118 | 0.147 | 0.256 | 0.130 |
| *Sparisoma rubripinne* | yellowtail parrotfish | Tropical; 42°N - 7°N | 0.469 | 0.063 | 0.050 | 0.028 | 0.078 | 0.118 | 0.216 | 0.119 | 0.224 |
| *Anisotremus surinamensis* | black margate | Subtropical; 31°N - 35°S | 0.552 | 0.531 | | | 0.047 | 0.086 | 0.030 | 0.085 | 0.013 |
| *Chaetodon capistratus* | foureye butterflyfish | Subtropical; 21°C - 28°C | 0.021 | | | | | 0.697 | 0.392 | 0.170 | 0.027 |
| *Caranx hippos* | crevalle jack | Subtropical | | | | | | | | 1.278 | 0.011 |
| *Labrisomus nuchipinnis* | hairy blenny | Tropical; 34°N - 34°S | 0.802 | 0.313 | 0.050 | 0.083 | | | | 0.017 | 0.005 |
| *Lutjanus analis* | mutton snapper | Tropical; 20°C - 28°C; 42°N - 28°S | 0.083 | 0.219 | 0.300 | | 0.156 | 0.118 | 0.093 | 0.125 | 0.080 |
| *Ptereleotris calliura* | blue dartfish | Tropical | | | 0.350 | | | 0.053 | 0.569 | 0.034 | 0.132 |
| *Chaetodipterus faber* | Atlantic spadefish | Subtropical; 43°N - 34°S | 0.344 | 0.063 | | | | 0.112 | | 0.494 | 0.100 |
| *Schultzea beta* | school bass | Subtropical | | | | 0.833 | | 0.033 | 0.172 | 0.057 | |
| *Bodianus parrae* | creole wrasse | Tropical; 34°N - 8°N | | | 0.075 | | | 0.895 | 0.078 | 0.034 | 0.004 |
| *Calamus calamus* | saucereye porgy | Subtropical; 37°N - 33°S | 0.292 | 0.188 | | | 0.068 | 0.072 | 0.284 | 0.011 | 0.067 |
| *Cantherhines pullus* | orangespotted filefish | Subtropical; 42°N - 26°S | 0.094 | 0.031 | 0.075 | | 0.266 | 0.211 | 0.116 | 0.102 | 0.045 |
| *Diplodus argenteus* | silver porgy | Subtropical; 20°S - 35°S | 0.073 | 0.188 | 0.250 | 0.194 | 0.141 | 0.026 | | | 0.067 |
| *Halichoeres radiatus* | puddingwife | Tropical; 23°C - 27°C; 37°N - 19°S | 0.104 | 0.063 | 0.150 | 0.333 | 0.031 | 0.092 | 0.017 | 0.102 | 0.038 |
| *Scomberomorus maculatus* | Spanish mackerel | Subtropical; 20°C - 30°C; 44°N - 19°N | 0.031 | | | | 0.875 | | | | 0.002 |
| *Azurina cyanea* | blue chromis | Tropical; 34°N - 7°N | | | 0.125 | | | 0.526 | 0.177 | 0.045 | 0.014 |
| *Sparisoma chrysopterum* | redtail parrotfish | Tropical; 28°N - 4°N | | | | 0.083 | 0.125 | 0.059 | 0.091 | 0.170 | 0.305 |
| *Caranx bartholomaei* | yellow jack | Subtropical; 42°N - 25°S | 0.010 | 0.219 | 0.125 | 0.056 | 0.250 | 0.020 | | 0.108 | 0.045 |
| *Diplectrum formosum* | sand perch | Subtropical; 42°N - 35°S | 0.021 | | 0.050 | 0.028 | | 0.020 | 0.478 | 0.006 | 0.214 |
| *Holacanthus tricolor* | rock beauty | Tropical; 37°N - 29°S | | | 0.125 | | 0.047 | 0.329 | 0.239 | 0.045 | 0.018 |
| *Cephalopholis cruentata* | graysby | Subtropical; 36°N - 6°N | | | 0.050 | | | 0.434 | 0.246 | 0.040 | 0.005 |
| *Nicholsina usta* | emerald parrotfish | Tropical; 41°N - 30°S | 0.021 | 0.688 | | | 0.016 | | | | 0.004 |
| *Serranus baldwini* | lantern bass | Tropical; 33°N - 36°S | | | 0.125 | | | 0.125 | 0.105 | 0.102 | 0.254 |
| *Stegastes planifrons* | threespot damselfish | Tropical | 0.010 | | | | | 0.151 | 0.134 | 0.364 | 0.030 |
| *Holacanthus ciliaris* | queen angelfish | Subtropical; 33°N - 35°S | 0.021 | | 0.150 | | 0.016 | 0.178 | 0.116 | 0.131 | 0.050 |
| *Microspathodon chrysurus* | yellowtail damselfish | Subtropical; 33°N - 25°S | 0.010 | | | | | 0.132 | 0.164 | 0.307 | 0.016 |
| *Diodon holocanthus* | balloonfish | Subtropical; 37°N - 39°S | 0.021 | | 0.050 | | 0.188 | 0.086 | 0.082 | 0.108 | 0.070 |
| *Holacanthus bermudensis* | blue angelfish | Subtropical; 35°N - 18°N | 0.083 | | | | | 0.197 | 0.086 | 0.165 | 0.031 |
| *Sphoeroides spengleri* | bandtail puffer | Subtropical; 43°N - 30°S | 0.010 | 0.031 | 0.025 | | 0.141 | 0.026 | 0.034 | 0.080 | 0.189 |
| *Centropomus undecimalis* | common snook | Tropical; 25°C - 31°C | 0.521 | | | | | | | | |
| *canthostracion quadricornis* | scrawled cowfish | Subtropical; 43°N - 39°S | | | 0.175 | | 0.219 | 0.039 | 0.030 | 0.006 | 0.013 |
| *Seriola rivoliana* | almaco jack | Subtropical; 43°N - 38°S | 0.031 | 0.125 | | | 0.188 | 0.033 | 0.091 | | 0.007 |
| *Gnatholepis thompsoni* | goldspot goby | Tropical; 43°N - 2°S | | | 0.075 | 0.028 | | 0.211 | 0.047 | 0.057 | 0.044 |
| *Serranus subligarius* | belted sandfish | Subtropical; 37°N - 18°N | 0.396 | 0.031 | | 0.028 | | | | | |
| *Malacoctenus macropus* | rosy blenny | Subtropical; 32°N - | 0.052 | 0.094 | 0.125 | | 0.016 | | 0.026 | 0.040 | 0.095 |
| *eteropriacanthus cruentatus* | glasseye snapper | Subtropical; 33°N - 32°S | | | 0.375 | | | 0.026 | 0.013 | | 0.018 |
| *Scarus guacamaia* | rainbow parrotfish | Subtropical; 33°N - 41°S | 0.063 | 0.063 | | | 0.031 | 0.079 | 0.103 | 0.023 | 0.054 |
| *Xyrichtys martinicensis* | rosy razorfish | Subtropical | | | 0.050 | 0.222 | | | 0.078 | | 0.055 |
| *Pareques umbrosus* | cubbyu | Subtropical; 37°N - 4°N | 0.396 | | | | | | | 0.006 | |
| *Stegastes diencaeus* | longfin damselfish | Tropical | | | | | | 0.099 | 0.073 | 0.170 | 0.056 |
| *Chaetodon striatus* | banded butterflyfish | Tropical; 41°N - 29°S | 0.010 | | | | 0.094 | 0.158 | 0.069 | 0.028 | 0.029 |
| *Serranus tabacarius* | tobaccofish | Subtropical; 34°N - 10°S | | | | | 0.177 | 0.020 | 0.118 | 0.023 | 0.032 |
| *Stephanolepis hispidus* | planehead filefish | Subtropical; 46°N - 37°S | | | | | 0.297 | | 0.009 | 0.028 | 0.029 |
| *Urobatis jamaicensis* | yellow stingray | Tropical; 37°N - 7°N | | | 0.025 | | 0.094 | 0.066 | 0.043 | 0.068 | 0.067 |
| *Ptereleotris helenae* | hovering dartfish | Tropical | | | 0.050 | | | 0.046 | 0.172 | 0.011 | 0.081 |
| *Lutjanus apodus* | schoolmaster | Subtropical; 42°N - 19°S | 0.021 | | | | | 0.171 | 0.060 | 0.108 | |
| *Calamus penna* | sheepshead porgy | Tropical; 30°N - 33°S | | 0.094 | | | 0.188 | 0.013 | 0.013 | 0.017 | 0.016 |
| *Eques punctatus* | spotted drum | Tropical; 36°N - 24°S | | 0.125 | 0.050 | | 0.094 | | | 0.006 | 0.061 |
| *Holocentrus adscensionis* | squirrelfish | Subtropical; 37°N - 25°S | | | | | | 0.105 | 0.082 | 0.119 | 0.029 |
| *Aluterus scriptus* | scrawled filefish | Subtropical; 46°N - 30°S | 0.010 | | | | 0.141 | 0.072 | 0.052 | 0.034 | 0.014 |
| *Scorpaena plumieri* | spotted scorpionfish | Subtropical; 43°N - 30°S | 0.063 | 0.063 | 0.075 | | 0.036 | | 0.017 | 0.045 | 0.020 |
| *Aulostomus maculatus* | Atlantic trumpetfish | Subtropical; 33°N - 0°N | | | 0.025 | | 0.094 | 0.020 | 0.009 | 0.125 | 0.040 |
| *Epinephelus morio* | red grouper | Subtropical; 35°N - 27°S | | | 0.025 | | 0.016 | 0.053 | 0.099 | 0.068 | 0.050 |
| *Scarus vetula* | queen parrotfish | Tropical; 33°N - 8°N | | | | | | 0.033 | 0.043 | 0.199 | 0.027 |
| *Lactophrys triqueter* | smooth trunkfish | Subtropical; 44°N - 35°S | | | | | 0.109 | 0.059 | 0.062 | 0.011 | 0.059 |
| *Monacanthus tuckeri* | slender filefish | Subtropical; 37°N - 10°N | | | 0.075 | 0.111 | | | 0.043 | 0.028 | 0.016 |
| *Coryphopterus dicrus* | colon goby | Subtropical | | | 0.025 | | | 0.053 | 0.065 | 0.085 | 0.034 |
| *Opistognathus aurifrons* | yellowhead jawfish | Tropical; 22°C - 28°C; 32°N - 23°S | | | | | | 0.013 | 0.177 | | 0.052 |
| *Haemulon macrostoma* | Spanish grunt | Tropical; 32°N - 34°S | 0.042 | | | | | 0.053 | 0.004 | 0.131 | 0.002 |
| *Pterois volitans* | red lionfish | Tropical; 22°C - 28°C; 43°N - 40°S | | | 0.025 | 0.056 | | 0.079 | 0.039 | 0.017 | 0.005 |
| *Calamus proridens* | littlehead porgy | Subtropical; 30°N - 18°N | 0.010 | | 0.075 | 0.028 | 0.031 | 0.013 | 0.026 | 0.006 | 0.005 |
| *Serranus tortugarum* | chalk bass | Tropical; 32°N - 7°N | | | | | | 0.020 | 0.164 | | 0.009 |
| *Eques lanceolatus* | jackknife-fish | Tropical; 37°N - 34°S | 0.083 | 0.094 | | | | | | | 0.011 |
| *Hypoplectrus gemma* | blue hamlet | Tropical | | | | | | 0.099 | 0.052 | 0.006 | 0.002 |
| *Chromis insolata* | sunshinefish | Tropical; 37°N - 7°N | | | 0.075 | | 0.016 | 0.033 | 0.026 | | 0.005 |
| *Scartella cristata* | molly miller | Tropical | | | | 0.111 | | | 0.009 | | 0.032 |
| *Apogon maculatus* | flamefish | Tropical; 46°N - 7°N | | | | 0.083 | 0.016 | 0.007 | 0.017 | 0.011 | 0.007 |
| *Haemulon album* | margate | Tropical; 33°N - 33°S | 0.010 | | | | 0.078 | 0.007 | 0.009 | | 0.034 |
| *Xyrichtys novacula* | pearly razorfish | Subtropical | | 0.031 | 0.025 | | 0.063 | | | | 0.016 |
| *Apogon pseudomaculatus* | twospot cardinalfish | Tropical; 43°N - 34°S | | | 0.075 | 0.028 | | | 0.009 | | 0.023 |
| *rchosargus probatocephalus* | sheepshead | Subtropical; 49°N - 6°S | 0.125 | | | | | | 0.009 | | |
| *Scomberomorus regalis* | cero | Tropical; 41°N - 24°S | | | | | | 0.066 | 0.022 | 0.034 | 0.009 |
| *Elacatinus evelynae* | sharknose goby | Tropical; 22°C - 27°C | | | | | 0.109 | | 0.009 | | 0.011 |
| *Megalops atlanticus* | tarpon | Subtropical; 10°C - 40°C; 49°N - 44°S | | | 0.050 | | 0.063 | | | 0.006 | 0.005 |
| *Acanthostracion polygonium* | honeycomb cowfish | Tropical; 42°N - 35°S | | | | 0.056 | 0.031 | 0.020 | 0.009 | | 0.005 |
| *Rhomboplites aurorubens* | vermilion snapper | Subtropical; 42°N - 33°S | 0.115 | | | | | | | | |
| *Fistularia tabacaria* | bluespotted cornetfish | Tropical; 48°N - 29°S | | | 0.025 | | 0.063 | 0.007 | 0.009 | | 0.011 |
| *Chromis enchrysurus* | yellowtail reeffish | Tropical; 32°N - | | | 0.100 | | | | | | |
| *Priacanthus arenatus* | bigeye | Subtropical; 44°N - 40°S | | | | | 0.078 | | 0.022 | | |
| *Emblemaria pandionis* | sailfin blenny | Tropical; 32°N - 4°N | | | | | 0.063 | | 0.017 | | 0.018 |
| *Gymnothorax moringa* | spotted moray | Subtropical; 37°N - 26°S | 0.031 | | 0.025 | 0.028 | | 0.013 | | | |
| *Holocanthus sp.* | townsend angelfish | | | | | | | 0.059 | 0.030 | 0.006 | |
| *Ginglymostoma cirratum* | nurse shark | Subtropical; 44°N - 35°S | 0.010 | | | | 0.016 | | 0.009 | 0.028 | 0.031 |
| *Halichoeres cyanocephalus* | yellowcheek wrasse | Tropical; 30°N - 33°S | | | 0.025 | 0.028 | | 0.020 | 0.017 | | 0.002 |
| *Hypoplectrus puella* | barred hamlet | Tropical | | | 0.025 | | | 0.020 | 0.013 | 0.028 | 0.005 |
| *Seriola zonata* | banded rudderfish | Subtropical; 46°N - 23°S | 0.052 | 0.031 | | | | | | | |
| *Euthynnus alletteratus* | little tunny | Tropical; 56°N - 30°S | | 0.031 | 0.050 | | | | | | |
| *Lutjanus mahogoni* | mahogany snapper | Subtropical; 36°N - 9°N | 0.010 | | | | 0.021 | 0.020 | 0.026 | | 0.002 |
| *Lutjanus jocu* | dog snapper | Subtropical; 42°N - 26°S | 0.063 | | | | | | 0.009 | 0.006 | |
| *Calamus bajonado* | jolthead porgy | Subtropical; 42°N - 18°S | 0.010 | | | 0.028 | 0.031 | | 0.004 | | 0.002 |
| *Diodon hystrix* | porcupinefish | Subtropical; 35°N - 31°S | | | 0.025 | | 0.021 | 0.013 | 0.009 | 0.006 | 0.002 |
| *Cephalopholis fulva* | coney | Subtropical; 36°N - 28°S | | | 0.025 | | | 0.013 | 0.026 | 0.006 | |
| *Malacanthus plumieri* | sand tilefish | Subtropical; 34°N - 8°S | | | | 0.056 | | | 0.009 | | 0.004 |
| *Scarus coeruleus* | blue parrotfish | Tropical; 39°N - 24°S | | | | 0.028 | 0.016 | | 0.013 | 0.006 | 0.005 |
| *Sphyraena barracuda* | great barracuda | Subtropical; 42°N - 35°S | 0.031 | 0.031 | | | | | | | 0.004 |
| *Seriola dumerili* | greater amberjack | Subtropical; 49°N - 36°S | 0.063 | | | | | | | | |
| *Narcine bancroftii* | lesser electric ray | Tropical; 35°N - 10°N | 0.031 | 0.031 | | | | | | | |
| *Acanthemblemaria aspera* | roughhead blenny | Subtropical; 20°N - 10°N | | | | | 0.047 | | 0.004 | | 0.009 |
| *Aluterus schoepfii* | orange filefish | Subtropical; 46°N - 27°S | 0.042 | | | | 0.016 | | | | 0.002 |
| *Neoniphon vexillarium* | dusky squirrelfish | Tropical | | | | 0.056 | | | | | |
| *Callionymus bairdi* | lancer dragonet | Tropical; 32°N - | | | | 0.056 | | | | | |
| *Gymnothorax vicinus* | purplemouth moray | Subtropical | 0.021 | | 0.025 | | | | 0.004 | | 0.005 |
| *Synodus intermedius* | sand diver | Subtropical; 37°N - 30°S | 0.010 | | | | | | 0.013 | 0.028 | 0.004 |
| *Holocentrus rufus* | longspine squirrelfish | Tropical; 36°N - 28°S | | | | | | | 0.040 | 0.011 | 0.004 |
| *Lactophrys bicaudalis* | spotted trunkfish | Subtropical; 32°N - 35°S | | | 0.025 | | | 0.026 | | | |
| *Malacoctenus gilli* | dusky blenny | Tropical | | | | | | | 0.009 | | 0.041 |
| *Chilomycterus schoepfii* | striped burrfish | Tropical; 46°N - 5°S | | | | | 0.016 | 0.033 | | | |
| *Bodianus pulchellus* | spotfin hogfish | Tropical; 24°C - 28°C; 29°N - 7°N | 0.021 | | | | | 0.020 | | 0.006 | |
| *Cantherhines macrocerus* | whitespotted filefish | Tropical; 33°N - 26°S | | | | | 0.016 | | 0.013 | 0.011 | 0.005 |
| *Hypanus americanus* | southern stingray | Subtropical; 36°N - 4°S | | | 0.025 | | | | 0.010 | | 0.007 |
| *Gymnothorax miliaris* | goldentail moray | Subtropical; 41°N - 26°S | | | | | | 0.007 | 0.009 | 0.023 | 0.002 |
| *Rypticus saponaceus* | greater soapfish | Tropical; 33°N - 35°S | | | 0.025 | | | | | 0.006 | 0.009 |
| *Mulloidichthys martinicus* | yellow goatfish | Subtropical; 33°N - 26°S | | | 0.025 | | | | 0.009 | | 0.002 |
| *Synodus foetens* | inshore lizardfish | Tropical; 25°C - 35°C; 43°N - 29°S | | | | | 0.016 | 0.013 | | | 0.006 |
| *Epinephelus adscensionis* | rock hind | Tropical; 40°N - 24°S | | | | | | 0.020 | 0.009 | | 0.004 |
| *Lutjanus cyanopterus* | cubera snapper | Subtropical; 46°N - 26°S | 0.031 | | | | | | | | |
| *Mycteroperca bonaci* | black grouper | Subtropical; 33°N - 27°S | | | | | | 0.026 | | | 0.004 |
| *Ophioblennius macclurei* | redlip blenny | Tropical | | | | 0.028 | | | | | 0.002 |
| *Apogon binotatus* | barred cardinalfish | Tropical; 32°N - 9°N | | | | | | 0.026 | | | 0.002 |
| *Aetobatus narinari* | spotted eagle ray | Subtropical; 36°N - 24°S | | | | | | 0.013 | 0.009 | | 0.005 |
| *Sphoeroides testudineus* | checkered puffer | Subtropical; 42°N - 35°S | | | | | | | 0.009 | 0.006 | 0.013 |
| *Remora remora* | remora | Subtropical; 60°N - 36°S | | | | | | | | 0.023 | 0.004 |
| *Epinephelus guttatus* | red hind | Tropical; 36°N - 8°N | | | | | | 0.007 | 0.017 | | 0.002 |
| *Calamus leucosteus* | whitebone porgy | Subtropical; 37°N - 10°N | | | 0.025 | | | | | | |
| *Lactophrys trigonus* | trunkfish | Subtropical; 45°N - 34°S | | | | | | | 0.004 | 0.011 | 0.007 |
| *Synodus foetens* | red lizardfish | Tropical; 25°C - 35°C; 43°N - 29°S | | | | | | | | 0.023 | |
| *Mycteroperca microlepis* | gag | Subtropical; 40°N - 27°S | 0.021 | | | | | | | | |
| *Gymnothorax funebris* | green moray | Tropical; 42°N - 30°S | | | | | | 0.013 | 0.004 | | 0.002 |
| *Mycteroperca phenax* | scamp | Subtropical; 41°N - 8°N | | | | | | 0.007 | 0.009 | | 0.004 |
| *Opistognathus whitehursti* | dusky jawfish | Tropical; 32°N - 7°N | | | | | | | | 0.017 | |
| *Alphestes afer* | mutton hamlet | Tropical; 32°N - 27°S | | | | | 0.016 | | | | |
| *Gobioclinus kalisherae* | downy blenny | Subtropical | | | | | 0.016 | | | | |
| *Centropyge argi* | cherubfish | Subtropical; 35°N - 10°N | | | | | | 0.007 | | 0.006 | |
| *Rypticus maculatus* | whitespotted soapfish | Subtropical; 41°N - 24°N | 0.010 | | | | | | | | 0.002 |
| *Elacatinus xanthiprora* | yellowprow goby | Subtropical; 37°N - 8°N | | | | | | | | 0.011 | |
| *Oligoplites saurus* | leatherjack | Subtropical; 43°N - 35°S | | | | | | | | 0.011 | |
| *Trachinotus goodei* | palometa | Subtropical; 43°N - 37°S | 0.010 | | | | | | | | |
| *Myrichthys breviceps* | sharptail eel | Subtropical; 33°N - 25°S | | | | | | | 0.004 | 0.006 | |
| *Ctenogobius saepepallens* | dash goby | Tropical; - 9°N | | | | | | | 0.004 | 0.006 | |
| *Apogon townsendi* | belted cardinalfish | Tropical | | | | | | | 0.009 | | |
| *pistognathus macrognathus* | banded jawfish | Tropical | | | | | | | 0.009 | | |
| *Chaenopsis limbaughi* | yellowface pikeblenny | Tropical | | | | | | | 0.009 | | |
| *Halichoeres pictus* | rainbow wrasse | Tropical; 32°N - 9°N | | | | | | | 0.004 | | 0.004 |
| *Scarus coelestinus* | midnight parrotfish | Tropical; 34°N - 26°S | | | | | | | | 0.006 | 0.002 |
| *Balistes vetula* | queen triggerfish | Tropical; ? - 28°C; 43°N | | | | | | 0.007 | | | |
| *Hypoplectrus guttavarius* | shy hamlet | Tropical | | | | | | 0.007 | | | |
| *Anchoa lyolepis* | dusky anchovy | Subtropical; 38°N - 27°S | | | | | | 0.007 | | | |
| *Hypoplectrus indigo* | indigo hamlet | Tropical | | | | | | 0.007 | | | |
| *Amblycirrhitus pinos* | redspotted hawkfish | Tropical; 36°N - 24°S | | | | | | | 0.004 | | 0.002 |
| *Neoniphon coruscum* | reef squirrelfish | Tropical; 33°N - 7°N | | | | | | | | 0.006 | |
| *Rachycentron canadum* | cobia | Subtropical; 17°C - 32°C; 47°N - 37°S | | | | | | | | 0.006 | |
| *Microgobius carri* | Seminole goby | Subtropical; 37°N - 10°N | | | | | | | | | 0.005 |
| *Calamus nodosus* | knobbed porgy | Subtropical; 37°N - 18°N | | | | | | | | | 0.004 |
| *Halichoeres caudalis* | painted wrasse | Subtropical; 37°N - 6°N | | | | | | | | | 0.004 |
| *Elacatinus horsti* | yellowline goby | Tropical; - 9°N | | | | | | | | | 0.004 |
| *Prionotus rubio* | blackwing searobin | Subtropical; 37°N - 19°N | | | | | | | | | 0.004 |
| *Coryphopterus lipernes* | peppermint goby | Tropical; 25°N | | | | | | | | | 0.004 |
| *Mobula birostris* | giant manta | Subtropical; 42°N - 38°S | | | | | | | | | 0.004 |
| *Canthidermis sufflamen* | ocean triggerfish | Tropical; 43°N - 17°S | | | | | | | | | 0.002 |
| *Pseudobatos lentiginosus* | Atlantic guitarfish | Subtropical; 36°N - 18°N | | | | | | | | | 0.002 |
| *Chilomycterus reticulatus* | spotted burrfish | Subtropical; - 24°S | | | | | | | | | 0.002 |
| *Caranx lugubris* | black jack | Subtropical; 30°N - 30°S | | | | | | | | | 0.002 |
| *Paralichthys albigutta* | Gulf flounder | Subtropical; 37°N - 7°N | | | | | | | | | 0.002 |
| *Sphyrna tiburo* | bonnethead | Subtropical; 45°N - 36°S, | | | | | | | | | 0.002 |
| Total number of species | | | 105 | 66 | 104 | 73 | 104 | 138 | 156 | 142 | 183 |

| Mean fish per SSU |
|---|
| >10 |
| 9 |
| 8 |
| 7 |
| 6 |
| 5 |
| 4 |
| 3 |
| 2 |
| 1 |
| 0 |

Present only in South Plam Beach, Deerfield, and Broward-Miami

Present only in North Plam Beach and Martin
