# Supplementary material for: Regional reef fish assemblage maps provide baseline biogeography for tropicalization monitoring
Source: Sci Rep. 2024 Apr 3;14:7893. doi: 10.1038/s41598-024-58185-6 (PMC10991435; doi:10.1038/s41598-024-58185-6)
Supplement: Supplementary file 8 — Supplementary Information 8. [file 41598_2024_58185_MOESM8_ESM.pdf]

			Deep assemblage percent occurrence												
			Martin		North Palm Beach			South Palm Beach			Deerfield		Broward-Miami		
			Hardbottom		Reef	Hardbottom		Reef		Hardbottom	Reef		Reef		Hardbottom
			High	Low	Low	Low	High	High	Low	Low	High	Low	High	Low	High
Scientific name	Common Name	Thermal Affinity (Fishbase.org)	DMNHBH	DMNHBL	DNPCRL	DNPHBL	DNPHBH	DSPCRH	DSPCRL	DSPHBL	DDFCRH	DDFCRL	DBMCRH	DBMCRL	DBMHBH
Stegastes partitus	bicolor damselfish	Tropical; 33°N - 5°S,	64.8%	58.8%	100.0%	69.2%	98.3%	100.0%	100.0%	100.0%	99.2%	98.2%	100.0%	99.1%	100.0%
Canthigaster rostrata	sharpnose puffer	Tropical; 34°N - 8°N	51.9%	41.2%	100.0%	66.9%	83.3%	98.0%	98.7%	100.0%	91.9%	92.9%	93.6%	94.5%	92.9%
Thalassoma bifasciatum	bluehead	Tropical; 23°C - 26°C	55.6%	41.2%	87.5%	57.0%	93.3%	99.0%	88.2%	100.0%	98.4%	88.4%	98.6%	96.8%	92.9%
Halichoeres garnoti	yellowhead wrasse	Tropical; 23°C - 27°C	18.5%	17.6%	100.0%	55.2%	85.0%	98.0%	94.7%	72.7%	97.6%	88.4%	97.2%	91.8%	100.0%
Acanthurus chirurgus	doctorfish	Subtropical; 22°C - 25°C ; 43°N - 30°S	64.8%	35.3%	87.5%	59.9%	83.3%	66.3%	73.7%	90.9%	75.0%	71.4%	79.8%	76.4%	100.0%
Chaetodon sedentarius	reef butterflyfish	Subtropical; 36°N - 25°S	44.4%	29.4%	37.5%	27.9%	86.7%	84.7%	88.2%	95.5%	92.7%	82.1%	93.6%	86.4%	100.0%
Acanthurus tractus	ocean surgeon	Tropical; 21°C - 25°C; 44°N - 7°N	35.2%	14.7%	62.5%	63.4%	80.0%	77.6%	86.8%	77.3%	91.1%	83.9%	81.9%	87.7%	78.6%
Sparisoma aurofrenatum	redband parrotfish	Subtropical; 33°N - 8°N	11.1%	5.9%	37.5%	29.7%	73.3%	94.9%	80.3%	36.4%	97.6%	88.4%	95.4%	85.9%	64.3%
Holacanthus tricolor	rock beauty	Tropical; 37°N - 29°S	18.5%	5.9%	62.5%	15.7%	46.7%	83.7%	80.3%	77.3%	74.2%	62.5%	83.0%	74.1%	100.0%
Acanthurus coeruleus	blue tang	Tropical; 43°N - 27°S	16.7%		37.5%	25.0%	66.7%	84.7%	69.7%	63.6%	81.5%	73.2%	76.2%	65.5%	100.0%
Anisotremus virginicus	porkfish	Subtropical; 32°N - 33°S	68.5%	20.6%	50.0%	36.0%	83.3%	81.6%	64.5%	40.9%	66.1%	61.6%	55.7%	35.0%	50.0%
Bodianus rufus	Spanish hogfish	Tropical; 22°C - 28°C); 33°N - 26°S	40.7%	11.8%	50.0%	16.3%	70.0%	67.3%	38.2%	31.8%	50.0%	49.1%	52.8%	40.9%	71.4%
Halichoeres bivittatus	slippery dick	Tropical; 37°N - 26°S	38.9%	61.8%	75.0%	67.4%	55.0%	31.6%	27.6%	77.3%	25.8%	35.7%	30.9%	55.0%	
Pomacanthus arcuatus	gray angelfish	Tropical; 22°C - 28°C	5.6%	5.9%	25.0%	25.0%	46.7%	53.1%	51.3%	40.9%	54.8%	65.2%	62.4%	67.7%	64.3%
Serranus tigrinus	harlequin bass	Tropical; 33°N - 7°N	3.7%		12.5%	11.0%	48.3%	65.3%	47.4%	22.7%	66.9%	75.0%	68.1%	74.5%	71.4%
Sparisoma atomarium	greenblotch parrotfish	Tropical; 32°N	18.5%	8.8%	62.5%	61.0%	68.3%	71.4%	52.6%	31.8%	41.9%	26.8%	52.5%	35.5%	
Balistes capriscus	gray triggerfish	Tropical; 58°N - 37°S	66.7%	73.5%	50.0%	55.2%	33.3%	13.3%	27.6%	86.4%	5.6%	22.3%	27.7%	46.8%	7.1%
Chaetodon ocellatus	spotfin butterflyfish	Tropical; 45°N - 8°N	20.4%	8.8%	12.5%	15.1%	20.0%	61.2%	46.1%	45.5%	48.4%	44.6%	56.0%	50.9%	85.7%
Pseudupeneus maculatus	spotted goatfish	Subtropical; 40°N - 30°S	42.6%		25.0%	23.8%	61.7%	50.0%	32.9%	9.1%	60.5%	53.6%	54.3%	43.2%	42.9%
Chromis insolata	sunshinefish	Tropical; 37°N - 7°N	20.4%	14.7%	37.5%	19.2%	66.7%	49.0%	36.8%	50.0%	41.9%	26.8%	36.5%	19.5%	71.4%
Cephalopholis cruentata	graysby	Subtropical; 36°N - 6°N	1.9%		37.5%	4.1%	46.7%	56.1%	32.9%	31.8%	53.2%	38.4%	55.0%	33.2%	85.7%
Stegastes xanthurus	cocoa damselfish	Tropical; 6°S - 30°S	57.4%	26.5%	50.0%	18.0%	66.7%	35.7%	27.6%	13.6%	22.6%	24.1%	39.7%	38.2%	50.0%
Azurina cyanea	blue chromis	Tropical; 34°N - 7°N	11.1%			3.5%	50.0%	64.3%	43.4%	18.2%	64.5%	37.5%	63.1%	31.8%	71.4%
Pterois volitans	red lionfish	Tropical; 22°C - 28°C; 43°N - 40°S	50.0%	23.5%	37.5%	8.1%	23.3%	20.4%	30.3%	86.4%	20.2%	17.0%	24.8%	15.0%	57.1%
Lutjanus analis	mutton snapper	Tropical; 20°C - 28°C; 42°N - 28°S	16.7%	20.6%	62.5%	39.5%	30.0%	16.3%	23.7%	40.9%	15.3%	14.3%	36.2%	36.8%	57.1%
Coryphopterus personatus	masked goby	Tropical; 32°N -				5.2%	38.3%	64.3%	23.7%	40.9%	50.8%	29.5%	59.2%	29.1%	64.3%
Sparisoma viride	stoplight parrotfish	Subtropical; 34°N - 21°S	1.9%	5.9%		11.0%	31.7%	64.3%	47.4%	13.6%	49.2%	44.6%	53.2%	37.7%	42.9%
Haemulon plumierii	white grunt	Subtropical; 39°N - 23°S	51.9%	14.7%		19.2%	53.3%	39.8%	19.7%	9.1%	41.1%	33.9%	38.7%	33.6%	42.9%
Pomacanthus paru	French angelfish	Subtropical; 33°N - 30°S	11.1%	11.8%	25.0%	19.8%	26.7%	33.7%	38.2%	31.8%	36.3%	26.8%	38.7%	46.8%	50.0%
Scarus iseri	striped parrotfish	Subtropical; 23°C - 26°C	3.7%	5.9%		9.9%	36.7%	50.0%	28.9%	13.6%	38.7%	53.6%	53.9%	49.1%	42.9%
Caranx ruber	bar jack	Subtropical; 35°N - 33°N	29.6%	2.9%	12.5%	22.7%	31.7%	58.2%	48.7%	31.8%	35.5%	30.4%	25.2%	30.5%	21.4%
Scarus taeniopterus	princess parrotfish	Tropical; 33°N - 34°S	9.3%			3.5%	15.0%	58.2%	28.9%	4.5%	62.9%	55.4%	59.2%	38.2%	42.9%
Halichoeres maculipinna	clown wrasse	Tropical; 36°N - 25°S	3.7%	5.9%	37.5%	23.8%	48.3%	29.6%	28.9%	27.3%	38.7%	44.6%	39.4%	35.9%	7.1%
Chromis enchrysurus	yellowtail reeffish	Tropical; 32°N -	59.3%	47.1%	62.5%	41.3%	55.0%	22.4%	21.1%	31.8%	8.1%	8.0%	4.3%	2.3%	7.1%
Holocentrus adscensionis	squirrelfish	Subtropical; 37°N - 25°S	1.9%		37.5%	19.2%	35.0%	28.6%	32.9%	36.4%	32.3%	21.4%	25.5%	25.9%	71.4%
Holacanthus ciliaris	queen angelfish	Subtropical; 33°N - 35°S	33.3%	2.9%		15.1%	28.3%	33.7%	27.6%	40.9%	19.4%	22.3%	36.9%	29.5%	57.1%
Holacanthus bermudensis	blue angelfish	Subtropical; 35°N - 18°N	27.8%	11.8%		18.6%	51.7%	27.6%	23.7%	13.6%	20.2%	15.2%	39.4%	32.7%	57.1%
Lachnolaimus maximus	hogfish	Subtropical; 46°N - 4°N	1.9%	2.9%		1.2%	11.7%	23.5%	18.4%	13.6%	22.6%	14.3%	57.4%	62.3%	92.9%
Serranus baldwini	lantern bass	Tropical; 33°N - 36°S	3.7%	5.9%	12.5%	22.1%	15.0%	31.6%	39.5%	50.0%	29.8%	25.9%	20.2%	19.1%	35.7%
Coryphopterus glaucofraenum	bridled goby	Tropical; 37°N - 33°S	5.6%	8.8%	50.0%	19.8%	40.0%	33.7%	25.0%	13.6%	7.3%	16.1%	25.2%	22.3%	35.7%
Calamus calamus	saucereye porgy	Subtropical; 37°N - 33°S	53.7%	38.2%	12.5%	23.8%	31.7%	12.2%	14.5%	22.7%	12.1%	10.7%	18.1%	20.9%	14.3%
Chromis scotti	purple reeffish	Tropical; 35°N - 23°S	20.4%	11.8%	12.5%	7.0%	46.7%	44.9%	19.7%	18.2%	27.4%	19.6%	17.7%	6.4%	21.4%
Aluterus scriptus	scrawled filefish	Subtropical; 46°N - 30°S	1.9%	5.9%		8.7%	20.0%	50.0%	36.8%	27.3%	25.8%	31.3%	21.6%	14.5%	28.6%
Cryptotomus roseus	bluelip parrotfish	Tropical; 32°N - 33°S	1.9%	8.8%	12.5%	36.6%	23.3%	22.4%	25.0%	22.7%	21.8%	17.0%	33.3%	16.4%	14.3%
Bodianus parrae	creole wrasse	Tropical; 34°N - 8°N	5.6%			4.1%	45.0%	54.1%	15.8%	9.1%	30.6%	19.6%	31.2%	13.6%	21.4%
Hypoplectrus unicolor	butter hamlet	Tropical; 32°N - 6°N	3.7%			5.8%	33.3%	30.6%	17.1%		31.5%	29.5%	36.9%	15.9%	42.9%
Caranx crysos	blue runner	Subtropical; 46°N - 26°S	70.4%	23.5%	25.0%	7.6%	15.0%	13.3%	15.8%	45.5%	3.2%	4.5%	8.5%	12.3%	
Gnatholepis thompsoni	goldspot goby	Tropical; 43°N - 2°S	7.4%	8.8%	37.5%	9.3%	20.0%	30.6%	26.3%	27.3%	19.4%	16.1%	23.8%	14.1%	
Haemulon aurolineatum	tomtate	Subtropical; 43°N - 33°S	77.8%	32.4%	12.5%	7.0%	30.0%	16.3%	7.9%	13.6%	11.3%	8.9%	4.3%	1.4%	14.3%
Acanthostracion polygonium	honeycomb cowfish	Tropical; 42°N - 35°S			25.0%	6.4%	25.0%	34.7%	38.2%	22.7%	18.5%	30.4%	7.8%	4.1%	14.3%
Centropyge argi	cherubfish	Subtropical; 35°N - 10°N			37.5%	16.3%	21.7%	14.3%	26.3%	63.6%	13.7%	6.3%	6.7%	3.2%	14.3%
Acanthostracion quadricornis	scrawled cowfish	Subtropical; 43°N - 39°S	11.1%	2.9%	37.5%	9.3%	21.7%	32.7%	17.1%	18.2%	33.9%	17.0%	8.9%	10.5%	
Stegastes leucostictus	beaugregory	Tropical; 33°N - 35°S	27.8%	5.9%	12.5%	9.9%	41.7%	19.4%	14.5%	13.6%	16.1%	11.6%	12.4%	20.5%	7.1%
Xyrichtys splendens	green razorfish	Subtropical; 32°N - 33°S	1.9%	17.6%	62.5%	47.7%	20.0%	7.1%	14.5%	18.2%	0.8%	4.5%	4.3%	10.9%	
Anisotremus surinamensis	black margate	Subtropical; 31°N - 35°S	25.9%	5.9%	25.0%	4.7%	41.7%	31.6%	17.1%	4.5%	16.9%	17.0%	7.1%	3.6%	
Cantherhines pullus	orangespotted filefish	Subtropical; 42°N - 26°S	5.6%		12.5%	4.7%	13.3%	36.7%	25.0%	22.7%	25.8%	17.9%	17.4%	15.5%	
Ocyurus chrysurus	yellowtail snapper	Subtropical; 42°N - 26°S	25.9%	8.8%	12.5%	7.0%	33.3%	26.5%	11.8%		23.4%	19.6%	11.0%	12.3%	
Azurina multilineata	brown chromis	Subtropical; 33°N - 31°S			12.5%	2.9%	45.0%	28.6%	6.6%	4.5%	21.8%	15.2%	15.2%	10.5%	28.6%
Haemulon sp.	grunt species	Subtropical; 22°C - 25°C	13.0%		12.5%	19.2%	36.7%	24.5%	17.1%	22.7%	13.7%	15.2%	6.4%	9.5%	
Opistognathus aurifrons	yellowhead jawfish	Tropical; 22°C - 28°C; 32°N - 23°S	3.7%	5.9%		19.8%	25.0%	6.1%	3.9%	22.7%	16.9%	25.9%	19.1%	29.5%	7.1%
Lactophrys triqueter	smooth trunkfish	Subtropical; 44°N - 35°S	1.9%			5.2%	8.3%	31.6%	28.9%	9.1%	30.6%	21.4%	15.2%	17.7%	
Sphoeroides spengleri	bandtail puffer	Subtropical; 43°N - 30°S	5.6%	5.9%		8.1%	5.0%	13.3%	14.5%	31.8%	21.8%	15.2%	16.3%	18.6%	7.1%
Halichoeres cyanocephalus	yellowcheek wrasse	Tropical; 30°N - 33°S		11.8%	12.5%	18.6%	15.0%	6.1%	9.2%	13.6%	16.1%	12.5%	13.1%	16.4%	14.3%
Serranus tabacarius	tobaccofish	Subtropical; 34°N - 10°S				2.9%	8.3%	11.2%	11.8%	4.5%	21.0%	21.4%	27.0%	43.6%	7.1%
Lutjanus griseus	gray snapper	Subtropical; 42°N - 9°N	61.1%	29.4%		3.5%	31.7%	4.1%	1.3%		3.2%	8.0%	4.6%	3.2%	7.1%
Epinephelus morio	red grouper	Subtropical; 35°N - 27°S	1.9%	5.9%	12.5%	5.2%	5.0%	6.1%	25.0%	31.8%	7.3%	18.8%	6.7%	15.9%	14.3%
Aulostomus maculatus	Atlantic trumpetfish	Subtropical; 33°N - 0°N				3.5%	16.7%	42.9%	26.3%	4.5%	22.6%	16.1%	9.2%	4.1%	7.1%
Chaetodon capistratus	foureye butterflyfish	Subtropical; 21°C - 28°C				1.2%	8.3%	42.9%	11.8%		21.0%	12.5%	24.5%	15.5%	
Lutjanus synagris	lane snapper	Subtropical; 38°N - 38°S	59.3%	41.2%		0.6%	1.7%	6.1%	1.3%	4.5%	4.0%	3.6%	2.1%	4.1%	7.1%
Bodianus pulchellus	spotfin hogfish	Tropical; 24°C - 28°C; 29°N - 7°N	14.8%			2.3%	20.0%	13.3%	13.2%	18.2%	6.5%	2.7%	7.8%	0.9%	35.7%
Gymnothorax moringa	spotted moray	Subtropical; 37°N - 26°S	5.6%	2.9%	37.5%	4.7%	5.0%	16.3%	19.7%	27.3%	2.4%	1.8%	2.5%	9.5%	
Ptereleotris calliura	blue dartfish	Tropical			12.5%	16.3%	8.3%	2.0%	19.7%	27.3%	4.0%	2.7%	8.2%	19.1%	7.1%
Centropristis striata	black sea bass	Temperate; 45°N - 25°N	55.6%	64.7%			3.3%					1.8%			
Caranx bartholomaei	yellow jack	Subtropical; 42°N - 25°S	9.3%	5.9%	12.5%	7.0%	13.3%	17.3%	7.9%	4.5%	8.1%	8.9%	6.4%	6.8%	14.3%
Haemulon flavolineatum	French grunt	Subtropical; 34°N - 34°S	1.9%		12.5%	2.9%	23.3%	25.5%	3.9%	4.5%	20.2%	12.5%	7.8%	5.0%	
Haemulon sciurus	bluestriped grunt	Subtropical; 33°N - 19°S				3.5%	20.0%	25.5%	5.3%	4.5%	21.0%	19.6%	8.5%	10.0%	
Sparisoma rubripinne	yellowtail parrotfish	Tropical; 42°N - 7°N				1.7%	5.0%	17.3%	13.2%		18.5%	16.1%	21.6%	16.4%	7.1%
Scorpaena plumieri	spotted scorpionfish	Subtropical; 43°N - 30°S	29.6%	14.7%		5.2%	10.0%	7.1%	11.8%	9.1%	6.5%	7.1%	5.7%	6.8%	
Elacatinus oceanops	neon goby	Subtropical; 21°C - 28°C		2.9%	12.5%	3.5%	3.3%	12.2%	3.9%	9.1%	20.2%	16.1%	8.5%	6.8%	14.3%
Abudefduf saxatilis	sergeant major	Subtropical; 41°N - 37°S	3.7%	2.9%		1.2%	21.7%	25.5%	10.5%	4.5%	19.4%	12.5%	7.8%	1.8%	
Malacanthus plumieri	sand tilefish	Subtropical; 34°N - 8°S			12.5%	12.2%	8.3%	8.2%	6.6%	22.7%	3.2%	6.3%	14.5%	10.5%	
Serranus tortugarum	chalk bass	Tropical; 32°N - 7°N				4.7%		7.1%	9.2%	13.6%	16.1%	8.9%	14.9%	22.7%	7.1%
Sparisoma radians	bucktooth parrotfish	Tropical; 33°N - 30°S				9.3%	15.0%	15.3%	3.9%	4.5%	9.7%	1.8%	9.9%	4.5%	28.6%
Kyphosus sectatrix	Bermuda chub	Subtropical; 46°N - 29°S	3.7%		12.5%	7.0%	46.7%	20.4%			5.6%	3.6%	2.1%		
Calamus penna	sheepshead porgy	Tropical; 30°N - 33°S	7.4%	35.3%		11.0%	15.0%	5.1%	3.9%		3.2%	4.5%	3.9%	8.6%	
Archosargus probatocephalus	sheepshead	Subtropical; 49°N - 6°S	53.7%	38.2%		2.3%	3.3%								
Haemulon parra	sailors choice	Subtropical; 31°N - 30°S	1.9%		25.0%	1.7%	30.0%	6.1%	5.3%		4.0%	8.0%	3.9%	3.6%	7.1%
Stephanolepis hispidus	planehead filefish	Subtropical; 46°N - 37°S		2.9%		1.7%		6.1%	9.2%	4.5%	11.3%	9.8%	9.2%	15.5%	21.4%
Sparisoma chrysopterum	redtail parrotfish	Tropical; 28°N - 4°N	1.9%			2.9%	3.3%	13.3%	7.9%	9.1%	10.5%	6.3%	17.7%	9.5%	7.1%
Calamus proridens	littlehead porgy	Subtropical; 30°N - 18°N	5.6%	29.4%		17.4%	11.7%	3.1%	3.9%	9.1%	0.8%		1.8%	4.5%	
Holocentrus rufus	longspine squirrelfish	Tropical; 36°N - 28°S		2.9%		4.7%	8.3%	5.1%	3.9%		11.3%	7.1%	6.0%	11.4%	21.4%
Chaetodipterus faber	Atlantic spadefish	Subtropical; 43°N - 34°S	18.5%	2.9%	12.5%	2.3%	11.7%	12.2%	2.6%		4.0%	3.6%	7.4%	2.3%	
Scomberomorus regalis	cero	Tropical; 41°N - 24°S	3.7%		25.0%			7.1%	5.3%	4.5%	12.1%	9.8%	6.0%	5.5%	
Rypticus saponaceus	greater soapfish	Tropical; 33°N - 35°S	7.4%	11.8%		2.3%	3.3%	15.3%	9.2%	13.6%	6.5%	3.6%	3.9%	0.5%	
Haemulon melanurum	cottonwick	Subtropical; 34°N - 12°S	3.7%		12.5%	6.4%	10.0%	4.1%	2.6%	9.1%	4.0%	3.6%	1.8%	4.5%	14.3%
Seriola rivoliana	almaco jack	Subtropical; 43°N - 38°S	14.8%	11.8%	12.5%	2.9%	15.0%	5.1%		4.5%		3.6%	2.1%	2.3%	
Pareques acuminatus	high-hat	Tropical; 38°N - 33°S	14.8%	5.9%		2.9%	11.7%	9.2%	1.3%		10.5%	7.1%	5.7%	2.7%	
Cephalopholis fulva	coney	Subtropical; 36°N - 28°S	3.7%			1.2%	11.7%	12.2%	2.6%	9.1%	6.5%	8.0%	2.1%	6.8%	7.1%
Monacanthus tuckeri	slender filefish	Subtropical; 37°N - 10°N	1.9%			7.6%		7.1%	19.7%	9.1%	6.5%	3.6%	7.8%	6.8%	
Halichoeres radiatus	puddingwife	Tropical; 23°C - 27°C; 37°N - 19°S		2.9%	12.5%	8.1%	20.0%	10.2%			7.3%	3.6%	2.1%	0.9%	
Diodon holocanthus	balloonfish	Subtropical; 37°N - 39°S				1.2%		4.1%	10.5%	9.1%	9.7%	9.8%	11.7%	8.2%	
Ptereleotris helenae	hovering dartfish	Tropical				6.4%	3.3%	5.1%	6.6%	18.2%	2.4%	5.4%	5.7%	10.5%	
Scarus guacamaia	rainbow parrotfish	Subtropical; 33°N - 41°S				0.6%	10.0%	10.2%	2.6%	4.5%	2.4%	13.4%	4.3%	8.2%	7.1%
Rypticus maculatus	whitespotted soapfish	Subtropical; 41°N - 24°N	27.8%	11.8%		1.7%	10.0%	2.0%	6.6%			0.9%			
Cantherhines macrocerus	whitespotted filefish	Tropical; 33°N - 26°S				4.1%	1.7%	10.2%	3.9%		4.8%	8.0%	2.1%	3.2%	21.4%
Elagatis bipinnulata	rainbow runner	Subtropical; 44°N - 41°S	16.7%		12.5%	3.5%	6.7%	4.1%			0.8%		0.7%		14.3%
Diplectrum formosum	sand perch	Subtropical; 42°N - 35°S	5.6%	29.4%		2.9%	1.7%		1.3%	13.6%	0.8%		1.1%	1.8%	
Mycteroperca phenax	scamp	Subtropical; 41°N - 8°N	25.9%	5.9%			3.3%		2.6%		2.4%	0.9%	1.8%	0.9%	14.3%
Chaetodon striatus	banded butterflyfish	Tropical; 41°N - 29°S				2.3%	3.3%	5.1%	3.9%	4.5%	11.3%	10.7%	8.2%	7.7%	
Malacoctenus triangulatus	saddled blenny	Subtropical; 32°N - 26°S	1.9%	2.9%	12.5%	1.7%	10.0%	4.1%	6.6%	4.5%	2.4%	6.3%	1.4%	2.7%	
Balistes vetula	queen triggerfish	Tropical; ? - 28°C; 43°N	3.7%	11.8%		4.7%	6.7%	2.0%	2.6%	9.1%	2.4%	1.8%	1.4%	2.3%	7.1%
Myripristis jacobus	blackbar soldierfish	Subtropical; 37°N - 23°S	3.7%			1.7%	20.0%	11.2%			8.1%	5.4%	3.2%	1.8%	
Haemulon carbonarium	caesar grunt	Subtropical; 33°N - 34°S	3.7%	5.9%			15.0%	9.2%	2.6%		8.9%	2.7%	2.1%	2.7%	
Halichoeres poeyi	blackear wrasse	Tropical; 30°N - 33°S		5.9%		18.0%	16.7%	3.1%	2.6%		4.8%		0.7%	0.9%	
Parablennius marmoreus	seaweed blenny	Subtropical; 36°N - 27°S	3.7%	8.8%		5.2%	1.7%	4.1%	3.9%	9.1%		12.5%	0.4%	2.3%	
Haemulon striatum	striped grunt	Subtropical; 35°N - 33°S	3.7%	2.9%			5.0%	6.1%	6.6%	18.2%	2.4%	3.6%	2.1%	0.5%	
Serranus subligarius	belted sandfish	Subtropical; 37°N - 18°N	16.7%	14.7%	12.5%	1.7%	3.3%								
Echeneis naucrates	sharksucker	Subtropical; 45°N - 45°S	3.7%	2.9%		1.2%	5.0%	5.1%	1.3%	13.6%	4.8%	3.6%	2.8%	3.6%	
Priacanthus arenatus	bigeye	Subtropical; 44°N - 40°S	9.3%	5.9%		1.7%	1.7%		2.6%	22.7%		1.8%		1.8%	
Heteropriacanthus cruentatus	glasseye snapper	Subtropical; 33°N - 32°S		5.9%	12.5%	1.2%		1.0%		22.7%			2.8%	1.4%	
Haemulon macrostoma	Spanish grunt	Tropical; 32°N - 34°S					8.3%	8.2%	2.6%		4.8%	2.7%	2.5%	0.9%	14.3%
Urobatis jamaicensis	yellow stingray	Tropical; 37°N - 7°N				2.3%		1.0%	5.3%	4.5%	4.8%	9.8%	4.3%	11.4%	
Stegastes adustus	dusky damselfish	Tropical; 32°N - 8°N	7.4%			0.6%	10.0%	4.1%			7.3%	2.7%	1.4%	1.8%	7.1%
Coryphopterus dicrus	colon goby	Subtropical		5.9%		3.5%	10.0%	6.1%	6.6%	4.5%		0.9%	2.1%	2.3%	
Stegastes planifrons	threespot damselfish	Tropical	3.7%		12.5%	0.6%	1.7%	1.0%		9.1%	8.1%	0.9%	1.1%	1.4%	
Pareques umbrosus	cubbyu	Subtropical; 37°N - 4°N	29.6%	2.9%				1.0%	1.3%			1.8%		0.5%	
Epinephelus itajara	goliath grouper	Subtropical; 36°N - 33°S	22.2%		12.5%	0.6%	1.7%								
Haemulon album	margate	Tropical; 33°N - 33°S						4.1%	2.6%	4.5%	2.4%	7.1%	3.2%	5.0%	7.1%
Calamus leucosteus	whitebone porgy	Subtropical; 37°N - 10°N	1.9%	17.6%		4.7%	1.7%			9.1%	0.8%				
Canthidermis sufflamen	ocean triggerfish	Tropical; 43°N - 17°S	1.9%	2.9%		3.5%	8.3%	4.1%	3.9%		0.8%	3.6%	2.5%	4.1%	
Fistularia tabacaria	bluespotted cornetfish	Tropical; 48°N - 29°S	3.7%	2.9%		1.7%	8.3%	5.1%	3.9%		4.0%	2.7%	1.1%	0.9%	
Apogon pseudomaculatus	twospot cardinalfish	Tropical; 43°N - 34°S	5.6%	2.9%	12.5%	2.3%	1.7%	1.0%	5.3%				0.4%	2.3%	
Epinephelus adscensionis	rock hind	Tropical; 40°N - 24°S						3.1%			5.6%	1.8%	3.5%	5.0%	14.3%
Mycteroperca microlepis	gag	Subtropical; 40°N - 27°S	18.5%				5.0%			4.5%	0.8%	1.8%	1.1%	0.9%	
Calamus bajonado	jolthead porgy	Subtropical; 42°N - 18°S		5.9%		4.1%	3.3%	4.1%	5.3%		2.4%	2.7%	2.8%	1.8%	
Gymnothorax funebris	green moray	Tropical; 42°N - 30°S	5.6%			1.2%	3.3%	7.1%		4.5%	2.4%	4.5%	2.8%		
Serranus annularis	orangeback bass	Tropical; 32°N -			12.5%	2.9%	10.0%	1.0%		4.5%			0.4%		
Mycteroperca bonaci	black grouper	Subtropical; 33°N - 27°S				0.6%	3.3%	1.0%	1.3%		2.4%	2.7%	0.7%	3.6%	14.3%
Decapterus punctatus	round scad	Subtropical; 45°N - 35°S	11.1%			1.7%	5.0%	1.0%	2.6%	4.5%		0.9%	0.7%	1.8%	
Calamus nodosus	knobbed porgy	Subtropical; 37°N - 18°N		2.9%		5.2%	5.0%	2.0%	5.3%	4.5%	1.6%	0.9%	0.7%	0.9%	
Sphyraena barracuda	great barracuda	Subtropical; 42°N - 35°S	1.9%			0.6%	6.7%	7.1%	2.6%		3.2%		3.9%	2.7%	
Scarus coeruleus	blue parrotfish	Tropical; 39°N - 24°S					1.7%	10.2%	1.3%			3.6%	7.4%	4.1%	
Euthynnus alletteratus	little tunny	Tropical; 56°N - 30°S	3.7%	2.9%	12.5%	1.2%	3.3%	1.0%	1.3%		1.6%		0.4%		
Holocanthus sp.	townsend angelfish		1.9%			3.5%	3.3%	3.1%		4.5%	3.2%	0.9%	3.5%	2.7%	
Hypoplectrus puella	barred hamlet	Tropical				1.2%	6.7%	5.1%	1.3%		6.5%	1.8%	1.4%	2.7%	
Xyrichtys martinicensis	rosy razorfish	Subtropical				4.1%	3.3%		3.9%	4.5%	3.2%	2.7%	1.8%	2.7%	
Aluterus schoepfii	orange filefish	Subtropical; 46°N - 27°S			12.5%	3.5%		4.1%			1.6%	0.9%	2.8%		
Synodus intermedius	sand diver	Subtropical; 37°N - 30°S	1.9%			2.9%		2.0%	1.3%	9.1%	3.2%	1.8%	0.4%	2.7%	
Scomberomorus maculatus	Spanish mackerel	Subtropical; 20°C - 30°C; 44°N - 19°N						4.1%	2.6%	4.5%	4.0%	6.3%	3.2%	0.5%	
Seriola dumerili	greater amberjack	Subtropical; 49°N - 36°S	3.7%	2.9%	12.5%		1.7%				1.6%	0.9%		0.9%	
Ginglymostoma cirratum	nurse shark	Subtropical; 44°N - 35°S	1.9%			0.6%	3.3%	1.0%					2.8%		14.3%
Scarus vetula	queen parrotfish	Tropical; 33°N - 8°N					1.7%	3.1%	1.3%		6.5%	3.6%	5.0%	2.7%	
Epinephelus guttatus	red hind	Tropical; 36°N - 8°N				1.7%	3.3%	4.1%	2.6%	4.5%	1.6%	0.9%	3.2%	0.9%	
Jenkinsia sp.	herring species		1.9%		12.5%	0.6%		2.0%		4.5%			0.4%		
Gymnothorax miliaris	goldentail moray	Subtropical; 41°N - 26°S				1.2%	1.7%	2.0%	2.6%		3.2%	1.8%	1.1%	0.9%	7.1%
Apogon townsendi	belted cardinalfish	Tropical					1.7%				2.4%	0.9%	1.4%		14.3%
Synodus foetens	inshore lizardfish	Tropical; 25°C - 35°C; 43°N - 29°S	3.7%				1.7%	1.0%	1.3%		1.6%	1.8%	0.4%	0.9%	7.1%
Apogon maculatus	flamefish	Tropical; 46°N - 7°N	1.9%	5.9%		1.2%	5.0%		1.3%			1.8%	1.1%	0.5%	
Brachygenys chrysargyrea	smallmouth grunt	Subtropical; 32°N - 34°S					5.0%	8.2%			4.0%		1.1%		
Serranus phoebe	tattler	Subtropical; 33°N - 34°S	3.7%		12.5%	1.7%									
Aluterus monoceros	unicorn filefish	Subtropical; 43°N - 39°S	1.9%			2.3%	3.3%	3.1%	1.3%	4.5%			1.4%		
Lactophrys bicaudalis	spotted trunkfish	Subtropical; 32°N - 35°S				1.7%	3.3%	3.1%			4.8%	1.8%	1.1%	1.8%	
Halichoeres pictus	rainbow wrasse	Tropical; 32°N - 9°N				0.6%	8.3%	2.0%	5.3%				1.1%		
Malacoctenus macropus	rosy blenny	Subtropical; 32°N -		5.9%		2.3%	6.7%				0.8%				
Rhomboplites aurorubens	vermilion snapper	Subtropical; 42°N - 33°S	9.3%	5.9%											
Decapterus macarellus	mackerel scad	Subtropical; 54°N - 46°S	9.3%			0.6%		1.0%	1.3%			0.9%	0.7%	0.5%	
Lutjanus apodus	schoolmaster	Subtropical; 42°N - 19°S					3.3%	4.1%		4.5%	0.8%		0.4%	0.9%	
Schultzea beta	school bass	Subtropical				1.7%	6.7%	4.1%				0.9%	0.4%		
Microspathodon chrysurus	yellowtail damselfish	Subtropical; 33°N - 25°S		2.9%				3.1%	3.9%		0.8%	2.7%			
Halichoeres caudalis	painted wrasse	Subtropical; 37°N - 6°N			12.5%							0.9%			
Hypoplectrus gemma	blue hamlet	Tropical						2.0%	1.3%		2.4%	4.5%	1.1%	1.8%	
Bothus lunatus	peacock flounder	Tropical; 34°N - 25°S			12.5%	0.6%									
Remora remora	remora	Subtropical; 60°N - 36°S		5.9%		1.2%					3.2%	0.9%	1.4%	0.5%	
Scarus coelestinus	midnight parrotfish	Tropical; 34°N - 26°S					5.0%	3.1%	3.9%				0.4%	0.5%	
Seriola zonata	banded rudderfish	Subtropical; 46°N - 23°S	9.3%				3.3%								
Lutjanus mahogoni	mahogany snapper	Subtropical; 36°N - 9°N					3.3%	3.1%			4.8%	0.9%	0.4%		
Carcharhinus leucas	bull shark	Subtropical; 42°N - 39°S						5.1%	2.6%	4.5%					
Xyrichtys novacula	pearly razorfish	Subtropical		2.9%		1.7%			2.6%		0.8%		0.4%	2.7%	
Eques punctatus	spotted drum	Tropical; 36°N - 24°S	1.9%					3.1%			1.6%	0.9%	2.5%	0.9%	
Aetobatus narinari	spotted eagle ray	Subtropical; 36°N - 24°S						1.0%			1.6%			0.9%	7.1%
Caranx hippos	crevalle jack	Subtropical		2.9%				1.0%	1.3%	4.5%			0.7%		
Myrichthys breviceps	sharptail eel	Subtropical; 33°N - 25°S				1.7%	3.3%		2.6%		0.8%		0.4%	1.4%	
Diplodus holbrookii	spottail pinfish	Subtropical; 40°N - 20°N	1.9%	2.9%				1.0%			2.4%	1.8%			
Coryphopterus eidolon	pallid goby	Tropical		2.9%		2.3%	1.7%		2.6%						
Heteroconger longissimus	brown garden eel	Tropical						6.1%	1.3%				0.7%	1.4%	
Diodon hystrix	porcupinefish	Subtropical; 35°N - 31°S				1.2%		2.0%			2.4%	0.9%	1.4%	1.4%	
Mulloidichthys martinicus	yellow goatfish	Subtropical; 33°N - 26°S					3.3%	2.0%				2.7%	0.4%	0.5%	
Apogon binotatus	barred cardinalfish	Tropical; 32°N - 9°N				0.6%		1.0%	1.3%			2.7%	1.1%	1.8%	
Hypanus americanus	southern stingray	Subtropical; 36°N - 4°S	1.9%				3.3%		1.3%		1.6%				
Odontoscion dentex	reef croaker	Tropical; 22°N - 25°S	3.7%	2.9%							0.8%		0.4%		
Nicholsina usta	emerald parrotfish	Tropical; 41°N - 30°S				1.7%		1.0%		4.5%				0.5%	
Carcharhinus perezii	reef shark	Subtropical; 33°N - 35°S					6.7%						0.7%		
Stegastes diencaeus	longfin damselfish	Tropical						2.0%	1.3%		0.8%	0.9%	1.4%		
Scartella cristata	molly miller	Tropical	1.9%							4.5%					
Neoniphon coruscum	reef squirrelfish	Tropical; 33°N - 7°N				1.7%	3.3%	1.0%							
Opsanus tau	oyster toadfish	Subtropical; 43°N - 25°N		5.9%											
Gymnothorax vicinus	purplemouth moray	Subtropical				1.2%		1.0%				2.7%	0.4%	0.5%	
Lactophrys trigonus	trunkfish	Subtropical; 45°N - 34°S				0.6%	1.7%					0.9%	0.7%	1.8%	
Emblemaria pandionis	sailfin blenny	Tropical; 32°N - 4°N				1.2%		1.0%				0.9%	0.7%	1.8%	
Scomberomorus cavalla	king mackerel	Tropical; 44°N - 22°S	1.9%			0.6%		1.0%	1.3%		0.8%				
Elacatinus horsti	yellowline goby	Tropical; - 9°N						2.0%			2.4%	0.9%			
Opistognathus whitehursti	dusky jawfish	Tropical; 32°N - 7°N								4.5%			0.7%		
Eques lanceolatus	jackknife-fish	Tropical; 37°N - 34°S	3.7%			0.6%							0.7%		
Lutjanus jocu	dog snapper	Subtropical; 42°N - 26°S	1.9%					1.0%			0.8%		0.4%	0.9%	
Elacatinus xanthiprora	yellowprow goby	Subtropical; 37°N - 8°N	3.7%										0.7%		
Haemulon vittatum	boga	Tropical; 33°N - 8°N					1.7%		2.6%						
Hypleurochilus bermudensis	barred blenny	Tropical; 33°N - 9°N				0.6%		1.0%			1.6%	0.9%			
Microgobius carri	Seminole goby	Subtropical; 37°N - 10°N		2.9%							0.8%		0.4%		
Amblycirrhitus pinos	redspotted hawkfish	Tropical; 36°N - 24°S					1.7%	1.0%	1.3%						
Orthopristis chrysoptera	pigfish	Temperate; 41°N - 10°N	1.9%								1.6%			0.5%	
Lutjanus cyanopterus	cubera snapper	Subtropical; 46°N - 26°S	3.7%												
Centropomus undecimalis	common snook	Tropical; 25°C - 31°C	3.7%												
Echeneis neucratoides	whitefin sharksucker	Subtropical; 43°N - 4°N	1.9%					1.0%					0.7%		
Pempheris schomburgkii	glassy sweeper	Tropical				1.2%		1.0%			0.8%		0.4%		
Elacatinus evelynae	sharknose goby	Tropical; 22°C - 27°C										0.9%		1.8%	
Ctenogobius saepepallens	dash goby	Tropical; - 9°N										2.7%			
Gerres cinereus	yellowfin mojarra	Subtropical; 33°N - 23°S							2.6%						
Bothus ocellatus	eyed flounder	Tropical; 41°N - 33°S							2.6%						
Pseudobatos lentiginosus	Atlantic guitarfish	Subtropical; 36°N - 18°N				1.2%			1.3%						
Hemiramphus brasiliensis	ballyhoo	Subtropical; 43°N - 13°S						2.0%					0.4%		
Caranx latus	horse-eye jack	Subtropical; 41°N - 23°S	1.9%											0.5%	
Hypoplectrus randallorum	tan hamlet							1.0%				0.9%	0.4%		
Rachycentron canadum	cobia	Subtropical; 17°C - 32°C; 47°N - 37°S				0.6%					1.6%				
Enchelycore nigricans	viper moray	Subtropical										1.8%	0.4%		
Sphoeroides testudineus	checkered puffer	Subtropical; 42°N - 35°S				1.2%								0.9%	
Chloroscombrus chrysurus	Atlantic bumper	Subtropical; 42°N - 41°S									0.8%		0.4%	0.9%	
Labrisomus nuchipinnis	hairy blenny	Tropical; 34°N - 34°S	1.9%												
Centropristis ocyurus	bank sea bass	Subtropical; 37°N - 18°N	1.9%												
Liopropoma eukrines	wrasse basslet	Subtropical; 37°N - 24°N	1.9%												
Lutjanus campechanus	red snapper	Subtropical	1.9%												
Sphoeroides nephelus	southern puffer	Subtropical; 31°N - 9°N	1.9%												
Prognathodes aculeatus	longsnout butterflyfish	Tropical; 33°N - 9°S						1.0%			0.8%				
Diplodus argenteus	silver porgy	Subtropical; 20°S - 35°S										1.8%			
Dactylopterus volitans	flying gurnard	Subtropical; 52°N - 45°S										1.8%			
Chilomycterus schoepfii	striped burrfish	Tropical; 46°N - 5°S									0.8%	0.9%			
Alphestes afer	mutton hamlet	Tropical; 32°N - 27°S					1.7%								
Upeneus parvus	dwarf goatfish	Tropical; 37°N - 33°S					1.7%								
Lagodon rhomboides	pinfish	Subtropical; 42°N - 18°N									1.6%				
Negaprion brevirostris	lemon shark	Subtropical; 45°N - 33°S				0.6%					0.8%				
Trachinotus falcatus	permit	Subtropical; 42°N - 25°S						1.0%					0.4%		
Rypticus bistrispinus	freckled soapfish	Tropical; - 23°S							1.3%						
Prionotus ophryas	bandtail searobin	Subtropical; 37°N - 9°N									0.8%			0.5%	
Opistognathus macrognathus	banded jawfish	Tropical											0.7%	0.5%	
Sphyrna lewini	scalloped hammerhead	Tropical; 46°N - 39°S				1.2%									
Hypoplectrus guttavarius	shy hamlet	Tropical									0.8%		0.4%		
Alectis ciliaris	African pompano	Subtropical; 42°N - 34°S						1.0%							
Sphyraena borealis	southern sennet	Tropical; 33°N - 35°S						1.0%							
Trachurus lathami	rough scad	Subtropical; 45°N - 37°S						1.0%							
Monacanthus ciliatus	fringed filefish	Subtropical; 52°N - 34°S				0.6%							0.4%		
Malacoctenus aurolineatus	goldline blenny	Subtropical												0.9%	
Prionotus rubio	blackwing searobin	Subtropical; 37°N - 19°N										0.9%			
Chilomycterus reticulatus	spotted burrfish	Subtropical; - 24°S										0.9%			
Archosargus rhomboidalis	sea bream	Subtropical; 22°C - 30°C; 41°N - 58°S										0.9%			
Megalops atlanticus	tarpon	Subtropical; 10°C - 40°C; 49°N - 44°S									0.8%				
Oligoplites saurus	leatherjack	Subtropical; 43°N - 35°S									0.8%				
Neoniphon vexillarium	dusky squirrelfish	Tropical											0.7%		
Chilomycterus antennatus	bridled burrfish	Tropical											0.7%		
Liopropoma rubre	peppermint basslet	Tropical											0.7%		
Ophioblennius macclurei	redlip blenny	Tropical				0.6%									
Coryphopterus lipernes	peppermint goby	Tropical; 25°N				0.6%									
Enchelycore carychroa	chestnut moray	Tropical				0.6%									
Galeocerdo cuvier	tiger shark	Subtropical; 62°N - 44°S				0.6%									
Istiophorus platypterus	sailfish	Subtropical				0.6%									
Ogcocephalus nasutus	shortnose batfish	Tropical				0.6%									
Paranthias furcifer	Atlantic creolefish	Subtropical; 35°N - 28°S				0.6%									
Priolepis hipoliti	rusty goby	Tropical				0.6%									
Acanthemblemaria maria	secretary blenny	Tropical; 18°N - 10°N												0.5%	
Apogon quadrisquamatus	sawcheek cardinalfish	Tropical; - 24°S												0.5%	
Hemiemblemaria simula	wrasse blenny	Tropical											0.4%		
Melichthys niger	black durgon	Tropical; 30°N - 29°S											0.4%		

Percent Occurrence: 100%, 90%, 80%, 70%, 60%, 50%, 40%, 30%, 20%, 10%, 0%

Present only in South Plam Beach, Deerfield, and Broward-Miami

Present only in North Plam Beach and Martin
