# Supplementary material for: Regional reef fish assemblage maps provide baseline biogeography for tropicalization monitoring
Source: Sci Rep. 2024 Apr 3;14:7893. doi: 10.1038/s41598-024-58185-6 (PMC10991435; doi:10.1038/s41598-024-58185-6)
Supplement: Supplementary file 9 — Supplementary Information 9. [file 41598_2024_58185_MOESM9_ESM.pdf]

			Deep assemblage mean densities												
			Martin		North Palm Beach			South Palm Beach			Deerfield		Broward-Miami		
			Hardbottom		Reef	Hardbottom		Reef		Hardbottom	Reef		Reef		Hardbottom
			High	Low	Low	Low	High	High	Low	Low	High	Low	High	Low	High
Scientific name	Common Name	Thermal Affinity (Fishbase.org)	DMNHBH	DMNHBL	DNPCRL	DNPHBL	DNPHBH	DSPCRH	DSPCRL	DSPHBL	DDFCRH	DDFCRL	DBMCRH	DBMCRL	DBMHBH
Jenkinsia sp.	herring species		9.259		675	0.116		0.020		0.023			0.007		
Stegastes partitus	bicolor damselfish	Tropical; 33°N - 5°S,	3.769	4.353	35	8.558	58.792	59.306	38.145	30.432	51.415	43.906	45.297	35.758	30.464
Thalassoma bifasciatum	bluehead	Tropical; 23°C - 26°C	3.981	1.735	9.875	7.453	36.925	52.143	23.145	24.523	26.879	22.880	25.358	21.451	8.714
Coryphopterus personatus	masked goby	Tropical; 32°N -				5.131	41.733	47.908	8.842	16.182	24.177	10.112	26.254	10.663	35.143
Haemulon aurolineatum	tomtate	Subtropical; 43°N - 33°S	75.991	13.941	0.063	2.262	39.450	17.464	1.500	0.727	5.085	9.656	2.280	0.030	4.714
Halichoeres garnoti	yellowhead wrasse	Tropical; 23°C - 27°C	0.417	0.353	2.438	2.910	9.217	12.378	8.684	7.568	12.073	11.342	9.098	9.520	6.536
Bodianus parrae	creole wrasse	Tropical; 34°N - 8°N	0.120			0.610	21.567	28.571	0.566	1.091	6.786	5.844	6.423	2.970	1.536
Haemulon sp.	grunt species	Subtropical; 22°C - 25°C	4.120		1.125	3.000	22.333	9.255	0.842	5.977	3.226	8.499	1.635	8.833	
Decapterus punctatus	round scad	Subtropical; 45°N - 35°S	51.759			0.195	1.667	5.102	1.316	0.045		0.223	0.014	0.132	
Acanthurus tractus	ocean surgeon	Tropical; 21°C - 25°C; 44°N - 7°N	0.639	0.235	2.188	1.392	3.258	5.607	6.059	5.364	6.968	5.595	6.284	6.385	2.929
Sparisoma aurofrenatum	redband parrotfish	Subtropical; 33°N - 8°N	0.074	0.044	0.500	0.517	5.858	9.357	3.092	1.227	7.472	6.225	7.467	5.075	1.893
Canthigaster rostrata	sharpnose puffer	Tropical; 34°N - 8°N	0.963	0.294	3.313	1.180	2.075	5.531	5.717	6.432	5.081	4.713	3.838	4.245	4.643
Acanthurus chirurgus	doctorfish	Subtropical; 22°C - 25°C ; 43°N - 30°S	1.778	0.544	4.938	1.887	3.750	3.311	2.961	4.705	3.306	3.012	5.555	4.749	5.929
Azurina cyanea	blue chromis	Tropical; 34°N - 7°N	0.611			0.055	7.742	8.077	1.145	0.409	8.274	4.067	5.540	3.314	4.857
Chromis insolata	sunshinefish	Tropical; 37°N - 7°N	1.259	1.471	0.813	1.116	5.983	4.327	1.882	3.000	3.742	1.116	3.823	1.266	3.786
Decapterus macarellus	mackerel scad	Subtropical; 54°N - 46°S	28.704			0.006		1.020	0.053			0.009	0.762	0.002	
Haemulon flavolineatum	French grunt	Subtropical; 34°N - 34°S	0.019		0.063	0.160	3.900	7.184	0.046	1.136	7.863	5.826	1.225	0.164	
Halichoeres bivittatus	slippery dick	Tropical; 37°N - 26°S	0.843	2.691	3.000	3.875	2.700	1.770	1.592	4.500	0.581	1.719	0.961	2.385	
Anisotremus virginicus	porkfish	Subtropical; 32°N - 33°S	3.444	1.000	0.750	1.532	5.842	3.204	1.099	1.295	1.806	1.853	1.149	0.638	0.607
Chromis scotti	purple reeffish	Tropical; 35°N - 23°S	2.704	0.382	0.063	0.503	7.267	4.515	0.947	0.750	2.141	1.366	0.823	0.273	0.786
Caranx crysos	blue runner	Subtropical; 46°N - 26°S	6.833	1.368	5.313	0.099	2.033	0.878	0.533	2.477	0.194	0.228	1.610	0.386	
Acanthurus coeruleus	blue tang	Tropical; 43°N - 27°S	0.148		0.250	0.703	2.475	3.327	2.230	1.636	2.528	2.202	2.249	1.938	1.321
Chaetodon sedentarius	reef butterflyfish	Subtropical; 36°N - 25°S	0.657	0.456	0.438	0.317	1.483	1.796	1.888	2.386	1.855	2.031	2.160	1.949	3.250
Balistes capriscus	gray triggerfish	Tropical; 58°N - 37°S	2.630	2.500	1.938	1.930	1.658	0.281	0.829	5.557	0.141	0.661	0.819	1.276	0.214
Scarus iseri	striped parrotfish	Subtropical; 23°C - 26°C	0.019	0.118		0.235	1.475	3.730	2.296	0.841	2.347	2.506	1.935	2.948	0.786
Sparisoma atomarium	greenblotch parrotfish	Tropical; 32°N	0.250	0.103	0.938	2.956	4.717	3.092	1.316	0.886	1.448	0.683	1.629	0.824	
Azurina multilineata	brown chromis	Subtropical; 33°N - 31°S			0.500	0.047	3.400	4.462	0.336	0.227	2.290	2.851	1.766	0.493	2.250
Haemulon melanurum	cottonwick	Subtropical; 34°N - 12°S	0.037		1.000	0.715	1.475	1.964	0.322	1.864	2.435	0.723	0.037	0.455	6.786
Chromis enchrysurus	yellowtail reeffish	Tropical; 32°N -	6.880	3.985	1.063	1.032	2.308	0.383	0.375	0.295	0.153	0.076	0.049	0.011	0.071
Halichoeres maculipinna	clown wrasse	Tropical; 36°N - 25°S	0.019	0.044	1.750	0.811	2.500	1.168	1.263	1.068	1.556	2.250	1.119	0.975	0.536
Haemulon plumierii	white grunt	Subtropical; 39°N - 23°S	1.157	0.206		0.375	3.825	2.240	0.507	0.068	1.581	2.269	1.171	0.998	0.464
Ocyurus chrysurus	yellowtail snapper	Subtropical; 42°N - 26°S	0.398	0.176	0.063	0.297	1.733	7.740	0.875		0.952	1.210	0.500	0.211	
Holacanthus tricolor	rock beauty	Tropical; 37°N - 29°S	0.139	0.029	1.125	0.148	0.675	1.571	1.349	2.114	1.097	0.917	1.368	1.188	1.893
Caranx ruber	bar jack	Subtropical; 35°N - 33°N	0.861	0.015	0.313	0.337	0.708	3.913	1.079	0.250	1.181	1.479	1.020	1.848	0.214
Lutjanus synagris	lane snapper	Subtropical; 38°N - 38°S	3.204	2.206		0.003	0.008	4.000	0.020	0.023	1.629	1.107	0.099	0.093	0.071
Pseudupeneus maculatus	spotted goatfish	Subtropical; 40°N - 30°S	1.185		0.188	0.523	2.608	1.418	0.263	0.318	1.407	1.545	0.766	0.567	0.821
Haemulon striatum	striped grunt	Subtropical; 35°N - 33°S	0.148	0.118			1.250	2.434	2.941	3.250	0.323	0.482	0.571	0.045	
Lutjanus griseus	gray snapper	Subtropical; 42°N - 9°N	6.250	1.324		0.035	2.104	0.393	0.007		0.024	0.576	0.339	0.175	0.036
Abudefduf saxatilis	sergeant major	Subtropical; 41°N - 37°S	0.046	0.044		0.047	0.992	4.811	0.329	0.091	1.968	1.583	0.626	0.173	
Elagatis bipinnulata	rainbow runner	Subtropical; 44°N - 41°S	0.778		3.125	0.552	1.167	0.485			0.040		0.184		4.286
Scarus taeniopterus	princess parrotfish	Tropical; 33°N - 34°S	0.074			0.157	0.375	1.633	0.849	0.727	1.617	1.296	1.479	1.380	0.786
Stegastes xanthurus	cocoa damselfish	Tropical; 6°S - 30°S	1.694	0.221	1.500	0.349	1.758	0.439	0.191	0.136	0.331	0.406	0.475	0.642	0.429
Cryptotomus roseus	bluelip parrotfish	Tropical; 32°N - 33°S	0.009	0.044	0.625	1.061	0.542	0.546	0.908	0.773	0.927	0.603	1.170	0.842	0.214
Haemulon sciurus	bluestriped grunt	Subtropical; 33°N - 19°S				0.294	0.792	3.051	0.066	0.023	0.911	1.972	0.949	0.184	
Serranus tortugarum	chalk bass	Tropical; 32°N - 7°N				0.049		0.270	0.434	0.068	5.157	0.388	0.389	0.717	0.714
Bodianus rufus	Spanish hogfish	Tropical; 22°C - 28°C); 33°N - 26°S	0.639	0.118	0.438	0.172	1.542	0.816	0.250	0.250	0.621	0.573	0.568	0.448	1.214
Centropristis striata	black sea bass	Temperate; 45°N - 25°N	4.250	3.309			0.017					0.027			
Coryphopterus glaucofraenum	bridled goby	Tropical; 37°N - 33°S	0.037	0.044	1.063	0.250	1.025	1.112	0.467	0.182	0.153	0.137	0.527	0.821	1.429
Serranus tigrinus	harlequin bass	Tropical; 33°N - 7°N	0.028		0.063	0.093	0.617	0.699	0.513	0.136	1.069	1.204	0.729	1.103	0.857
Schultzea beta	school bass	Subtropical				0.163	0.175	5.296				0.004	1.418		
Xyrichtys splendens	green razorfish	Subtropical; 32°N - 33°S	0.019	1.118	1.625	2.558	0.800	0.219	0.184	0.273	0.004	0.045	0.043	0.150	
Chaetodon ocellatus	spotfin butterflyfish	Tropical; 45°N - 8°N	0.287	0.074	0.063	0.160	0.367	0.954	0.592	0.636	0.641	0.624	0.740	0.681	1.179
Pomacanthus arcuatus	gray angelfish	Tropical; 22°C - 28°C	0.083	0.044	0.813	0.302	0.425	0.500	0.421	0.409	0.597	0.638	0.772	0.926	0.750
Haemulon vittatum	boga	Tropical; 33°N - 8°N					0.017		6.579						
Pterois volitans	red lionfish	Tropical; 22°C - 28°C; 43°N - 40°S	0.861	0.221	0.250	0.067	0.150	0.143	0.309	1.455	0.198	0.134	0.241	0.122	2.250
Caranx bartholomaei	yellow jack	Subtropical; 42°N - 25°S	0.241	0.162	3.750	0.349	0.408	0.235	0.059	0.455	0.125	0.063	0.087	0.055	0.071
Chaetodipterus faber	Atlantic spadefish	Subtropical; 43°N - 34°S	1.565	0.015	1.125	0.192	1.208	0.806	0.066		0.113	0.344	0.465	0.050	
Sparisoma viride	stoplight parrotfish	Subtropical; 34°N - 21°S	0.019	0.059		0.087	0.358	1.260	0.750	0.114	0.569	0.754	0.749	0.844	0.250
Calamus calamus	saucereye porgy	Subtropical; 37°N - 33°S	2.769	0.559	0.063	0.285	0.583	0.204	0.086	0.409	0.073	0.074	0.160	0.290	0.214
Lachnolaimus maximus	hogfish	Subtropical; 46°N - 4°N	0.009	0.015		0.012	0.192	0.143	0.145	0.114	0.141	0.089	1.006	1.126	2.179
Centropyge argi	cherubfish	Subtropical; 35°N - 10°N			0.375	0.215	0.442	0.643	0.770	1.864	0.448	0.098	0.057	0.016	0.143
Kyphosus sectatrix	Bermuda chub	Subtropical; 46°N - 29°S	0.093		0.750	0.215	1.100	1.724			0.198	0.058	0.154		
Pomacanthus paru	French angelfish	Subtropical; 33°N - 30°S	0.083	0.088	0.375	0.198	0.500	0.423	0.362	0.250	0.298	0.244	0.387	0.474	0.393
Cephalopholis cruentata	graysby	Subtropical; 36°N - 6°N	0.009		0.313	0.049	0.417	0.541	0.250	0.205	0.556	0.481	0.488	0.225	0.536
Stegastes leucostictus	beaugregory	Tropical; 33°N - 35°S	0.444	0.147	0.188	0.151	1.342	0.204	0.118	0.250	0.214	0.134	0.122	0.430	0.036
Holocentrus adscensionis	squirrelfish	Subtropical; 37°N - 25°S	0.019		0.438	0.166	0.583	0.219	0.217	0.318	0.274	0.211	0.209	0.281	0.607
Holacanthus ciliaris	queen angelfish	Subtropical; 33°N - 35°S	0.231	0.015		0.119	0.325	0.388	0.276	0.386	0.125	0.135	0.322	0.359	0.857
Lutjanus analis	mutton snapper	Tropical; 20°C - 28°C; 42°N - 28°S	0.130	0.162	0.500	0.390	0.333	0.153	0.164	0.432	0.109	0.103	0.337	0.355	0.286
Ptereleotris calliura	blue dartfish	Tropical			0.063	0.317	0.117	0.046	0.355	1.750	0.069	0.018	0.129	0.488	0.036
Holacanthus bermudensis	blue angelfish	Subtropical; 35°N - 18°N	0.333	0.162		0.201	0.675	0.224	0.204	0.114	0.157	0.091	0.384	0.296	0.536
Gnatholepis thompsoni	goldspot goby	Tropical; 43°N - 2°S	0.037	0.044	0.813	0.087	0.200	0.510	0.382	0.273	0.242	0.266	0.280	0.180	
Opistognathus aurifrons	yellowhead jawfish	Tropical; 22°C - 28°C; 32°N - 23°S	0.019	0.088		0.372	0.408	0.153	0.099	0.409	0.294	0.455	0.294	0.570	0.107
Hypoplectrus unicolor	butter hamlet	Tropical; 32°N - 6°N	0.019			0.047	0.342	0.959	0.171		0.302	0.354	0.379	0.148	0.500
Anisotremus surinamensis	black margate	Subtropical; 31°N - 35°S	0.676	0.059	0.313	0.081	1.083	0.240	0.145	0.045	0.121	0.144	0.066	0.018	
Brachygenys chrysargyrea	smallmouth grunt	Subtropical; 32°N - 34°S					0.367	1.735			0.359		0.273		
Serranus baldwini	lantern bass	Tropical; 33°N - 36°S	0.056	0.118	0.063	0.151	0.083	0.214	0.428	0.477	0.246	0.217	0.156	0.138	0.286
Aluterus scriptus	scrawled filefish	Subtropical; 46°N - 30°S	0.028	0.029		0.058	0.192	0.561	0.296	0.341	0.210	0.278	0.193	0.123	0.143
Haemulon parra	sailors choice	Subtropical; 31°N - 30°S	0.056		0.938	0.035	0.567	0.087	0.026		0.040	0.225	0.085	0.114	0.071
Pareques umbrosus	cubbyu	Subtropical; 37°N - 4°N	2.009	0.044				0.005	0.046			0.119		0.016	
Pareques acuminatus	high-hat	Tropical; 38°N - 33°S	0.417	0.324		0.023	0.467	0.357	0.007		0.145	0.186	0.117	0.012	
Haemulon carbonarium	caesar grunt	Subtropical; 33°N - 34°S	0.046	0.059			0.692	0.342	0.039		0.560	0.021	0.223	0.027	
Elacatinus oceanops	neon goby	Subtropical; 21°C - 28°C		0.015	0.063	0.061	0.025	0.143	0.026	0.636	0.323	0.332	0.092	0.094	0.071
Chaetodon capistratus	foureye butterflyfish	Subtropical; 21°C - 28°C				0.012	0.083	0.587	0.138		0.294	0.210	0.337	0.182	
Serranus tabacarius	tobaccofish	Subtropical; 34°N - 10°S				0.017	0.058	0.102	0.079	0.045	0.254	0.390	0.248	0.488	0.071
Lutjanus apodus	schoolmaster	Subtropical; 42°N - 19°S					0.017	1.658		0.023	0.028		0.018	0.005	
Diplodus holbrookii	spottail pinfish	Subtropical; 40°N - 20°N	0.185	0.059				0.459			0.371	0.670			
Sparisoma radians	bucktooth parrotfish	Tropical; 33°N - 30°S				0.116	0.383	0.372	0.099	0.023	0.149	0.027	0.152	0.076	0.286
Archosargus probatocephalus	sheepshead	Subtropical; 49°N - 6°S	0.824	0.618		0.020	0.167								
Pempheris schomburgkii	glassy sweeper	Tropical				1.163		0.306			0.129		0.002		
Myripristis jacobus	blackbar soldierfish	Subtropical; 37°N - 23°S	0.019			0.073	0.550	0.265			0.290	0.240	0.113	0.014	
Chloroscombrus chrysurus	Atlantic bumper	Subtropical; 42°N - 41°S									0.032		0.115	1.364	
Aulostomus maculatus	Atlantic trumpetfish	Subtropical; 33°N - 0°N				0.023	0.267	0.444	0.263	0.023	0.194	0.156	0.069	0.025	0.036
Acanthostracion quadricornis	scrawled cowfish	Subtropical; 43°N - 39°S	0.056	0.015	0.313	0.064	0.133	0.270	0.112	0.091	0.198	0.110	0.053	0.068	
Bodianus pulchellus	spotfin hogfish	Tropical; 24°C - 28°C; 29°N - 7°N	0.102			0.026	0.117	0.143	0.086	0.364	0.048	0.013	0.053	0.011	0.464
Calamus penna	sheepshead porgy	Tropical; 30°N - 33°S	0.083	0.618		0.137	0.358	0.026	0.020		0.032	0.027	0.035	0.084	
Acanthostracion polygonium	honeycomb cowfish	Tropical; 42°N - 35°S			0.125	0.032	0.167	0.199	0.276	0.159	0.109	0.164	0.043	0.032	0.071
Cantherhines pullus	orangespotted filefish	Subtropical; 42°N - 26°S	0.028		0.063	0.023	0.075	0.296	0.171	0.182	0.190	0.104	0.109	0.085	
Ptereleotris helenae	hovering dartfish	Tropical				0.128	0.042	0.128	0.099	0.364	0.016	0.134	0.087	0.327	
Seriola dumerili	greater amberjack	Subtropical; 49°N - 36°S	0.019	0.088	0.938		0.250				0.016	0.004		0.005	
Seriola rivoliana	almaco jack	Subtropical; 43°N - 38°S	0.222	0.221	0.125	0.029	0.175	0.327		0.023		0.064	0.074	0.025	
Lactophrys triqueter	smooth trunkfish	Subtropical; 44°N - 35°S	0.009			0.029	0.042	0.281	0.289	0.045	0.222	0.144	0.093	0.100	
Stegastes planifrons	threespot damselfish	Tropical	0.065		0.938	0.003	0.033	0.005		0.068	0.093	0.009	0.005	0.007	
Halichoeres cyanocephalus	yellowcheek wrasse	Tropical; 30°N - 33°S		0.059	0.063	0.148	0.100	0.036	0.066	0.205	0.113	0.080	0.094	0.119	0.143
Sparisoma rubripinne	yellowtail parrotfish	Tropical; 42°N - 7°N				0.009	0.025	0.214	0.079		0.149	0.183	0.293	0.221	0.036
Sphoeroides spengleri	bandtail puffer	Subtropical; 43°N - 30°S	0.056	0.029		0.049	0.025	0.092	0.092	0.205	0.157	0.107	0.112	0.168	0.036
Euthynnus alletteratus	little tunny	Tropical; 56°N - 30°S	0.370	0.015	0.250	0.023	0.250	0.026	0.033		0.153		0.004		
Scarus guacamaia	rainbow parrotfish	Subtropical; 33°N - 41°S				0.003	0.675	0.071	0.020	0.023	0.016	0.095	0.041	0.090	0.036
Epinephelus morio	red grouper	Subtropical; 35°N - 27°S	0.009	0.029	0.063	0.035	0.025	0.041	0.138	0.295	0.044	0.134	0.034	0.136	0.071
Priacanthus arenatus	bigeye	Subtropical; 44°N - 40°S	0.139	0.132		0.026	0.033		0.013	0.659		0.009		0.014	
Sparisoma chrysopterum	redtail parrotfish	Tropical; 28°N - 4°N	0.037			0.020	0.050	0.117	0.039	0.068	0.065	0.040	0.140	0.193	0.071
Calamus proridens	littlehead porgy	Subtropical; 30°N - 18°N	0.046	0.265		0.209	0.108	0.020	0.020	0.091	0.008		0.016	0.035	
Monacanthus tuckeri	slender filefish	Subtropical; 37°N - 10°N	0.009			0.052		0.092	0.250	0.227	0.040	0.031	0.048	0.040	
Malacanthus plumieri	sand tilefish	Subtropical; 34°N - 8°S			0.063	0.096	0.067	0.061	0.053	0.227	0.016	0.031	0.108	0.059	
Hemiramphus brasiliensis	ballyhoo	Subtropical; 43°N - 13°S						0.638					0.124		
Gymnothorax moringa	spotted moray	Subtropical; 37°N - 26°S	0.037	0.015	0.188	0.023	0.025	0.082	0.132	0.136	0.012	0.009	0.020	0.055	
Lutjanus mahogoni	mahogany snapper	Subtropical; 36°N - 9°N					0.017	0.536			0.109	0.067	0.002		
Seriola zonata	banded rudderfish	Subtropical; 46°N - 23°S	0.704				0.017								
Coryphopterus dicrus	colon goby	Subtropical		0.029		0.023	0.067	0.469	0.039	0.023		0.027	0.020	0.011	
Scorpaena plumieri	spotted scorpionfish	Subtropical; 43°N - 30°S	0.167	0.074		0.029	0.067	0.056	0.079	0.068	0.036	0.042	0.039	0.034	
Parablennius marmoreus	seaweed blenny	Subtropical; 36°N - 27°S	0.056	0.118		0.096	0.008	0.020	0.020	0.114		0.213	0.002	0.016	
Epinephelus itajara	goliath grouper	Subtropical; 36°N - 33°S	0.463		0.188	0.003	0.008								
Holocentrus rufus	longspine squirrelfish	Tropical; 36°N - 28°S		0.029		0.038	0.083	0.036	0.039		0.073	0.049	0.041	0.130	0.143
Calamus leucosteus	whitebone porgy	Subtropical; 37°N - 10°N	0.009	0.544		0.035	0.017			0.045	0.008				
Apogon pseudomaculatus	twospot cardinalfish	Tropical; 43°N - 34°S	0.065	0.368	0.063	0.026	0.017	0.005	0.066				0.002	0.025	
Stephanolepis hispidus	planehead filefish	Subtropical; 46°N - 37°S		0.015		0.009		0.046	0.053	0.023	0.073	0.062	0.060	0.113	0.143
Chaetodon striatus	banded butterflyfish	Tropical; 41°N - 29°S				0.017	0.067	0.046	0.046	0.023	0.101	0.121	0.099	0.075	
Halichoeres poeyi	blackear wrasse	Tropical; 30°N - 33°S		0.029		0.201	0.233	0.041	0.013		0.056		0.004	0.009	
Scomberomorus maculatus	Spanish mackerel	Subtropical; 20°C - 30°C; 44°N - 19°N						0.041	0.020	0.023	0.020	0.112	0.369	0.002	
Heteroconger longissimus	brown garden eel	Tropical						0.367	0.086				0.071	0.052	
Scomberomorus regalis	cero	Tropical; 41°N - 24°S	0.037		0.125			0.051	0.053	0.023	0.089	0.055	0.105	0.027	
Stegastes adustus	dusky damselfish	Tropical; 32°N - 8°N	0.083			0.003	0.225	0.020			0.093	0.063	0.014	0.014	0.036
Heteropriacanthus cruentatus	glasseye snapper	Subtropical; 33°N - 32°S		0.029	0.063	0.122		0.005		0.250			0.014	0.007	
Halichoeres radiatus	puddingwife	Tropical; 23°C - 27°C; 37°N - 19°S		0.029	0.063	0.070	0.158	0.071			0.044	0.027	0.012	0.005	
Mycteroperca phenax	scamp	Subtropical; 41°N - 8°N	0.287	0.029			0.033		0.020		0.016	0.004	0.009	0.009	0.071
Rypticus maculatus	whitespotted soapfish	Subtropical; 41°N - 24°N	0.213	0.088		0.009	0.075	0.010	0.053			0.004			
Serranus subligarius	belted sandfish	#N/A	0.102	0.118	0.125	0.032	0.067								
Haemulon macrostoma	Spanish grunt	Tropical; 32°N - 34°S					0.192	0.046	0.013		0.081	0.016	0.012	0.005	0.071
Cephalopholis fulva	coney	Subtropical; 36°N - 28°S	0.019			0.006	0.058	0.087	0.013	0.045	0.040	0.042	0.012	0.036	0.071
Balistes vetula	queen triggerfish	Tropical; ? - 28°C; 43°N	0.037	0.088		0.023	0.067	0.010	0.013	0.068	0.024	0.009	0.007	0.011	0.071
Xyrichtys martinicensis	rosy razorfish	Subtropical				0.064	0.150		0.066	0.023	0.016	0.058	0.019	0.034	
Rypticus saponaceus	greater soapfish	Tropical; 33°N - 35°S	0.037	0.059		0.012	0.017	0.097	0.053	0.068	0.036	0.018	0.021	0.002	
Diplectrum formosum	sand perch	Subtropical; 42°N - 35°S	0.046	0.206		0.035	0.008		0.007	0.091	0.004		0.009	0.014	
Sphyraena barracuda	great barracuda	Subtropical; 42°N - 35°S	0.009			0.003	0.033	0.082	0.013		0.048		0.122	0.100	
Echeneis naucrates	sharksucker	Subtropical; 45°N - 45°S	0.019	0.029		0.009	0.042	0.036	0.007	0.091	0.073	0.027	0.032	0.043	
Calamus nodosus	knobbed porgy	Subtropical; 37°N - 18°N		0.074		0.076	0.108	0.010	0.059	0.045	0.008	0.004	0.007	0.005	
Cantherhines macrocerus	whitespotted filefish	Tropical; 33°N - 26°S				0.023	0.008	0.061	0.020		0.024	0.063	0.020	0.025	0.143
Malacoctenus triangulatus	saddled blenny	Subtropical; 32°N - 26°S	0.009	0.015	0.063	0.015	0.067	0.031	0.059	0.023	0.012	0.060	0.007	0.025	
Diodon holocanthus	balloonfish	Subtropical; 37°N - 39°S				0.006		0.020	0.053	0.045	0.060	0.058	0.067	0.044	
Scarus coeruleus	blue parrotfish	Tropical; 39°N - 24°S					0.008	0.097	0.013			0.103	0.055	0.025	
Rhomboplites aurorubens	vermilion snapper	Subtropical; 42°N - 33°S	0.222	0.074											
Apogon maculatus	flamefish	Tropical; 46°N - 7°N	0.009	0.191		0.006	0.042		0.013			0.009	0.005	0.002	
Scarus coelestinus	midnight parrotfish	Tropical; 34°N - 26°S					0.108	0.020	0.145				0.002	0.002	
Aluterus monoceros	unicorn filefish	Subtropical; 43°N - 39°S	0.019			0.035	0.025	0.015	0.026	0.023			0.129		
Haemulon album	margate	Tropical; 33°N - 33°S						0.020	0.013	0.023	0.012	0.094	0.032	0.039	0.036
Calamus bajonado	jolthead porgy	Subtropical; 42°N - 18°S		0.029		0.032	0.042	0.061	0.039		0.012	0.022	0.017	0.009	
Orthopristis chrysoptera	pigfish	Temperate; 41°N - 10°N	0.231								0.016			0.002	
Urobatis jamaicensis	yellow stingray	Tropical; 37°N - 7°N				0.012		0.005	0.026	0.023	0.024	0.046	0.027	0.086	
Canthidermis sufflamen	ocean triggerfish	Tropical; 43°N - 17°S	0.009	0.015		0.023	0.050	0.031	0.020		0.004	0.021	0.039	0.034	
Mycteroperca bonaci	black grouper	Subtropical; 33°N - 27°S				0.003	0.042	0.005	0.007		0.012	0.010	0.004	0.018	0.143
Epinephelus adscensionis	rock hind	Tropical; 40°N - 24°S						0.015			0.040	0.009	0.021	0.023	0.107
Fistularia tabacaria	bluespotted cornetfish	Tropical; 48°N - 29°S	0.019	0.029		0.009	0.067	0.026	0.020		0.020	0.013	0.005	0.005	
Mycteroperca microlepis	gag	Subtropical; 40°N - 27°S	0.111				0.042			0.023	0.004	0.018	0.009	0.005	
Caranx hippos	crevalle jack	Subtropical		0.029				0.020	0.033	0.114			0.014		
Epinephelus guttatus	red hind	Tropical; 36°N - 8°N				0.026	0.017	0.020	0.020	0.091	0.008	0.004	0.016	0.005	
Sphyraena borealis	southern sennet	Tropical; 33°N - 35°S						0.204							
Halichoeres pictus	rainbow wrasse	Tropical; 32°N - 9°N				0.003	0.117	0.036	0.039				0.007		
Serranus annularis	orangeback bass	Tropical; 32°N -			0.063	0.017	0.058	0.005		0.045			0.002		
Mulloidichthys martinicus	yellow goatfish	Subtropical; 33°N - 26°S					0.117	0.015				0.036	0.004	0.009	
Synodus intermedius	sand diver	Subtropical; 37°N - 30°S	0.019			0.047		0.010	0.007	0.045	0.016	0.009	0.002	0.020	
Gymnothorax funebris	green moray	Tropical; 42°N - 30°S	0.028			0.006	0.017	0.041		0.023	0.020	0.022	0.018		
Aluterus schoepfii	orange filefish	Subtropical; 46°N - 27°S			0.063	0.035		0.036			0.008	0.004	0.021		
Scarus vetula	queen parrotfish	Tropical; 33°N - 8°N					0.017	0.020	0.007		0.036	0.027	0.030	0.025	
Xyrichtys novacula	pearly razorfish	Subtropical		0.015		0.020			0.086		0.008		0.002	0.027	
Holocanthus sp.	townsend angelfish		0.009			0.026	0.025	0.015		0.023	0.016	0.004	0.020	0.012	
Gymnothorax miliaris	goldentail moray	Subtropical; 41°N - 26°S				0.006	0.008	0.010	0.013		0.016	0.013	0.005	0.005	0.071
Hypoplectrus puella	barred hamlet	Tropical				0.006	0.033	0.026	0.007		0.032	0.009	0.007	0.020	
Odontoscion dentex	reef croaker	Tropical; 22°N - 25°S	0.056	0.074							0.008		0.002		
Malacoctenus macropus	rosy blenny	Subtropical; 32°N -		0.044		0.029	0.058				0.004				
Scartella cristata	molly miller	Tropical	0.009							0.114					
Ginglymostoma cirratum	nurse shark	Subtropical; 44°N - 35°S	0.009			0.006	0.017	0.005					0.014		0.071
Lactophrys bicaudalis	spotted trunkfish	Subtropical; 32°N - 35°S				0.012	0.017	0.015			0.044	0.009	0.005	0.009	
Centropomus undecimalis	common snook	Tropical; 25°C - 31°C	0.111												
Hypoplectrus gemma	blue hamlet	Tropical						0.010	0.007		0.012	0.058	0.005	0.018	
Apogon townsendi	belted cardinalfish	Tropical					0.008				0.016	0.004	0.009		0.071
Lagodon rhomboides	pinfish	Subtropical; 42°N - 18°N									0.101				
Microspathodon chrysurus	yellowtail damselfish	Subtropical; 33°N - 25°S		0.015				0.015	0.053		0.004	0.013			
Synodus foetens	inshore lizardfish	Tropical; 25°C - 35°C; 43°N - 29°S	0.019				0.008	0.005	0.007		0.008	0.009	0.002	0.005	0.036
Bothus ocellatus	eyed flounder	Tropical; 41°N - 33°S							0.092						
Serranus phoebe	tattler	Subtropical; 33°N - 34°S	0.019		0.063	0.009									
Halichoeres caudalis	painted wrasse	Subtropical; 37°N - 6°N			0.063							0.018			
Eques punctatus	spotted drum	Tropical; 36°N - 24°S	0.019					0.015			0.008	0.004	0.021	0.005	
Opistognathus whitehursti	dusky jawfish	Tropical; 32°N - 7°N								0.068			0.004		
Nicholsina usta	emerald parrotfish	Tropical; 41°N - 30°S				0.041		0.005		0.023				0.002	
Remora remora	remora	Subtropical; 60°N - 36°S		0.029		0.006					0.016	0.004	0.011	0.002	
Aetobatus narinari	spotted eagle ray	Subtropical; 36°N - 24°S						0.010			0.016			0.005	0.036
Bothus lunatus	peacock flounder	Tropical; 34°N - 25°S			0.063	0.003									
Stegastes diencaeus	longfin damselfish	Tropical						0.015	0.013		0.008	0.018	0.011		
Elacatinus xanthiprora	yellowprow goby	Subtropical; 37°N - 8°N	0.056										0.009		
Carcharhinus perezii	reef shark	Subtropical; 33°N - 35°S					0.058						0.004		
Carcharhinus leucas	bull shark	Subtropical; 42°N - 39°S						0.026	0.013	0.023					
Myrichthys breviceps	sharptail eel	Subtropical; 33°N - 25°S				0.009	0.025		0.013		0.004		0.002	0.007	
Caranx latus	horse-eye jack	Subtropical; 41°N - 23°S	0.028											0.030	
Apogon binotatus	barred cardinalfish	Tropical; 32°N - 9°N				0.003		0.005	0.013			0.022	0.005	0.008	
Diodon hystrix	porcupinefish	Subtropical; 35°N - 31°S				0.006		0.010			0.016	0.004	0.007	0.011	
Trachurus lathami	rough scad	Subtropical; 45°N - 37°S						0.051							
Coryphopterus eidolon	pallid goby	Tropical		0.015		0.012	0.008		0.013						
Malacoctenus aurolineatus	goldline blenny	Subtropical												0.045	
Opsanus tau	oyster toadfish	Subtropical; 43°N - 25°N		0.044											
Scomberomorus cavalla	king mackerel	Tropical; 44°N - 22°S	0.019			0.003		0.005	0.013		0.004				
Elacatinus evelynae	sharknose goby	Tropical; 22°C - 27°C										0.027		0.016	
Elacatinus horsti	yellowline goby	Tropical; - 9°N						0.020			0.012	0.009			
Hypanus americanus	southern stingray	Subtropical; 36°N - 4°S	0.009				0.017		0.007		0.008				
Microgobius carri	Seminole goby	Subtropical; 37°N - 10°N		0.029							0.004		0.007		
Neoniphon coruscum	reef squirrelfish	Tropical; 33°N - 7°N				0.009	0.025	0.005							
Emblemaria pandionis	sailfin blenny	Tropical; 32°N - 4°N				0.006		0.005				0.009	0.005	0.014	
Lactophrys trigonus	trunkfish	Subtropical; 45°N - 34°S				0.003	0.008					0.013	0.004	0.009	
Paranthias furcifer	Atlantic creolefish	Subtropical; 35°N - 28°S				0.035									
Eques lanceolatus	jackknife-fish	Tropical; 37°N - 34°S	0.019			0.012							0.004		
Upeneus parvus	dwarf goatfish	Tropical; 37°N - 33°S					0.033								
Lutjanus jocu	dog snapper	Subtropical; 42°N - 26°S	0.009					0.005			0.004		0.004	0.009	
Labrisomus nuchipinnis	hairy blenny	Tropical; 34°N - 34°S	0.028												
Centropristis ocyurus	bank sea bass	Subtropical; 37°N - 18°N	0.028												
Echeneis neucratoides	whitefin sharksucker	Subtropical; 43°N - 4°N	0.019					0.005					0.004		
Gymnothorax vicinus	purplemouth moray	Subtropical				0.006		0.005				0.010	0.002	0.002	
Ctenogobius saepepallens	dash goby	Tropical; - 9°N										0.022			
Hypleurochilus bermudensis	barred blenny	Tropical; 33°N - 9°N				0.003		0.005			0.008	0.004			
Alectis ciliaris	African pompano	Subtropical; 42°N - 34°S						0.020							
Amblycirrhitus pinos	redspotted hawkfish	Tropical; 36°N - 24°S					0.008	0.005	0.007						
Lutjanus cyanopterus	cubera snapper	Subtropical; 46°N - 26°S	0.019												
Archosargus rhomboidalis	sea bream	Subtropical; 22°C - 30°C; 41°N - 58°S										0.018			
Oligoplites saurus	leatherjack	Subtropical; 43°N - 35°S									0.016				
Rachycentron canadum	cobia	Subtropical; 17°C - 32°C; 47°N - 37°S				0.003					0.012				
Gerres cinereus	yellowfin mojarra	Subtropical; 33°N - 23°S							0.013						
Chilomycterus schoepfii	striped burrfish	Tropical; 46°N - 5°S									0.008	0.004			
Pseudobatos lentiginosus	Atlantic guitarfish	Subtropical; 36°N - 18°N				0.006			0.007						
Sphyrna lewini	scalloped hammerhead	Tropical; 46°N - 39°S				0.012									
Hypoplectrus randallorum	tan hamlet							0.005				0.004	0.002		
Enchelycore nigricans	viper moray	Subtropical										0.009	0.002		
Liopropoma rubre	peppermint basslet	Tropical											0.011		
Sphoeroides testudineus	checkered puffer	Subtropical; 42°N - 35°S				0.006								0.005	
Liopropoma eukrines	wrasse basslet	Subtropical; 37°N - 24°N	0.009												
Lutjanus campechanus	red snapper	Subtropical	0.009												
Sphoeroides nephelus	southern puffer	Subtropical; 31°N - 9°N	0.009												
Prognathodes aculeatus	longsnout butterflyfish	Tropical; 33°N - 9°S						0.005			0.004				
Diplodus argenteus	silver porgy	Subtropical; 20°S - 35°S										0.009			
Dactylopterus volitans	flying gurnard	Subtropical; 52°N - 45°S										0.009			
Alphestes afer	mutton hamlet	Tropical; 32°N - 27°S					0.008								
Negaprion brevirostris	lemon shark	Subtropical; 45°N - 33°S				0.003					0.004				
Trachinotus falcatus	permit	Subtropical; 42°N - 25°S						0.005					0.002		
Rypticus bistrispinus	freckled soapfish	Tropical; - 23°S							0.007						
Prionotus ophryas	bandtail searobin	Subtropical; 37°N - 9°N									0.004			0.002	
Opistognathus macrognathus	banded jawfish	Tropical											0.004	0.002	
Ophioblennius macclurei	redlip blenny	Tropical				0.006									
Hypoplectrus guttavarius	shy hamlet	Tropical									0.004		0.002		
Monacanthus ciliatus	fringed filefish	Subtropical; 52°N - 34°S				0.003							0.002		
Prionotus rubio	blackwing searobin	Subtropical; 37°N - 19°N										0.004			
Chilomycterus reticulatus	spotted burrfish	Subtropical; - 24°S										0.004			
Megalops atlanticus	tarpon	Subtropical; 10°C - 40°C; 49°N - 44°S									0.004				
Neoniphon vexillarium	dusky squirrelfish	Tropical											0.004		
Chilomycterus antennatus	bridled burrfish	Tropical											0.004		
Hemiemblemaria simula	wrasse blenny	Tropical											0.004		
Coryphopterus lipernes	peppermint goby	Tropical; 25°N				0.003									
Enchelycore carychroa	chestnut moray	Tropical				0.003									
Galeocerdo cuvier	tiger shark	Subtropical; 62°N - 44°S				0.003									
Istiophorus platypterus	sailfish	Subtropical				0.003									
Ogcocephalus nasutus	shortnose batfish	Tropical				0.003									
Priolepis hipoliti	rusty goby	Tropical				0.003									
Acanthemblemaria maria	secretary blenny	Tropical; 18°N - 10°N												0.002	
Apogon quadrisquamatus	sawcheek cardinalfish	Tropical; - 24°S												0.002	
Melichthys niger	black durgon	Tropical; 30°N - 29°S											0.002		
			139	101	77	179	165	184	157	121	174	172	194	172	85

Mean fish per SSU
>10
9
8
7
6
5
4
3
2
1
0

Present only in South Plam Beach, Deerfield, and Broward-Miami

Present only in North Plam Beach and Martin
